# Water Oxidation by Pentapyridyl Base Metal Complexes? A Case Study

**DOI:** 10.1021/acs.inorgchem.2c00631

**Published:** 2022-06-06

**Authors:** Manuel Boniolo, Md Kamal Hossain, Petko Chernev, Nina F. Suremann, Philipp A. Heizmann, Amanda S.L. Lyvik, Paul Beyer, Michael Haumann, Ping Huang, Nessima Salhi, Mun Hon Cheah, Sergii I. Shylin, Marcus Lundberg, Anders Thapper, Johannes Messinger

**Affiliations:** † Molecular Biomimetics, Department of Chemistry−Ångström Laboratory, Uppsala University, 75120 Uppsala, Sweden; ‡ Synthetic Molecular Chemistry, Department of Chemistry−Ångström Laboratory, Uppsala University, 75120 Uppsala, Sweden; § Physics Department, Freie Universität Berlin, 14195 Berlin, Germany; ∥ Department of Chemistry, Chemical Biological Centre, Umeå University, 90187 Umeå, Sweden

## Abstract

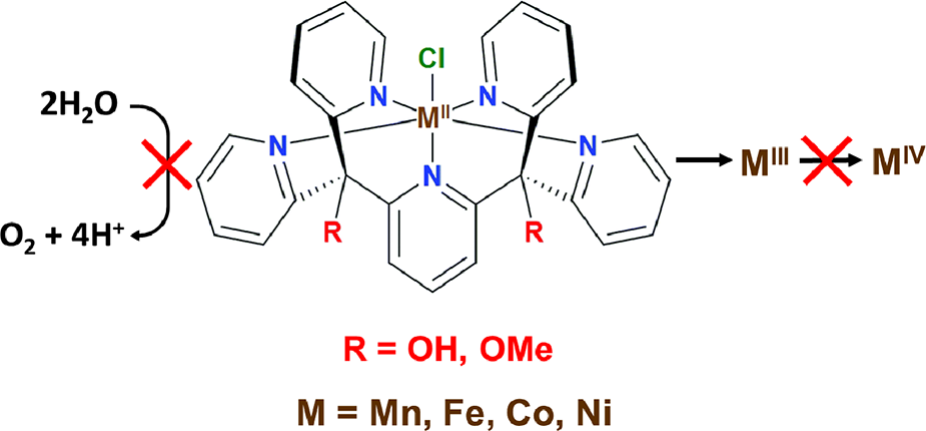

The design of molecular water oxidation catalysts (WOCs) requires a rational approach that considers the intermediate steps of the catalytic cycle, including water binding, deprotonation, storage of oxidizing equivalents, O–O bond formation, and O_2_ release. We investigated several of these properties for a series of base metal complexes (M = Mn, Fe, Co, Ni) bearing two variants of a pentapyridyl ligand framework, of which some were reported previously to be active WOCs. We found that only [Fe(Py5**OMe**)Cl]^+^ (Py5**OMe** = pyridine-2,6-diylbis[di-(pyridin-2-yl)methoxymethane]) showed an appreciable catalytic activity with a turnover number (TON) = 130 in light-driven experiments using the [Ru(bpy)_3_]^2+^/S_2_O_8_^2–^ system at pH 8.0, but that activity is demonstrated to arise from the rapid degradation in the buffered solution leading to the formation of catalytically active amorphous iron oxide/hydroxide (FeOOH), which subsequently lost the catalytic activity by forming more extensive and structured FeOOH species. The detailed analysis of the redox and water-binding properties employing electrochemistry, X-ray absorption spectroscopy (XAS), UV–vis spectroscopy, and density-functional theory (DFT) showed that all complexes were able to undergo the M^III^/M^II^ oxidation, but none was able to yield a detectable amount of a M^IV^ state in our potential window (up to +2 V vs SHE). This inability was traced to (i) the preference for binding Cl^–^ or acetonitrile instead of water-derived species in the apical position, which excludes redox leveling *via* proton coupled electron transfer, and (ii) the lack of sigma donor ligands that would stabilize oxidation states beyond M^III^. On that basis, design features for next-generation molecular WOCs are suggested.

## Abstract 2

We scrutinize the water oxidation activity for pentapyridyl metal complexes [M^II^(Py5R)Cl]^+^ (M = Mn, Fe, Co, Ni; R = OH, OMe). Analysis of their stability, redox, and water-binding properties shows that the complexes are not able to reach high-valent intermediate states and do not catalyze water oxidation in their molecular form.

## 1 Introduction

Water is the only essentially inexhaustible source for electrons and protons on earth, and for that reason, its light-driven oxidation has been exploited by nature for the reduction of carbon dioxide to carbohydrates for the past 3 billion years.^1−4^ Thereby, oxygenic photosynthesis continues to provide the chemical energy for life on earth and the molecular oxygen we breathe. More recently, it has been realized that adapting this process into technical solutions that produce H_2_ or drive CO_2_ or N_2_ reduction will be required for replacing the use of the ancient photosynthetic products such as coal, oil, and gas by CO_2_-neutral alternatives.^5−7^

The present technical solutions for CO_2_-neutral water oxidation are based on electrolysis powered by locally or remotely produced renewable electricity. While functional, widespread implementation is hampered by its low metal atom economy and high system costs. Thus, research efforts are ongoing to reduce the amount of metal atoms required for catalysis by developing efficient and stable molecular water oxidation catalysts (WOCs).^8,9^ These may be employed to either improve photovoltaic-driven electrolysis or allow the construction of direct (wireless) photochemical processes.

The first example of a molecular catalyst for water oxidation was the blue dimer, cis-[(H_2_O)Ru^III^(bpy)_2_(μ-O)Ru^III^(bpy)_2_(OH_2_)]^4+^, reported in 1982 by Meyer et al.^10^ The activity of molecular ruthenium-based catalysts has been improved over the past 40 years in a spectacular fashion by systematic studies combining electrochemistry, spectroscopy, isotope ratio mass spectrometry, and DFT calculations with synthetic efforts, as summarized recently in two landmark reviews.^11,12^ One important step in this development was the realization that mononuclear Ru complexes, which can be more readily synthetized than dimers, can also catalyze water oxidation either via water nucleophilic attack (WNA) or by the intermolecular coupling of two oxo units (I2M). The present record activities of these mononuclear Ru complexes are a turnover number (TON) over 100,000 and a turnover frequency (TOF) up to 1000 s^–1^,^13^ which both rival the performance of biological water oxidation.^14^ Similarly, several WOCs with Ir as the active centers have been developed.^15−18^

Inspired by the tetra-manganese calcium penta-oxygen (Mn_4_CaO_5_) cluster of natural photosynthesis, attempts are ongoing for developing sustainable and stable molecular base metal WOCs that utilize earth-abundant first-row transition metals. Extensive efforts have been devoted to develop molecular WOCs based on manganese,^19^ iron,^20,21^ cobalt,^22,23^ nickel,^24^ and copper.^25^ Notable examples are dinuclear Mn^II^Mn^III^ complexes with benzodiazole derivatives,^26^ a pentanuclear Fe_4_^II^Fe^III^ complex with 3,5-bis(2-pyridyl)pyrazole ligands,^27^ iron complexes with the tetradentate Me2Pytacn (1-(2′-pyridylmethyl)-4,7-dimethyl-1,4,7-triazacyclononane) ligand,^28^ a bipyridyl hydroxy-bridged Cu^II^ dimer,^29^ a bispyridylpyrazolate ligated Co^III^ peroxo dimer,^30^ and a water-soluble Ni^II^ porphyrin.^31^ However, it is challenging to prove, beyond doubt, the molecular nature of the active catalysts.^32,33^ Indeed, careful studies have shown in several cases that, under water oxidation conditions, the formation of catalytically active heterogeneous metal-oxide species occurred, while the molecular species was just an inactive or less active precursor.^34−36^ Thus, similar to the Ru complexes,^11,12^ careful systematic studies are required for improving the TOF and TON of molecular base metal complexes. For this purpose, mononuclear complexes are an ideal starting point.

**Scheme 1:**
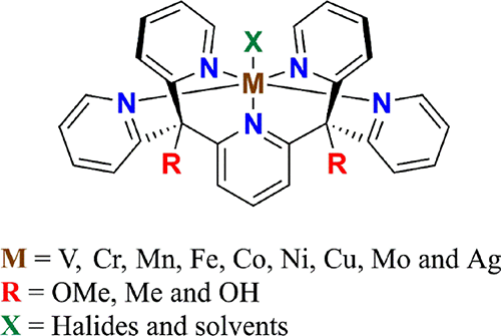
Molecular Structure of the Py5-Metal Complexes Reported to Date^37−49,51−53^

Pentapyridyl ligands of the Py5 family (Py5 = 2,6-bis-[(2-pyridyl)methyl]pyridine) have been employed in the past 20 years as a robust but flexible scaffold for accommodating different transition metals in a variety of oxidation states and with different apical ligands (X in Scheme 1).^37−45^ In addition, there are three reported variations of the peripheral group R of the Py5 scaffold: hydroxyl-substituted (Py5**OH**, pyridine-2,6-diylbis[di-(pyridin-2-yl)methanol]), methoxyl-substituted (Py5**OMe**, pyridine-2,6-diylbis[di-(pyridin-2-yl)methoxymethane]), and methyl-substituted (Py5**Me**, 2,6-bis(1,1-bis(2-pyridyl)ethyl)pyridine). Comprehensive comparative studies have been made to highlight the structural and electronic differences of the complexes having the same ligand but different divalent metals.^44−46^ The peripheral ligand modification is not innocent, as it affects metal–ligand distances, the binding strength of the apical ligand, and even the spin state of the metal ion. For example, [Fe(Py5**OH**)Cl]^+^ exhibits a thermally induced spin transition at 80 K, while [Fe(Py5**OMe**)Cl]^+^ remains high-spin in the entire temperature range.^47^

Berlinguette et al. were the first to employ [Co(Py5**OMe**)H_2_O]^2+^ in electrochemical water oxidation at pH 9.2,^48,49^ but the molecular nature of the active species has been called into question.^50^ Subsequently, Sun et al. reported oxygen evolution from water in a controlled potential electrolysis (CPE) experiment using [Ni(Py5**Me**)Cl]^+^ at pH 10.^51^ Also [Co(Py5**OH**)Cl]^+^ and [Fe(Py5**OH**)Cl]^+^ complexes were claimed to be active WOCs in photochemical water oxidation at pH 8.0.^52,53^ In the related ligand systems featuring four and three pyridine rings, photochemical and chemical water oxidation was reported for [Fe(N4Py)]^2+^ (N4Py = *N*,*N*-bis(2-pyridylmethyl)-*N*-bis(2-pyridyl)methylamine), [Mn(N4Py)]^2+^, and [Mn(PaPy3)]^2+^ (PaPy3 = *N*,*N*-bis(2-pyridylmethyl)-amine-*N*-ethyl-2-pyridine-2-carboxamide), while [Mn(Py5**OMe**)H_2_O]^2+^ appeared to be inactive.^54,19^

Even without a detailed knowledge of the reaction mechanism for water oxidation, it is still possible to state some general requirements for activity. The redox potential of water under standard conditions (pH = 0; *T* = 298 K) is *E*^o^ = 1.23 V, which is lowered to *E*_H2O/O2_ = 0.76 V at pH = 8.0 due to the release of four protons. To drive the four-electron four-proton removal with appreciable kinetics, the redox potential of the catalyst shall be somewhat more oxidizing than this potential but not too high so that the process can still be driven efficiently by visible light. For any feasible mechanism leading to O_2_ formation, at least a two-electron oxidation of the WOC needs to be achieved. If the M^II^ state is most stable, as reported for the pentapyridyl complexes above,^52,53^ this implies that at least the equivalent of a M^IV^ state needs to be reached. If the WOCs have a water ligand and can form M^IV^=O, the most straightforward reaction mechanism for O–O bond formation is I2M, unless steric clashes prevent the I2M mechanism. In such cases, WNA onto the M^IV^=O unit may also be possible but would need to be followed, within the lifetime of the peroxidic intermediate, by the removal of two further electrons. Thus, pentapyridyl base metal complexes would need to support at least two oxidation steps within a feasible potential window and additionally bind one water molecule that would both act as a substrate and allow redox leveling via concerted or sequential PCET.

Recently, we have reported the crystal structure and electrochemical properties, in dry acetonitrile (MeCN), of [M(Py5**OH**)X]^*n*+^ (M = Mn, Fe, Co, Ni; X = Cl^–^ or MeCN).^46^ We identified the electron-spin energetics as the main contributor to the relative redox potentials of the metal-centered one-electron oxidations within this 3d metal series. In addition, we noted the effects of organic solvent and ionic strength on the relative binding affinities of the apical ligand. The complexes do not have a water ligand as isolated but instead complete their ligand sphere with an apical Cl^–^/MeCN ligand that, potentially, can be exchanged against water/hydroxide in water-containing media.

Here, we study the effects of water addition on the structure and redox properties of these complexes and scrutinize the reported water oxidation activity.^52,53^ For elucidating the effects of peripheral ligand changes, we additionally include the [Fe(Py5**OMe**)Cl]^+^ complex. We show that, at pH 8.0 (borate buffer), no complex of this series worked as a molecular WOC using the ruthenium dye photo-oxidant system and that the highest TON ≈ 130 found here for [Fe(Py5**OMe**)Cl]^+^ is explained by its low stability in aqueous solutions, which led to rapid FeOOH formation. By contrast, for [Fe(Py5**OH**)Cl]^+^ that has a significantly higher stability, a TON = 2 was found. The inability of the [M(Py5**OH**)X]^*n*+^ and [Fe(Py5**OMe**)Cl]^+^ complexes to split water in MeCN/H_2_O mixtures is explained by two factors: (i) the preference for exchanging Cl^–^ by MeCN rather than water or hydroxide in their M^II^ state and (ii) the inability to undergo M^IV/III^ oxidation. Together, this leads to the inability to form M^IV^=O or M^III^–O^•^ species at relevant electrochemical potentials, here taken to be up to 2 V vs SHE (all potentials are hereinafter given vs SHE, standard hydrogen electrode). We conclude that the underlying reasons are that, first, the ligand system is neutral and thus does not provide enough stabilization for highly positively charged intermediates and, second, it lacks design features that would promote water binding and redox leveling via concerted or sequential proton coupled electron transfer (PCET).

## 2 Results and Discussion

### 2.1 Water Oxidation Assays

The water oxidation catalysis by pentapyridyl base metal complexes was evaluated in an aqueous borate buffer at pH 8.0 containing a low amount acetonitrile (0.2% MeCN) for enhancing the solubility of the complexes. We performed both light-driven and chemical oxidation measurements using [Ru(bpy)_3_]^2+^/S_2_O_8_^2–^ and [Ru(bpy)_3_]^3+^, respectively, as well as electrochemical oxidation (10% MeCN) *via* controlled potential electrolysis (CPE).

In the light-driven assays with the [M(Py5**OH**)Cl]^+^ complexes, in all cases, amounts of O_2_ evolved were comparable to those obtained in corresponding blank experiments (Table 1). By contrast, with the Fe complex featuring the methylated ligand, [Fe(Py5**OMe**)Cl]^+^, a clear water oxidation activity was seen that corresponded to an average TON of 133. Similarly, the chemical oxidation of [Fe(Py5**OMe**)Cl]^+^ with 60 equiv of [Ru(bpy)_3_]^3+^ resulted in a TON of 8, corresponding to 50% of the maximal possible O_2_ production (Figure S1).

**Table 1:** Light-Driven (LED, 470 nm) Oxygen Evolution Using 10 μM [M(Py5**OH**)Cl]^+^ or 1.25 μM [Fe(Py5**OMe**)Cl]^+^, 0.5 mM [Ru(bpy)_3_](ClO_4_)_2_ as the Photosensitizer, and 2.5 mM Na_2_S_2_O_8_ as the Electron Acceptor in 40 mM pH 8.0 Borate Buffer and 0.2% v/v MeCN Measured with TR-MIMS (Time-Resolved Membrane-Inlet Mass Spectrometry)

complex	TON
[Mn(Py5**OH**)Cl]^+^	1.3 ± 0.9
[Fe(Py5**OH**)Cl]^+^	2.1 ± 0.7
[Fe(Py5**OMe**)Cl]^+^	133 ± 4
[Co(Py5**OH**)Cl]^+^	1.6 ± 0.6
[Ni(Py5**OH**)Cl]^+^	–0.5 ± 0.5

Water oxidation using [Fe(Py5**OMe**)Cl]^+^ was investigated further in a CPE experiment. A potential of at least 2.0 V was required for generating O_2_ detectible by a Clark-type electrode. Under these conditions, O_2_ evolution was observed over the first 6–8 min of CPE with a maximal Faradaic efficiency close to 70% after 3 min. Thereafter, O_2_ evolution ceased, indicating the instability of the catalyst (Figures S2 and S3). While XPS analysis of the working electrode performed after the 20 min CPE established the deposition of iron (Figure S4; Tables S1 and S2), a rinse test demonstrated that the deposit was inactive in oxygen evolution (Figure S5).

To understand whether [Fe(Py5**OMe**)Cl]^+^ is the catalyst in the above assays or rather a precatalyst that decomposes into a metastable, catalytically active species, we studied the stability of [Fe(Py5**OMe**)Cl]^+^ in 90 mM borate buffer (pH 8, 10% MeCN; exposed to air) by UV–vis and X-ray spectroscopies. The UV–vis data revealed that [Fe(Py5**OMe**)Cl]^+^ decomposes completely within 15 min, while the degradation of the ″less active″ [Fe(Py5**OH**)Cl]^+^ is not complete until 2 h (Figure S6).

**Figure 1:**
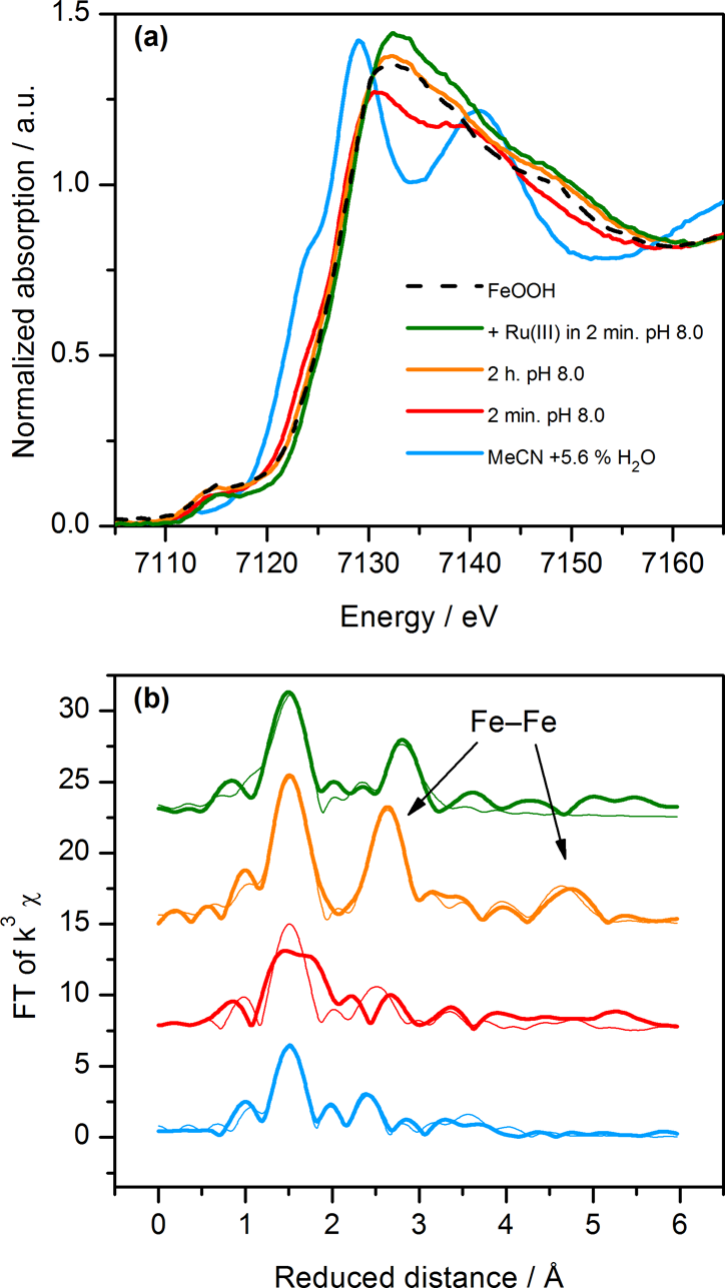
(a) XANES and (b) FT-EXAFS data of 0.5 mM [Fe(Py5**OMe**)Cl]^+^ recorded at 20 K under different conditions: dissolved in MeCN with 5.6% v/v water and 0.1 M TBAPF_6_, blue line; after 2 min as dissolved in borate buffer (90 mM, pH 8.0, with 10% MeCN), red line; after 2 h in borate buffer (90 mM, pH 8.0, with 10% MeCN), orange line; oxidized with 6 equiv of [Ru(bpy)_3_](ClO_4_)_3_ after 2 min as dissolved in borate buffer (90 mM, pH 8.0, with 10% MeCN), green line; and solvothermally prepared FeOOH, black dashed line. Simulations of the experimental data are shown as thin lines.

The XAS measurements (Figure 1) showed that the spectral change observed by UV–vis is due to a rapid Fe^II^ oxidation to Fe^III^ coupled to FeOOH formation. A 2 min old solution of [Fe(Py5**OMe**)Cl]^+^ in borate buffer already displayed an edge shift of 2 eV with respect to the spectrum recorded in MeCN with 5.6% v/v water (Figure 1a). This shift increased to 2.5 eV for the 2 h sample, and a similar shift was also seen after oxidation with [Ru(bpy)_3_]^3+^. These final edge position and shape are virtually identical to those of solvothermally prepared Fe(III) oxide/hydroxide (FeOOH) and to those of minerals such as goethite.^55^ The EXAFS of the 2 min sample (Figure S7) is characterized by low intensity peaks, corresponding to a sample that is a mixture of phases, at least one of which is an oxide/hydroxide. However, in the 2 h sample, the EXAFS shows strong oscillations at high wavenumbers, and the two prominent peaks in the Fourier transform (Figure 1b) correspond to Fe–O distances of 1.93 Å and Fe–Fe of 2.99 Å. Additionally, long-distance Fe–Fe peaks are seen up to 5 Å. These long-range features in the EXAFS indicate that, at this stage, a well-ordered and more extended FeOOH structure is reached. After chemical oxidation with [Ru(bpy)_3_]^3+^, a similar FeOOH type spectrum was obtained in the EXAFS, but the dominating Fe–Fe distance was 3.13 Å (Figure S7).

**Figure 2:**
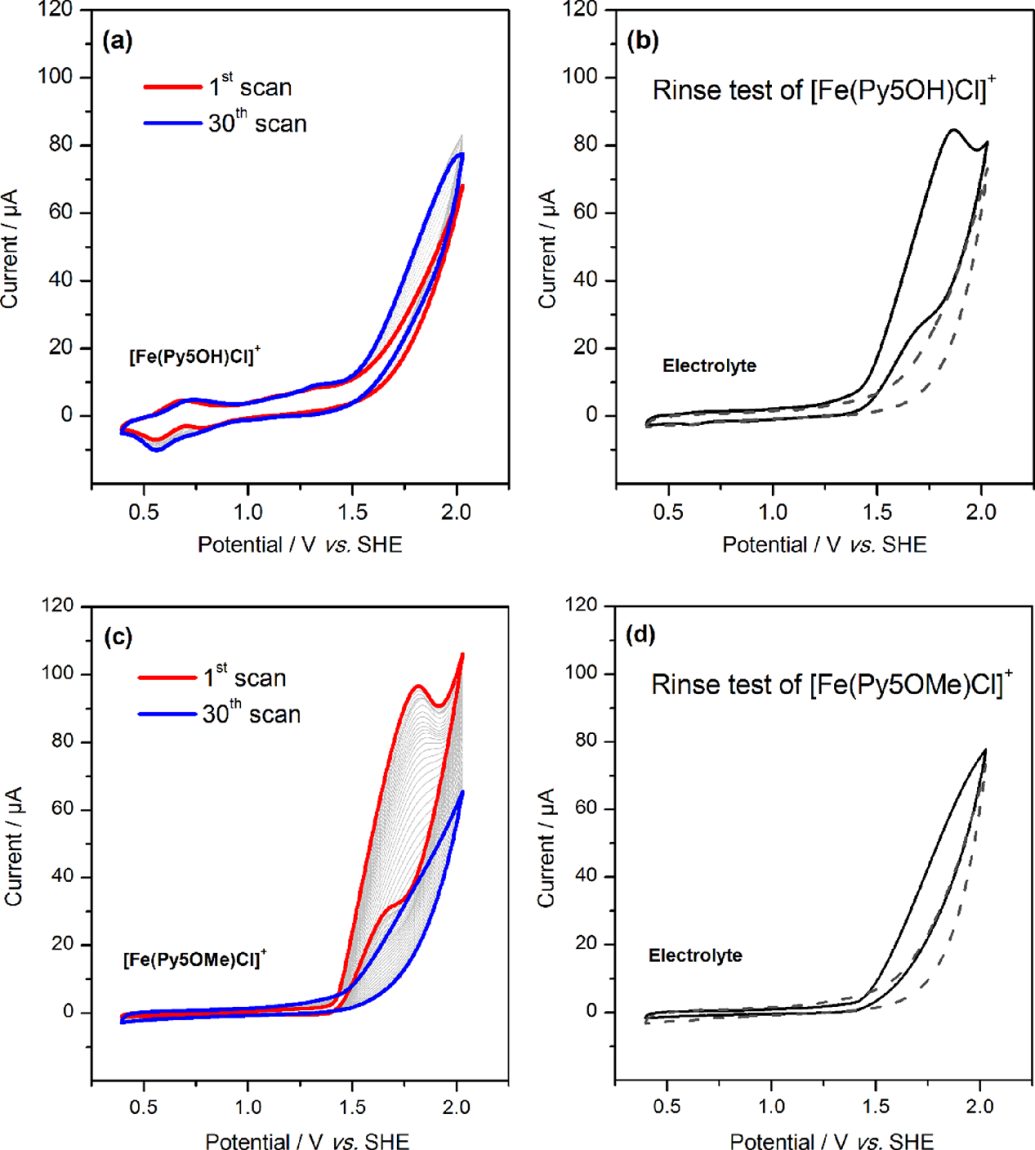
CVs of 0.5 mM [Fe(Py5**OH**)Cl]^+^ and [Fe(Py5**OMe**)Cl]^+^ recorded in 90 mM borate buffer (pH 8.0) with 10% acetonitrile: (a) multiple CV scans of [Fe(Py5**OH**)Cl]^+^. The large peak-to-peak separation of 140 mV for the reversible oxidation at 0.63 V is due to the high solution resistance as tested by using [Fe(CN)_6_]^2+^ as the internal standard. (b) CV of the electrolyte solution with the unpolished working electrode after the experiment shown in panel a. (c) Multiple CV scans of [Fe(Py5**OMe**)Cl]^+^. (d) CV of the electrolyte solution with the unpolished working electrode after the experiment in panel c. Scan rate 100 mV s^–1^. The CVs of the respective blank experiments with polished electrodes are represented with dashed lines.

Further evidence for the rapid loss of the molecular [Fe(Py5**OMe**)Cl]^+^ species under both chemical and electrochemical water oxidation conditions was obtained by both dynamic light scattering (DLS) and multiple cyclic voltammogram (CV) scans. At sample concentrations of 0.5 mM, DLS clearly showed particle formation upon aging in air and after [Ru(bpy)_3_]^3+^ addition, while the method was not sensitive enough at the concentrations employed for the chemical and photochemical water oxidation assays (Table S3). The CVs of [Fe(Py5**OMe**)Cl]^+^ in borate lack the reversible features from the molecular species observed in acetonitrile, while this feature is clearly observed for [Fe(Py5**OH**)Cl]^+^ (compare Figure 2a,c). Interestingly, while for [Fe(Py5**OMe**)Cl]^+^ the water oxidation wave around 1.7 V is pronounced in the first scan, it decreases strongly with scan number. By contrast, the behavior is opposite for [Fe(Py5**OH**)Cl]^+^, where the wave increases with scan number. A rinse test after the 30^th^ scan revealed an electrocatalytically active material on the electrode in both cases, but the deposit of the [Fe(Py5**OH**)Cl]^+^ precursor was more active. It is thus concluded that the introduction of the methoxy groups in Py5**OMe** destabilizes the pentapyridyl complex to an extent that [Fe(Py5**OMe**)Cl]^+^ decomposes rapidly at pH 8.0 to form amorphous FeOOH that acts as a good water oxidation catalyst but which loses catalytic activity upon forming a more structured FeOOH precipitate during aging.^56^ The decomposition process is much slower in [Fe(Py5**OH**)Cl]^+^, explaining the very low TON in the chemical oxidation, where the limited stability of the Ru oxidant allows sampling of the initial condition only. Similarly, it is consistent with the presence of a molecular species and a modest initial water oxidation wave for [Fe(Py5**OH**)Cl]^+^ that slowly increases with scan number. The molecular basis for this surprisingly strong effect of a ligand variation in a remote position lies in a 30° tilt of the axial pyridine ligand that is induced by the two methoxy groups, which in turn leads to a nonsymmetrical pyridine coordination in the equatorial plane and poorer overlap of metal–ligand orbitals (Table S4 and Figure S8).^47^

Thus, none of the pentapyridyl base metal complexes studied here acted as a molecular WOC. Below, we describe detailed electrochemical, UV–vis, and X-ray absorption experiments as well as DFT calculations that elucidate the underlying reasons for that.

### 2.2 Oxidation Intermediates

As mentioned in the Introduction, for the pentapyridyl base metal complexes to function as water oxidation catalysts, at least two oxidation steps would need to be reached within a feasible oxidation potential. Additionally, at some point during the reaction, they should bind a water-derived ligand that can act as a substrate and allow redox leveling via PCET. The pentapyridyl complexes studied here do not have a water ligand as isolated but instead complete their ligand sphere with an apical Cl^–^ ligand or solvent molecule that, potentially, can be exchanged against water/hydroxide in water-containing media. We will test the latter at a low water concentration (5.6% v/v; about 3 M water) in MeCN, because, as seen above, high water concentrations can lead to complete ligand exchange with concomitant oxide formation, at least for the two Fe complexes. As ligand affinity changes with oxidation state, a detailed characterization is performed for all species detected in the CVs up to 2.0 V.

**Figure 3:**
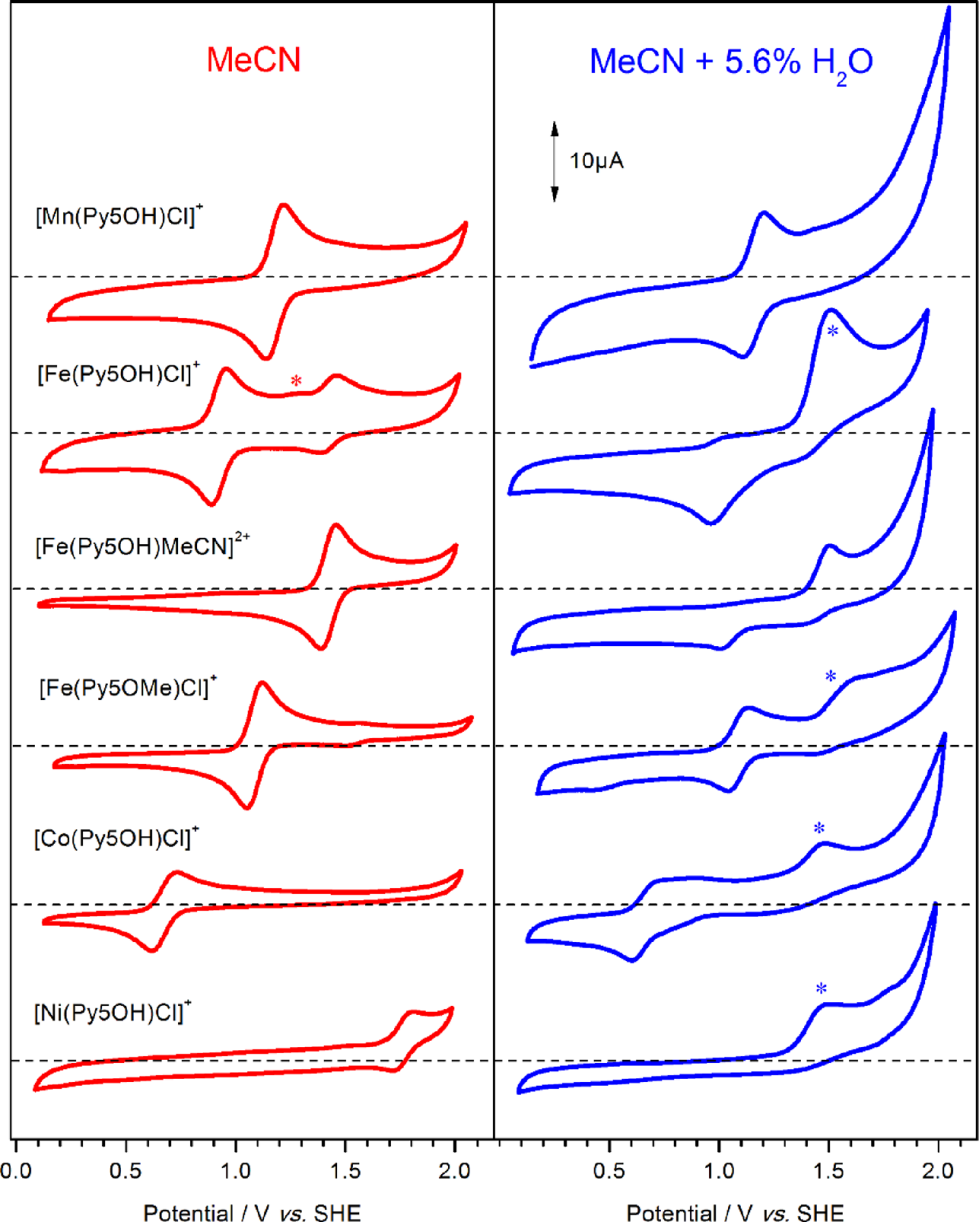
CVs of 0.5 mM [M(Py5**OH**)Cl]^+^ (M = Mn, Fe, Co, and Ni), [Fe(Py5**OH**)MeCN]^2+^, and [Fe(Py5**OMe**)Cl]^+^ in dry MeCN (red lines) and with 5.6% v/v water addition (blue lines) corrected with the dilution factor. All samples contain 0.1 M TBAPF_6_. The waves marked with asterisk are assigned to the chloride oxidation (Figure S11). Scan rate = 100 mV s^–1^.

As we reported previously, the CVs of [M(Py5**OH**)Cl]^+^ in dry MeCN show only one reversible one-electron redox couple, M^III^/M^II^, except for [Fe(Py5**OH**)Cl]^+^ (*vide infra*).^43^ For comparison, these data are shown on the left panel in Figure 3. The right panel of Figure 3 shows the CVs of the same complexes after the addition of 3 M H_2_O (∼6000 equiv with respect to the metal complex). Independent of the complexes, this led, especially at potentials above 1.6 V, to an enhancement of the charging current due to capacitive effects arising from the partial oxidation of the glassy carbon (GC) surface (Figure S9).^57^

#### 2.2.1 [Mn(Py5**OH**)Cl]^+^

**Figure 4:**
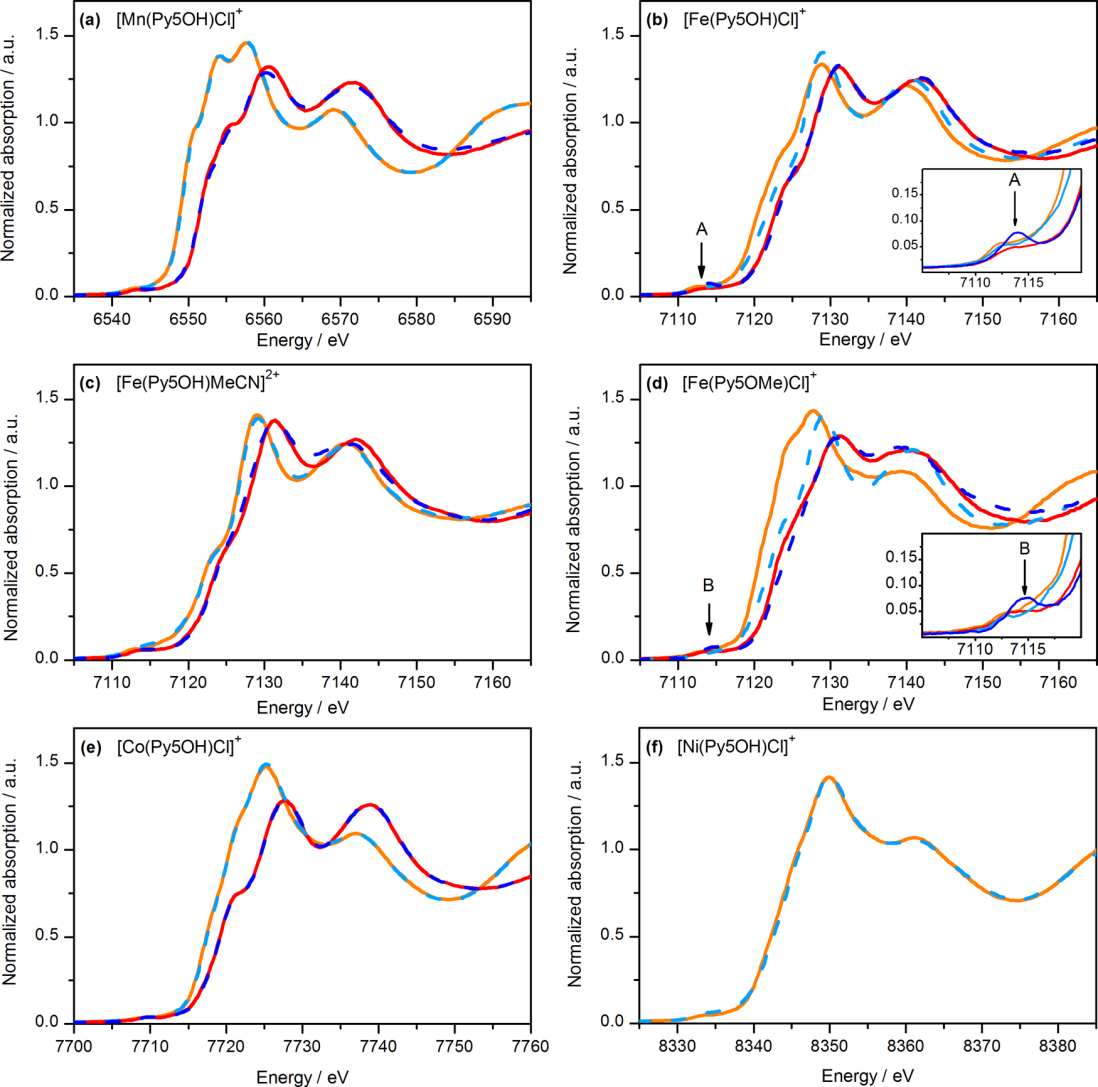
XANES spectra of the [M(Py5**OR**)X]^+^ complexes [(a) M = Mn, R = H; (b) M = Fe, R = H; (c) M = Fe, R = H; (d) M = Fe, R = Me; (e) M = Co, R = H; and (f) M = Ni, R = H] in dry MeCN (solid lines) and after the addition of 5.6% v/v water (dashed lines). The spectra were recorded before (orange and light blue lines) and after (red and dark blue lines) electrochemical oxidation. All samples contain 0.1 M TBAPF_6_. The insets show the pre-edge region in detail. The data for the oxidized Ni complex are not included because the oxidation was incomplete.

**Figure 5:**
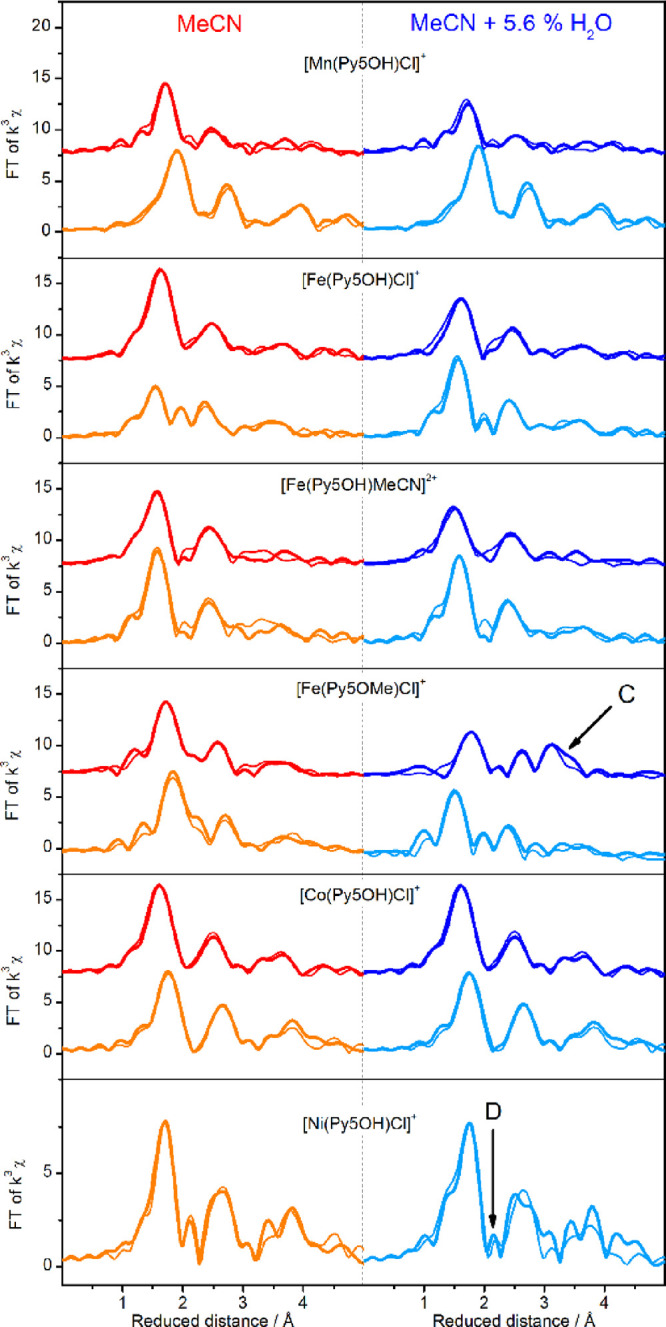
FT-EXAFS spectra (*k*^3^-weighted) of the [M(Py5**OR**)X]^+^ complexes in dry MeCN (left) and in MeCN containing 5.6% v/v water (right). The spectra were recorded for the reduced (orange and light blue lines) and one-electron oxidized (red and dark blue lines) forms of the complexes. All samples contain 0.1 M TBAPF_6_. The spectra were recorded at 20 K and are offset for clarity. Simulations of the experimental data are shown as thin lines, and the parameters are given in Table S5. EXAFS spectra in k-space are shown in Figure S10.

The data in Figure 3 show that the [Mn(Py5**OH**)Cl]^+^ complex was not affected by water addition as it shows an unchanged redox wave for the Mn^III^/Mn^II^ couple at 1.20 V. Importantly, no Mn^IV^/Mn^III^ oxidation or catalytic wave was observed. The stability of this complex was further supported by X-ray absorption spectroscopy (XAS). Both the X-ray absorption near-edge structure (XANES; Figure 4) and the extended X-ray absorption fine structure (EXAFS; Figure 5 and Figure S10) data for the reduced complex were essentially identical in dry MeCN and 3 M water, and the same was also the case for the oxidized complex. The edge positions and Mn–N distances were typical for Mn^II^ in case of the reduced form and Mn^III^ in the oxidized species.

Thus, while the [Mn(Py5**OH**)Cl]^+^ complex appears to be stable in 3 M water, it is unable to support water coordination and water oxidation due to its inability to exchange the apical Cl^–^ ligand against the water-derived ligand and a concomitant too high potential for oxidation beyond Mn^III^.

#### 2.2.2 [Fe(Py5**OH**)Cl]^+^, [Fe(Py5**OH**)MeCN]^2+^, and [Fe(Py5**OMe**)Cl]^+^

The binding of Cl^–^ to Fe^II^ in [Fe(Py5**OH**)Cl]^+^ is weaker as compared to Mn^II^ in [Mn(Py5**OH**)Cl]^+^, as the cyclic voltammogram of [Fe(Py5**OH**)Cl]^+^ in dry MeCN shows a second wave at *E*_1/2_ = 1.45 V assigned to a partial substitution (about 30%) of the chloride ligand by a solvent molecule yielding [Fe(Py5**OH**)MeCN]^2+^ (Figure 3, left panel).^46^ In addition, a weak wave is seen that originates from the oxidation of Cl^–^ in MeCN (asterisk in Figure 3; see Figure S11 for details).^46^ The CV of [Fe(Py5**OMe**)Cl]^+^ in dry MeCN, which is highly similar to that originally reported by Stack’s group,^44^ also showed two redox waves, but the change from R = **OH** to R = **OMe** shifted the wave for the chloride-ligated complex by +160 mV (*E*_1/2_ = 1.11 V) and that for the MeCN-ligated complex by +100 mV (*E*_1/2_ = 1.55 V). The latter species is present in a small amount (ca. 4% of the total Faradaic current of the iron complex); i.e., the chloride is dissociating to a much lesser extent than observed for [Fe(Py5**OH**)Cl]^+^.^37,44^

DFT calculations corroborate these observations, indicating that the chloride exchange for MeCN is less favorable by 2.5 kcal mol^–1^ in the [Fe(Py5**OMe**)Cl]^+^ compared to the [Fe(Py5**OH**)Cl]^+^ complex (see Table 2). Similar to the lower stability of the [Fe(Py5**OMe**)Cl]^+^ complex in borate buffer discussed above, this difference between the sister complexes is due to the tilt of the axial pyridine ring that is induced by the methoxy groups and the associated poorer overlap of metal–ligand orbitals. This results in a 2.1 kcal mol^–1^ stabilization of the high-spin (quintet) over the low-spin (singlet) state (see Table S4), which in turn favors the high-spin (HS) [Fe(Py5**OMe**)Cl]^+^ complex over the low-spin (LS) [Fe(Py5**OMe**)MeCN]^2+^ complex.

**Table 2:** Exchange Energies of Axial Ligands in [M(Py5**OR**)X]^+^ Complexes Calculated with DFT(B3LYP*) Using the SMD Solvation Model with Different Solvents^a^

	ligand exchange energies							
	SMD MeCN		SMD H_2_O					
	Cl^–^ to MeCN		Cl^–^ to MeCN		Cl^–^ to H_2_O		Cl^–^ to OH^–^	
complex	M^II^	M^III^	M^II^	M^III^	M^II^	M^III^	M^II^	M^III^
[Mn-Py5**OH**-X]	5.32		4.14					
[Fe-Py5**OH**-X]	0.55	9.75	–1.15	6.91	6.69	9.32	10.53	–2.01
[Fe-Py5**OMe**-X]	3.06	8.88	1.79	6.26	11.05	10.40	11.99	–1.25
[Co-Py5**OH**-X]	2.79	7.13	1.54	4.28				
[Ni-Py5**OH**-X]	–0.16		–1.40					

a Exchange energies are calculated for the following relative concentrations: [M(Py5**OR**)Cl]^+^ = 1, [MeCN] = 3.83 × 10^4^, [H_2_O] = 6.66 × 10^3^, and [OH^–^] = 1.20 × 10^–4^. The energy of the free OH^–^ ligand has been adjusted by +12.7 kcal/mol so that p*K*_a_(water) = 14.0.

**Figure 6:**
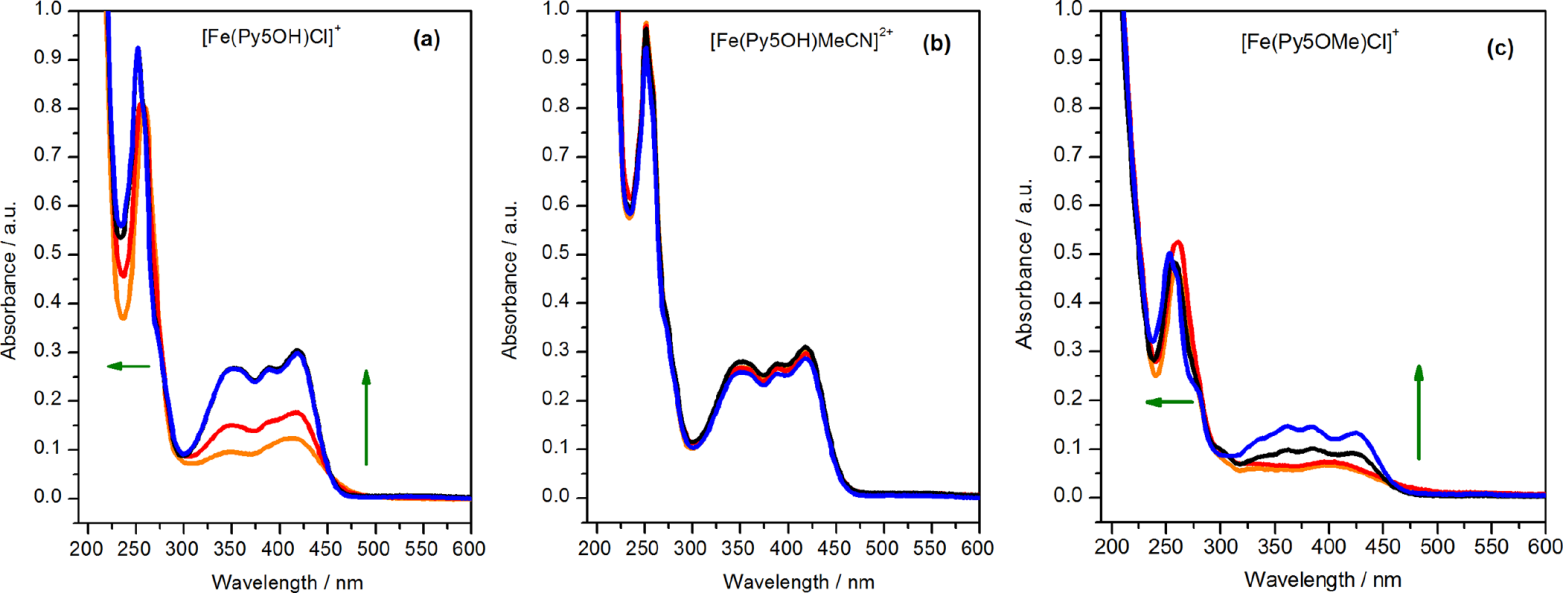
UV–vis spectra of (a) 50 μM [Fe(Py5**OH**)Cl]^+^, (b) 50 μM [Fe(Py5**OH**)MeCN]^2+^, and (c) 50 μM [Fe(Py5**OMe**)Cl]^+^ in dry MeCN without (orange line) or with (red line) the addition of 0.1 M TBAPF_6_ and after the addition of 5.6% v/v H_2_O (corrected with the dilution factor) to the above MeCN solutions without (black line) or with TBAPF_6_ (0.1 M, blue line). The green arrows indicate the transformation of [Fe(Py5**OR**)Cl]^+^ into [Fe(Py5**OR**)MeCN]^2+^.

The addition of water did not alter the anodic CV scan of the chloride-free iron complex [Fe(Py5**OH**)MeCN]^2+^ (Figure 3, right panel). The stability of this complex was further established by UV–vis spectroscopy, which showed that the spectra in dry MeCN and in 3 M water were identical (Figure 6b). Together, these data show that water/hydroxide binding is unfavorable in the reduced state, which is supported by the DFT calculations showing preferable MeCN over water binding (by 7.9 kcal mol^–1^, see Table 2). Similarly, the XANES and EXAFS spectra of [Fe(Py5**OH**)MeCN]^2+^ with and without water addition are fully consistent with this conclusion (Figures 4c and 5; Figure S10 and Table S5). In this regard, this complex in its divalent state is similar to tetrapyridine ferrous complexes [Fe(N4Py)X]^2+^ (X = solvent), favoring the coordination of MeCN as the sixth ligand with respect to H_2_O, but different from the tripyridine complex [Fe(Bn3Py)X_2_]^2+^ (Bn3Py = *N*-benzyl-1,1-di(pyridin-2-yl)-*N*-(pyridin-2-ylmethyl)methanamine), which exhibits rapid hydrolysis in the presence of H_2_O.^58^

By contrast, water addition modified significantly the cathodic CV scan of [Fe(Py5**OH**)MeCN]^3+^, which in the presence of water showed two reduction waves: one at *E*_p_ = 1.40 V, corresponding to the reduction of the MeCN-ligated complex, and a second wave at *E*_p_ = 1.05 V. Even if this second wave has a similar potential to the reduction of [Fe(Py5**OH**)Cl]^2+^, it must have a different origin since Cl^–^ is not present in these samples. Instead, the wave must come from a species with a water-derived ligand that is formed when the complex is oxidized. In the reduced state, this species then undergoes a ligand exchange back to the original [Fe(Py5**OH**)MeCN]^2+^ complex, explaining the absence of a corresponding oxidative wave. DFT calculations support the binding of OH^–^ to the Fe^III^ form (Table 2). Consistent with this interpretation, the FT EXAFS spectra of [Fe(Py5**OH**)MeCN]^3+^ show, after the addition of water, decreased peak amplitudes (Figure 5, blue line), which indicate the formation of a second species. The EXAFS simulation that is shown in Figure 5 includes a major fraction of the oxidized complex with MeCN (60%) plus a minor species (40%) that is simulated with a short Fe–O distance of around 1.9 Å, consistent with a hydroxo ligand (see also Table S5).

While a significant fraction of [Fe(Py5**OH**)MeCN]^2+^ undergoes water/hydroxide binding during oxidation to Fe^III^, still further oxidation, presumably an Fe^IV^=O species, cannot be observed in CVs up to 2 V.^59,60^ Noteworthily, the CV pattern of [Fe(Py5**OH**)MeCN]^2+^ has similar features to that of the tetrapyridine containing complex [Fe(N4Py)]^2+^ reported previously.^61^ But while, in the latter case, the two consecutive redox events observed in the MeCN/H_2_O mixture were attributed to Fe^III^/Fe^II^ and Fe^IV^=O/Fe^III^ couples, we argue that, in our case, both waves (Figure 3, right panel) originate from Fe^III^/Fe^II^ oxidation, with different solvents as the apical ligand.

For [Fe(Py5**OH**)Cl]^+^, the CV changed drastically upon water addition (Figure 3, right panel). The intensity of the original oxidative wave at *E*_1/2_ = 0.95 V arising from [Fe(Py5**OH**)Cl]^+^ significantly decreased. Instead, a new broad, quasi-reversible redox process is observed at *E*_p_ = 1.50 V. While this is the same potential as observed above for [Fe(Py5**OH**)MeCN]^2+^, this wave is broader and has about twice the amplitude. This indicates that the solvation of Cl^–^ by water facilitates a nearly complete Cl^**–**^/MeCN exchange and that the Cl^–^ oxidation occurs at nearly the same potential (asterisk in Figure 3; Figure S11) as that of [Fe(Py5**OH**)MeCN]^2+^. The essentially complete conversion of [Fe(Py5**OH**)Cl]^+^ to [Fe(Py5**OH**)MeCN]^2+^ in the presence of water with electrolyte was confirmed by the UV–vis spectra depicted in Figure 6a (compare to Figure 6b). It also explains the drastic changes in the XANES and EXAFS data upon water addition (Figures 4b and 5) because the Cl^–^/MeCN exchange is coupled to a HS/LS conversion. This interpretation was corroborated by DFT calculations (Table 2 and Table S4). Interestingly, a reductive wave was still observed at *E*_p_ = 0.95 V during the cathodic scan, while the reductive feature at 1.50 V remained smaller than expected. This indicates a daughter product formation during anodic oxidation at 1.50 V (Figure S12) that is in line with a higher binding affinity of Cl^–^ to Fe^III^ than to Fe^II^ (see Table 2).

The addition of water to [Fe(Py5**OH**)Cl]^2+^ results in only a small change in the XANES edge shape (Figure 4b, dark blue line), indicating that water has a smaller effect on Fe(III) than on Fe(II). However, a decrease in the amplitudes of the peaks in the Fourier transform of EXAFS (Figure 5, red and blue lines) was observed, and the pre-edge feature in the XANES was now more intense (see A in the inset in Figure 4b). This suggests that one or more additional species were present, with a noncentrosymmetric coordination, for example, species with a short Fe–O bond (Table S6).

In contrast to [Fe(Py5**OH**)Cl]^+^, for [Fe(Py5**OMe**)Cl]^+^, the main redox wave observed in dry MeCN was still present even after the addition of 3 M water, though at a diminished amplitude (Figure 3, right panel). Additionally, a new broad, quasi-reversible wave at *E*_p_ = 1.62 V was observed, which we interpret as a superposition of the oxidation of Cl^–^ and of the MeCN-coordinated complex. There are no clear indications in the CV for a reduction of species with a hydroxide-derived ligand for the [Fe(Py5**OMe**)OH]^2+^ complex; however, as shown above for the other two Fe complexes, these signals occur at about the same potential as the reduction of the Cl^–^-bound complex; thus, the formation of [Fe(Py5**OMe**)OH]^2+^ cannot be excluded. Again, the UV–vis spectra of [Fe(Py5**OMe**)Cl]^+^ follow the electrochemical observations by showing a comparatively small change after the addition of a supporting electrolyte and an incomplete dissociation of the chloride ligand in the presence of water (see Figure 6c).

The EXAFS spectrum of [Fe(Py5**OMe**)Cl]^2+^ is simulated well with only a Cl-ligated Fe(III) species (Figure 5 and Table S5). The addition of water to the oxidized sample increases the pre-edge intensity, indicating a deviation from the initial octahedral geometry (B in Figure 4d). Interestingly, the Fe–N peak is now at a significantly longer distance than in the reduced sample with 3 M H_2_O in the FT-EXAFS spectrum (Figure 5) and completely overlaps with the position where Cl^–^ is expected to be, which makes it impossible to say to what extent Cl^–^ is bound to Fe(III). Additionally, a new broad peak appears at a reduced distance of 3.1 Å (C in Figure 5). This peak is consistent with a 3.33 Å Fe–Fe distance, such as formed by a mono-μ-oxo bridge between iron ions. This indicates the formation of either a bi- or polynuclear species or of small clusters of Fe oxide/hydroxide. These occur due to the reaction with water, as intermediate(s) on the way to the final degradation product under oxidative conditions, the catalytically inactive bulk Fe oxide/hydroxide.

In conclusion, while water binding is observed to various degrees for all three Fe^III^ complexes, the formation of the Fe^IV^=O state could still not be observed under an applied potential up to 2 V. Instead, for [Fe(Py5**OMe**)Cl]^+^, indications were found for an oxo-bridged dimer formation, which may be the start of Fe-oxide formation. This is in line with the observed rapid degradation of this complex in borate buffer (*vide supra*).

#### 2.2.3 [Co(Py5**OH**)Cl]^+^

For [Co(Py5**OH**)Cl]^+^, the redox wave observed at *E*_1/2_ = 0.70 V in dry MeCN decreases in intensity upon water addition with a concomitant formation of a reversible redox feature at *E*_1/2_ = 0.86 V (Figure 3). We assign this signal to the formation of [Co(Py5**OH**)MeCN]^2+^. This is supported by DFT calculations that find that the redox potential of [Co(Py5**OH**)MeCN]^2+^ is 190 mV above that of [Co(Py5**OH**)Cl]^+^ (*E*_1/2_ = 0.77 and 0.59 V, respectively).

For [Co(Py5**OH**)Cl]^+^, the EXAFS data do not provide evidence for Cl^–^/MeCN exchange (Figure 5). This discrepancy to the electrochemistry data may be explained by the incomplete loss of Cl^–^ and the fact that this ligand change is not associated with a spin transition and accompanying structural changes, as seen above for the Fe complexes. Nevertheless, some difference would be expected in the EXAFS since Cl^–^ is a much stronger back scatterer than O and N. The addition of water does not affect the XAS spectra of [Co(Py5**OH**)Cl]^2+^ (Figures 4e and 5; Table S5). Thus, no evidence for water or hydroxide binding to Co^III^ was obtained. This may explain why, even with the Co complex, which has the lowest *E*_1/2_ for the M^II/III^ oxidation of the complexes studied here, no water oxidation activity was observed.

#### 2.2.4 [Ni(Py5**OH**)Cl]^+^

For the [Ni(Py5**OH**)Cl]^+^ complex, in the presence of 3 M water, a new anodic peak at *E*_p_ = 1.50 V was observed in addition to a strongly diminished Ni^II/III^ redox couple at *E*_1/2_ = 1.80 V, indicating the release of Cl^–^ in a significant fraction of metal centers upon water addition (Figure 3). Here, no additional species was detected, possibly because its potential was outside of the range of the CV scan or was coinciding with the above waves.

In the FT EXAFS, the chloride shell peak of [Ni(Py5**OH**)Cl]^+^ is strongly reduced after the addition of 3 M water (D in Figure 5) and approaches zero in EXAFS fits when considering N from MeCN as the apical ligand (Table S5). A similar behavior has been observed previously for the similar [Ni(Py5**Me**)Cl]^+^ complex^51^ and is in line with our DFT calculations that indicate that MeCN is preferred over the Cl^–^ binding in the presence of 3 M water (Table 2).

The Ni complex has the highest redox potential for the M^II/III^ oxidation in this metal series. It was not possible to generate its fully oxidized form during XAS sample preparation with our electrochemical flow cell, and thus, no additional data on the Ni^III^ complex could be obtained.

## 3 Conclusions

We investigated water oxidation by the base metal complexes [M(Py5**OH**)Cl]^+^ (M = Mn, Fe, Co, Ni) and the reference complex [Fe(Py5**OMe**)Cl]^+^ in borate buffered pH 8.0 solutions. Only one complex, [Fe(Py5**OMe**)Cl]^+^, showed significant water oxidation activity in the buffered ruthenium dye photo-oxidant system, but further investigation with XAS revealed a rapid degradation of [Fe(Py5**OMe**)Cl]^+^ into FeOOH species. This observation was further supported by UV–vis spectroscopy and electrochemistry. A similar process was observed for [Fe(Py5**OH**)Cl]^+^, however, at a significantly slower rate. We thus conclude that the lower stability of the methylated complex in borate buffer makes it an efficient precursor for an amorphous Fe-oxo/hydroxo species that is active in water oxidation catalysis. In this study, we could not reproduce the previously reported water oxidation activity for [Co(Py5**OH**)Cl]^+^ and [Fe(Py5**OH**)Cl]^+^.^52,53^ We propose that, in the earlier experiments, the reported activities could have come from cobalt oxides and iron oxide/hydroxides that were not observed with DLS (see Table S3).^32−36,62^

To understand the lack of water oxidation activity, we performed detailed investigations into their redox and ligand exchange properties in MeCN and MeCN/H_2_O solutions. All the complexes, except [Mn(Py5**OH**)Cl]^+^, exchange the apical chloride ligand by MeCN in the presence of 3 M water in MeCN. Only for the iron complexes we obtained evidence for partial hydroxide binding in the Fe^III^ state. None of the complexes supported metal oxidation beyond the M^III^ oxidation state at potentials up to 2.0 V. Thus, the major bottlenecks for all complexes of this study were that they could perform only a one-electron oxidation and that the affinity for substrate water binding was low. Together, this prevented the formation of the catalytic key intermediates, i.e., of M^IV^=O or M^III^–O^•^, which would allow O_2_ formation via I2M, WNA, or radical coupling mechanisms.

Given the precedence in mononuclear Ru WOCs,^11,12^ in the majority of high-valent Mn and Fe complexes,^63^ and in the Mn_4_CaO_5_ cluster in photosystem II,^5^ we expect that stronger σ-donor ligand systems may be better suited for supporting the required higher oxidation states. This can be achieved by incorporating one or more negatively charged donor atoms to the ligand, which may be viewed as an alternative to the carboxylate ligands and μ-oxo bridges of the Mn_4_CaO_5_ cluster.^19^ Notable examples of strong σ-donors employed to stabilize M^IV^ and even M^V^ species are carboxamido,^64^ hydrazide,^65^ phenolate,^66^ and carboxylate^67^ groups. Importantly, the candidate ligand system would also need to support the binding of one substrate water molecule, as well as stepwise proton release from it, for example, via providing a hydrogen bonding partner.^68^ Finally, if an I2M mechanism is targeted, then the formed M^IV^=O unit must not be obstructed by bulky ligands.

For the Fe complexes, one additional problem in this study has been their low stability in water that led to the degradation to metal oxides or hydroxides. To avoid this, the overall binding strength of the metal in the ligand system must be high enough. Here we show that even rather peripheral ligand changes (R in Scheme 1) can have a significant effect on the overall stability in water and thus may provide opportunities for tuning the binding strength. In addition to a suitable ligand design, degradation may be also prevented by placing the molecular catalysts in environments that limit the access of water to that required for efficient catalysis. In photosystem II, the Mn_4_CaO_5_ cluster is situated within a large protein complex in which the access of water is regulated by three channels. In addition, the water molecules near the Mn_4_CaO_5_ cluster are arranged mainly along one face of the cluster, and they are highly ordered due to H-bonding networks that are supported by specific amino acids.^69−72^ In case of molecular WOCs, the bulk water access may be limited by water/inert solvent mixtures (e.g., 3 M water in MeCN) or, more specifically, by embedding the catalyst in matrices such as MOFs, redox active polymers, or designed polypeptides/proteins.^73^

This study aligns with previous reports^33−35^ in showing the complexity of developing and testing molecular water oxidation catalysts comprising first-row transition metals. Importantly, it also demonstrates that, by detailed experiments and analysis, the bottlenecks can be identified and rational strategies for the next generation of complexes can be developed. With insights from such studies, it thus seems feasible that similar improvements as seen previously for Ru-based water oxidation catalysts^11,12^ will be achievable.

## 4 Experimental Methods

All starting reagents were obtained from commercial sources and used as received. All glassware was cleaned and dried overnight at 120 °C. The synthesis of the metal complexes was conducted under a dry argon atmosphere. The synthesized samples were stored in air without any observed degradation.

The M^II^ complexes were fully characterized using ^1^H NMR, FT-IR, UV–vis, HR-MS, and CHN elemental analysis and compared with our previous study to confirm the identity of the products.^46^ The solutions of M^III^ complexes were obtained by electrolysis in MeCN with TBAPF_6_ as the electrolyte (0.1 M) as described below.

### 4.1 Synthesis of [M(Py5**OH**)Cl]PF_6_ and [Fe(Py5**OH**)MeCN](ClO_4_)_2_

Caution: Perchlorate salts are potentially explosive and should be handled with care.

The syntheses of the Py5**OH** ligand and the metal complexes [M(Py5**OH**)Cl]PF_6_ (M = Mn, Fe, Co and Ni) and [Fe(Py5**OH**)MeCN](ClO_4_)_2_ were carried out according to the previously reported procedure with minor modifications.^46^ Specifically, to ensure the high purity of the metal compounds for their quantitative experiment application, the addition of KPF_6_ as a source of counter ion was carried out with exact stoichiometry. The washing procedure of the obtained metal complexes was done with a large amount of cold dry MeOH. Only for the two iron complexes was it possible to collect enough material by recrystallization. This was carried out by dissolving the compound in MeCN and slow pervaporation of diethyl ether over a week.

Elemental analysis for the compounds:

**[Mn(Py5OH)Cl]PF_6_**. MnC_27_H_21_N_5_ClO_2_PF_6_ (682.85 g mol^–1^) calculated %: C 47.49, H 3.10, N 10.26 found %: C 47.78, H 3.28, N 10.08.
**[Fe(Py5OH)Cl]PF_6._** FeC_27_H_21_N_5_ClO_2_PF_6_ (683.75 g mol^–1^) calculated %: C 47.43, H 3.10, N 10.24; found %: C 47.61, H 3.26, N 10.09.
**[Fe(Py5OH)MeCN](ClO_4_)_2_**. FeC_29_H_24_N_6_Cl_2_O_10_ (745.28 g mol^–1^) calculated %: C 46.86, H 3.25, N 11.31; found %: C 46.30, H 4.30, N 10.98.
**[Co(Py5OH)Cl]PF_6_**. CoC_27_H_21_N_5_ClO_2_PF_6_ (686.84 g mol^–1^) calculated %: C 47.21, H 3.08, N 10.20; found %: C 48.06, H 4.29, N 9.15.
**[Ni(Py5OH)Cl]PF_6_**. NiC_27_H_21_N_5_ClO_2_PF_6_·4H_2_O (686.60 g mol^–1^) calculated %: C 47.23, H 3.08, N 10.20; found %: C 48.22, H 4.26, N 9.03.

### 4.2 Synthesis of Py5**OMe**

Py5**OH** (200 mg, 0.447 mmol) was dissolved in 20 mL of dry THF to produce an orange solution, and 5 equiv of NaH (54 mg, 2.236 mmol) was added to the solution. Immediately, the peach mixture started bubbling, and methyl iodide (318 mg, 2.236 mmol) was added slowly to the reaction mixture at room temperature and finally heated to 40 °C overnight. After that, the solution was acidified with 5% HCl to a pH of 4.0 to dissolve the product in the aqueous layer and then basified with saturated aqueous Na_2_CO_3_ to pH 9.0 with the precipitation of a white solid. The product was extracted with CHCl_3_ (3 × 30 mL), and the organic phases were combined and dried over Na_2_SO_4_. Removal of the solvent under reduced pressure followed by recrystallization from cold acetone/diethyl ether afforded Py5**OMe** as a white solid (85 mg, 0.179 mmol, yield: 40%). ^1^H NMR (400 MHz, CDCl_3_): 3.20 ppm (6 H, s, C-OMe), 7.18 ppm (4 H, td, J = 3.0 Hz, and 1.3 Hz, 5-Hpy-a), 7.45 ppm (4 H, d, J = 7.6 Hz, 3-Hpy-a), 7.59 ppm (2 H, d, J 4.6 Hz, 3-Hpy-b), 7.57 ppm (4 H, t, J = 5.0 Hz, 4-Hpy-a), 7.74 ppm (1 H, t, J = 7.8 Hz, 4-Hpy-b), 8.56 ppm (4 H, d, J = 4.4 Hz, 6-Hpy-a). HR MS: *m/z* [**L** + H]^+^ 476.5502 (calc. 476.2087). Solid FT-IR (KBr) of the Py5**OMe** differentiates from the reported Py5**OH** by the absence of the strong *v*(O–H) stretching at 3270 cm^–1^.^46^ Instead, a new band appears in the 2800–3000 cm^–1^ region assigned to the *v*(C–H) stretching of the two methoxy groups.

### 4.3 Synthesis of [Fe(Py5**OMe**)Cl]PF_6_

Py5**OMe** (38.1 mg, 0.08 mmol) was dissolved in 15 mL of MeOH in a 100 mL bottom flask. FeCl_2_·4H_2_O (15.9 mg, 0.08 mmol) was dissolved in 6 mL of methanol and added dropwise to the ligand. The solution changed from transparent to yellow. After 30 min, KPF_6_ (16.5 mg, 0.09 mmol, dissolved in 6 mL of MeOH) was added slowly, and the solution was kept under constant stirring at RT until the day after. A few drops of diethyl ether were necessary to promote the precipitation of the product that was collected by Buchner filtration. The metal complex was washed with ice-cold dry methanol. The collected filtrate was redissolved in 10 mL of dry MeCN. The open vial was stored in a closed desiccator with ethyl acetate. After a week, the formed yellow crystals were collected and dried under a vacuum for 48 h (26.8 mg, 0.038 mmol, yield: 47%). Solid FT-IR (KBr) of the complex shows the same vibration modes as the ligand with a blueshift of 12 cm^–1^ and the characteristic P–F stretching at 842 cm^–1^ from the PF_6_^–^ ion. FeC_29_H_25_N_5_ClO_2_PF_6_ (711.81 g mol^–1^) calculated %: C 48.93, H 3.54, N 9.84; found %: C 48.83, H 3.75, N 10.17. The UV–vis spectrum shows a strong sharp peak at 256 nm (ε = 18.04 × 10^3^ M^–1^ cm^–1^) and a broad absorption at 300–500 nm with two main maxima at 404 (ε = 2.54 × 10^3^ M^–1^ cm^–1^) and 335 nm (ε = 2.40 × 10^3^ M^–1^ cm^–1^).

### 4.4 Cyclic Voltammetry

CVs in MeCN with the addition of water were conducted in the following way. The electrolyte solution consisted of 0.1 M TBAPF_6_ in dry MeCN. The reference electrode was made by an Ag wire coated with AgCl and sealed in a porous glass tipped tube that was refilled with the same electrolyte solution. The electrochemical stability of the Ag pseudo reference electrode was tested over 2 days by recording the cyclic voltammetry of ferrocene. A drift of 0.014 V was observed in the redox potential (*E*_1/2_ (Fc^+^/Fc) = 0.47 V *vs* Ag pseudo reference in MeCN). Deionized water was added to the electrolyte solution to 5.6% v/v. All the reported CVs are corrected by the dilution factor. At the end of each experiment, the potential was calibrated with ferrocene as the reference (*E*° (Fc^+^/Fc) = +0.624 V *vs* SHE).

CVs in aqueous solution were done with a 9:1 mixture of borate buffer (100 mM, pH 8.0) and dry MeCN for the blank or with a 5 mM metal complex MeCN solution. The reference electrode was a Ag/AgCl 3 M KCl that was regularly checked using K_4_[Fe(CN)_6_] as the standard (*E*° (Ag/AgCl) = +0.210 V *vs* SHE).

For all the CVs, a glassy carbon working electrode (3 mm diameter) was used that was polished with alumina particles of 1 and 0.05 μm immediately prior to use. The counter electrode was a platinum rod polished with sandpaper before use. Unless stated otherwise, the following parameters were used to record CVs: scan rate, 100 mV s^–1^; step potential, 0.002 V. All potentials given in this study are relative to the SHE electrode.

### 4.5 X-ray Absorption Spectroscopy

X-ray absorption spectroscopy was performed at the KMC-3 beamline at the BESSY II synchrotron, Berlin, Germany. The storage ring was operated in the top-up mode (300 mA). The incident X-ray energy was scanned through the Mn, Fe, Co, and Ni K-edge regions using a silicon (111) double-crystal monochromator. Measurements were performed with samples positioned at 45° with respect to the incident beam in a helium-cooled cryostat (Oxford Instruments) at 20 K. The solution samples of the metal complexes (1 mM) in MeCN with 0.1 M TBAPF_6_ were oxidized with a custom-made continuous flow electrosynthesis cell.^74^ The applied potential was selected by recording a slow (10 mV s^–1^) cyclic voltammogram in a steady condition. The extent of electrolysis was monitored by recording the current response (typically around 0.2 mA at 0.05 mL min^–1^ flow rate) and changes in the UV–vis spectrum to provide an estimation of the percentage of the conversion. For the complexes with 5.6% of water, a 11.2% v/v water–electrolyte solution was added to the outlet of the flow cell, resulting in a halved dilution (0.5 mM). The oxidized sample solution was collected and frozen immediately in liquid nitrogen. Kα fluorescence signals from the samples were recorded with a 13-element silicon drift detector (RaySpec) positioned perpendicular to the incident beam. For each sample, 8–16 scans were taken. Each scan was collected on a new sample spot to avoid possible radiation damage; additionally, three consecutive scans at the same sample spot confirmed that there was no observable radiation damage on the time scale of the XAS measurement. A 10 μm Fe, Co, or Ni foil (Goodfellow Cambridge Limited) positioned behind the sample served as an energy calibration standard. For Mn, a thin layer of KMnO_4_ was used. Energy calibration was done by assigning the position of the first inflection point of the absorption of the Fe, Co, and Ni foils to energies 7112, 7709, and 8333 eV, respectively. The position of the KMnO_4_ pre-edge was set to 6543.3 eV. *E*_0_ values used for EXAFS extraction were 6539 (Mn), 7115 (Fe), 7710 (Co), and 8334 eV (Ni). EXAFS simulations were done with the FEFF 9.0 software (using settings NLEG 6, CRITERIA 12 5, RPATH 7, SCF 7 1 30 0.05).^75^ The amplitude reduction factor *S*_0_^2^ was 0.9 for Mn, Co, and Ni and 0.85 for Fe. Least-squares fitting of *k*^3^-weighted EXAFS data was done with an in-house software (SimXLite). The fitting range was between *k* = 1.6 and 13 Å^–1^. The fitting included changing of interatomic distances for the first four single-scattering shells and Debye–Waller factors for all shells (with all multiple-scattering shells having the same Debye–Waller factor). Fit parameter errors were determined as described previously.^76^ A detailed list of parameters is presented in Table S4.

### 4.6 Light-Driven Oxygen Evolution Experiments

Oxygen evolution was measured by time-resolved membrane-inlet mass spectrometry (TR-MIMS). The total amount of oxygen was quantified from the peak heights that were calibrated for each day of measurement by injecting air-saturated water into the reaction chamber. Light-driven oxygen evolution was conducted by using [Ru(bpy)_3_](ClO_4_)_2_ as the photosensitizer (0.5 mM) and Na_2_S_2_O_8_ as the electron acceptor (2.5 mM), while the final concentration of the complexes was 10 μM for [M(Py5**OH**)Cl]^+^ and 2.50, 1.25, 0.63, and 0.31 μM for [Fe(Py5**OMe**)Cl]^+^ in borate buffer, pH 8.0 (40 mM). MeCN (0.2% v/v) was used to dissolve the complexes. A typical experiment was conducted under dimmed red room illumination to prevent the photo-activation of the photosensitizer before the start of the experiment. One milliliter of a reaction mixture was inserted into the reaction chamber that was kept at 22 °C by a thermostat. The light was provided by a custom-built blue LED device that surrounded the entire reaction chamber (24.8 mW at 470 nm). The solution was separated from the vacuum of the instrument by a semipermeable silicon membrane that allows only gasses to diffuse through. The reaction mixture was kept under constant stirring to allow degasification (required for a stable baseline) for 5 min. When the level of oxygen was sufficiently low and stable, the cell was illuminated, and the evolved oxygen was directly detected by the mass spectrometer *via* the membrane inlet. Blank experiments were conducted using the solution containing [Ru(bpy)_3_](ClO_4_)_2_ and Na_2_S_2_O_8_ in the buffer but without [M(Py5**OR**)Cl]^+^ complexes. After each experiment, the reaction chamber was washed with deionized water and a 0.01 M HCl solution. The blank experiment performed in the absence of [M(Py5**OR**)Cl]^+^ produces background oxygen originating from the instability of photochemically generated [Ru(bpy)_3_]^3+^.^77,78^ Chemical water oxidation was performed by adding an aqueous solution of [Ru(bpy)_3_]^3+^ (0.6 mM) to the [M(Py5**OR**)Cl]^+^ solution (10 μM) in borate buffer (0.04 M, pH 8.0) containing MeCN (0.2% v/v). To conduct the experiment in a reproducible fashion, fresh solutions of all the reactants were made every day. TONs were calculated by subtracting the oxygen contribution from the blank experiment. Errors were calculated with error propagation methods.

### 4.7 Dynamic Light Scattering (DLS)

Dynamic light scattering (DLS) measurements were performed with a Zetasizer Nano ZS from Malvern, Ltd. (Malvern, UK). Data were collected at 298 K. The instrument was tuned for Fe_2_O_3_ detection with a reflex index of 3.321. All the buffers were treated with a 20 μm Teflon filter before use, resulting in an attenuator factor of 11 that was taken as the absence of detectable nanoparticles.

### 4.8 Controlled Potential Electrolysis for O_2_ Catalytic Activity

O_2_ activity measurements with controlled potential electrolysis were done in a 2 mL cell equipped with a Clark-type oxygen sensor. The O_2_ signal was calibrated with a two-point calibration: air-saturated deionized water at 22 °C (273 μM) and oxygen-free water conditions. The electrolysis was carried out with customized electrodes to adapt in the 10 mm diameter cylindrical vessel of the Clark electrode. The glassy carbon working electrode had a shape of a square plate (5.0 × 5.5 × 1 mm) with a total surface area of 71 mm^2^. Before CPE, the electrode was carefully polished with a water suspension of alumina particles (1.0 μm). The customized reference electrode consisted of a silver wire coated with AgCl inserted in a glass capillary (1 mm ⌀ × 4 mm) containing a 3 M solution of KCl. A cylindrical molecular sieve was used as a frit by sealing it into one of the ends of the capillary. The stability of the reference electrode was regularly checked by measuring the half-peak potential of the redox couple Fe^II/III^ of K_4_[Fe(CN)_6_] before and after CPE. A maximum relative deviation of 3% of the potential was measured over the CPE experiment. The counter electrode consisted of a 10 mm glass tube that was separated from the test solution by a porous support. The solution was inserted into the counter electrode tube to prevent possible dilution processes during electrolysis. A glassy carbon rod was placed on the upper end of the tube. In a typical experiment, MeCN was used to prepare a 5 mM solution of [Fe(Py5**OMe**)Cl]^+^. A 10-fold dilution with borate buffer (100 mM, pH 8.0) was used for the CPE experiment. The same electrolyte composition was used for the assay with FeSO_4_. The working and reference electrodes were inserted into the cell that was filled with 1.6 mL of the solution under study. The counter electrode compartment was placed on the top and also used as a plunger to insulate the solution from the atmosphere. An air-saturated baseline was recorded for 1 min before applying a 2.0 V potential for 20 min. The oxygen recording was stopped after 25 min. Between experiments, the cell was carefully washed with 0.01 M HCl and deionized water. For the rinse test, the working electrode was washed with water and MeCN. We were particularly prudent to not scrape out possible depositions on the surface.

### 4.9 DFT Calculations

DFT calculations were performed using Gaussian09 E.01.^79^ The initial geometry optimization for all complexes was done using the B3LYP functional with the Lanl2DZ basis set for transition metals and 6-31G(p,d) for all other atoms. Thermal contributions were calculated from the subsequent Hessian calculations. Structures were then reoptimized using the B3LYP-D3 functional with the larger Lanl2TZ(f) basis set for the transition metals and 6-311+G(2df,2pd) for all other atoms. All optimizations were performed in the MeCN solvent using the default Polarizable Continuum Model (PCM) in Gaussian09. Final energies were calculated with the B3LYP* functional^80^ using the D3 parameters from B3LYP.^81^ This functional was previously shown to give good results for spin-state energetics in the spin-crossover complex [Fe(Py5**OH**)Cl]^+^.^47^ Final energy calculations were done using the Solvation Model based on Density (SMD).^82^ Binding energies were calculated from the reaction [M(Py5**OR**)MeCN]^*n*+^ + X ⇌ [M(Py5**OR**)X]^*n*+^ with all components in the MeCN solvent, including the apical ligand X. For reactions with differences in concentration between components, a configurational entropy factor has been added according to Boltzmann’s formula, *S* = *k*_B_ln *W*, where *k*_B_ is Boltzmann’s constant and *W* is the number of solvent molecules per complex.

Calculated redox potentials were obtained using the reaction [M(Py5**OR**)X]^*n*+^ ⇌ [M(Py5**OR**)X]^(*n* + 1)+^ + e^–^. The energy of the solvated electron was calculated using 4.28 V for the absolute potential of SHE. The choice of reference value affects the absolute potentials but not the comparison between complexes. Pure water was used in the SMD solvent model to calculate free energies of protonation reactions of water-derived ligands. A value of −264.0 kcal mol^–1^ was used for the absolute solvation energy of a proton in an aqueous solution,^83^ with −6.3 kcal mol^–1^ as a correction for the free energy of a gas phase proton from the Sackur–Tetrode equation.^84^ Reaction energies were calculated for pH = 7, and the correction for the reduced concentration of proton was −9.5 kcal mol^–1^ using pH*(−1.36) kcal mol^–1^. This gave −279.8 kcal mol^–1^ as the solvated Gibbs free energy of a proton in water at pH = 7.

## Supporting Information Available

Experimental details (synthesis of Ru bipyridyl complexes and XPS), supplementary figures, and atom coordinates of the optimized structures (PDF)
