# Supplementary material for: Water Oxidation by Pentapyridyl Base Metal Complexes? A Case Study
Source: Inorg Chem. 2022 Jun 6;61(24):9104–18. doi: 10.1021/acs.inorgchem.2c00631 (PMC9214691; doi:10.1021/acs.inorgchem.2c00631)
Supplement: Supplementary file 1 — ic2c00631_si_001.pdf [file ic2c00631_si_001.pdf]

Electronic Supplementary Information

Water Oxidation by Pentapyridyl Base Metal Complexes? A case study

Manuel Boniolo,[a] Md Kamal Hossain,[b] Petko Chernev,[a] Nina F. Suremann,[b] Philipp A. Heizmann,[b] Amanda S. L. Lyvik,[a] Paul Beyer,[c] Michael Haumann,[c] Ping Huang,[a] Nessima Salhi,[a] Mun Hon Cheah,[a] Sergii I. Shylin,[a,*] Marcus Lundberg,[a,*] Anders Thapper,[b,*] and Johannes Messinger [a,d,*]

[a] Molecular Biomimetics, Department of Chemistry – Ångström Laboratory, Uppsala University, 75120 Uppsala, Sweden

[b] Synthetic Molecular Chemistry, Department of Chemistry – Ångström Laboratory, Uppsala University, 75120 Uppsala, Sweden

[c] Physics Department, Freie Universität Berlin, 14195 Berlin, Germany

[d] Department of Chemistry, Chemical Biological Centre, Umeå University, 90187 Umeå, Sweden

* corresponding authors:

SIS (sergii.shylin@kemi.uu.se)

ML (marcus.lundberg@kemi.uu.se)

AT (anders.thapper@kemi.uu.se)

JM (johannes.messinger@kemi.uu.se)

Table of contents

Methods	3
Figure S1. Chemical water oxidation	4
Figure S2. Electrochemical water oxidation	5
Figure S3. Electrochemical water oxidation: blank experiment	6
Figure S4. XPS of electrodes	7
Figure S5. Electrolysis using rinsed electrodes	8
Figure S6. UV-Vis spectra of $[Fe(Py5OR)Cl]^+$	9
Figure S7. EXAFS spectra of $[Fe(Py5OMe)Cl]^+$	10
Figure S8. Molecular structures of $[Fe(Py5OR)Cl]^+$	11
Figure S9. CVs of the ligand	12
Figure S10. EXAFS of $[M(Py5OR)S]^+$ complexes	13
Figure S11. CVs of TBACl	14
Figure S12. CVs of $[Fe(Py5OH)Cl]^+$	15
Table S1. XPS: element composition	16
Table S2. XPS: binding energy	16
Table S3. DLS measurements	16
Table S4. DFT calculations parameters	17
Table S5. EXAFS fitting parameters	18
Table S6. Species detected in XAS experiments	20
Table S7. Half-potentials calculated using DFT	20
Coordinates of the DFT optimized geometries	21

Methods

Synthesis of $[Ru(bpy)_3](ClO_4)_2$

Caution: Perchlorate salts are potentially explosive and should be handled with care. The commercial $[Ru(bpy)_3]Cl_2{\cdot}6H_2O$ (202 mg, 0.270 mmol) was dissolved in water (2 mL). An aqueous solution of 5M $NaClO_4{\cdot}H_2O$ (1.75 mL) cooled in an ice bath was added dropwise to the cold $[Ru(bpy)_3]Cl_2{\cdot}6H_2O$ solution followed by the formation of an orange precipitate. The solid was filtered and washed with ice-cold water (2 mL). The collected product was then recrystallized from hot ethanol and the crystals were dried under vacuum overnight (127 mg, 0.164 mmol, yield: 61 %). MS-ESI: m/z: 285.1 $[Ru(bpy)_3]^{2+}$, 669.0 $[Ru(bpy)_3{\cdot}(ClO_4)^{-}]^{+}$.

Synthesis of $[Ru(bpy)_3](ClO_4)_3$

Caution: Perchlorate salts are potentially explosive and should be handled with care. The synthesis is a modified version of a previously reported procedure.[1] PbO_2 (500 mg, 2.1 mmol) was added to an orange saturated solution of $[Ru(bpy)_3]Cl_2{\cdot}6\ H_2O$ (350 mg, 0.490 mmol) in 3.5 M H_2SO_4 (18 mL) and the color changed to green. The reaction mixture was stirred for 10 min and filtered to remove the oxidizing agent. The green solution was then cooled in a water-ice bath and a cold solution of $NaClO_4$ (5 M) was added dropwise until precipitate stopped forming. The solid was collected by Buchner filtration and washed with ice-cold water. The product was dried in an oven at 90 °C for 45 minutes and kept overnight under vacuum (234.5 mg, 0.270 mmol, yield: 55.1 %).

XPS

X-ray photoelectron spectroscopy (XPS) was performed on a PHI *Quantera* II spectrometer using a monochromatic Al Kα source (hν = 1486.6 eV) operated at 50 W and 15 kV with a beam diameter of 200 μm. Survey spectra were collected while scanning a range from 1100 eV to 0 eV at a pass energy of 224 eV, a step size of 0.80 eV/step and 50 ms per step. High resolution spectra were collected with a pass energy of 55 eV and a step size of 0.10 eV/step. All spectra were analyzed using CasaXPS software,[2] employing the R.S.F. values from Multipak, the Ulvac-PHI software.[3] For the quantification a Shirley background correction was applied. All spectra were calibrated by setting adventitious carbon to 284.8 eV. To compare the results of the degradation experiments to the pure complex, $[Fe(Py5\mathbf{OMe})Cl]^{+}$ was dissolved in MeCN and drop-casted on a glassy carbon surface. The drop-casted iron oxide was prepared by dissolving $FeSO_4$ in borate buffer (pH 8.0, 90 mM) with 10 % v/v MeCN. Before measuring XPS, all samples were dried under vacuum for at least 24 h.

1. Shafirovich, V. Y.; Khannanov, N. K.; Shilov, A. E. Inorganic Models of Photosystem II of Plant Photosynthesis. Catalytic and Photocatalytic Oxidation of Water with Participation of Manganese Compounds. *J. Inorg. Biochem.* **1981,** *15* (2), 113-129.
2. Fairley, N. *CasaXPS*, 2.3.24; Casa Software Ltd: Teignmouth, UK, 1999.
3. *Multipak*, 9.8.0.19; Ulvac-phi, Inc.: 2017.

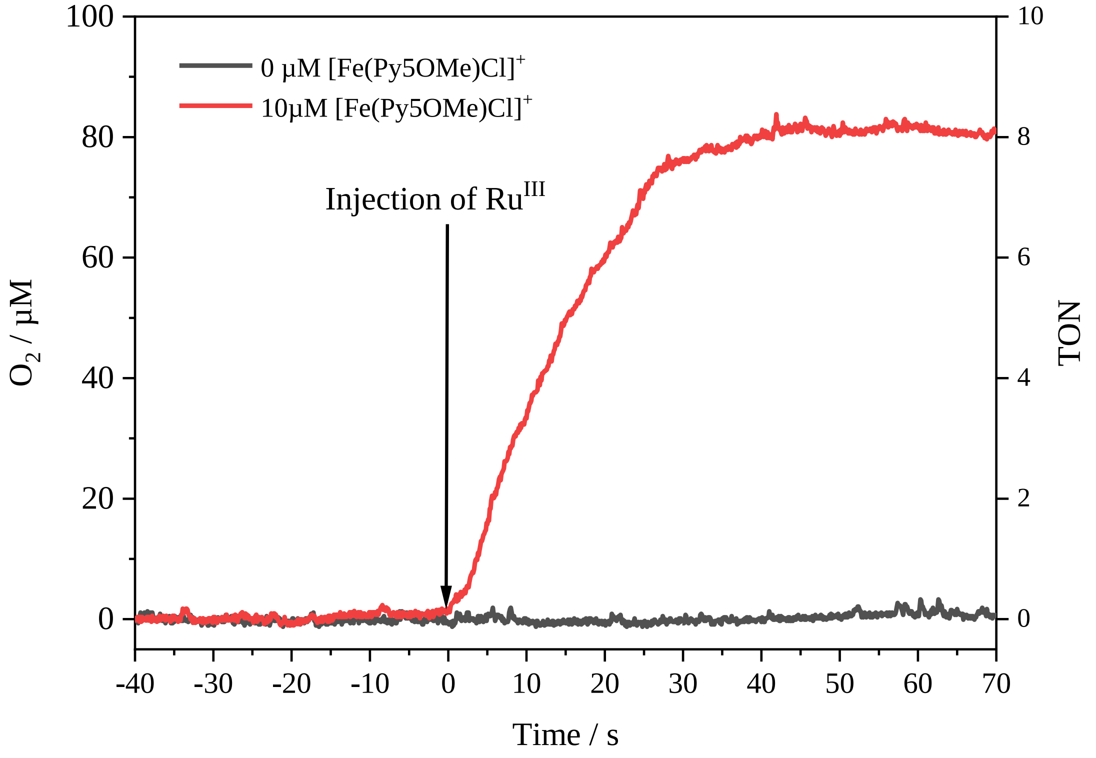

Figure S1. Oxygen evolution traces obtained after addition of $[Ru(bpy)_3]^{3+}$ (0.6 mM) to either a buffered solution of $[Fe(Py5OMe)Cl]^{+}$ (10 μM), red trace, or to a plain borate buffer (40 mM; pH 8.0), gray trace. Both solutions contained 0.2 % $^{v}/_{v}$ MeCN. The oxidizer $[Ru(bpy)_3]^{3+}$ 5 min after the complex $[Fe(Py5OMe)Cl]^{+}$ was dissolved in buffered solution (see Experimental methods for details).

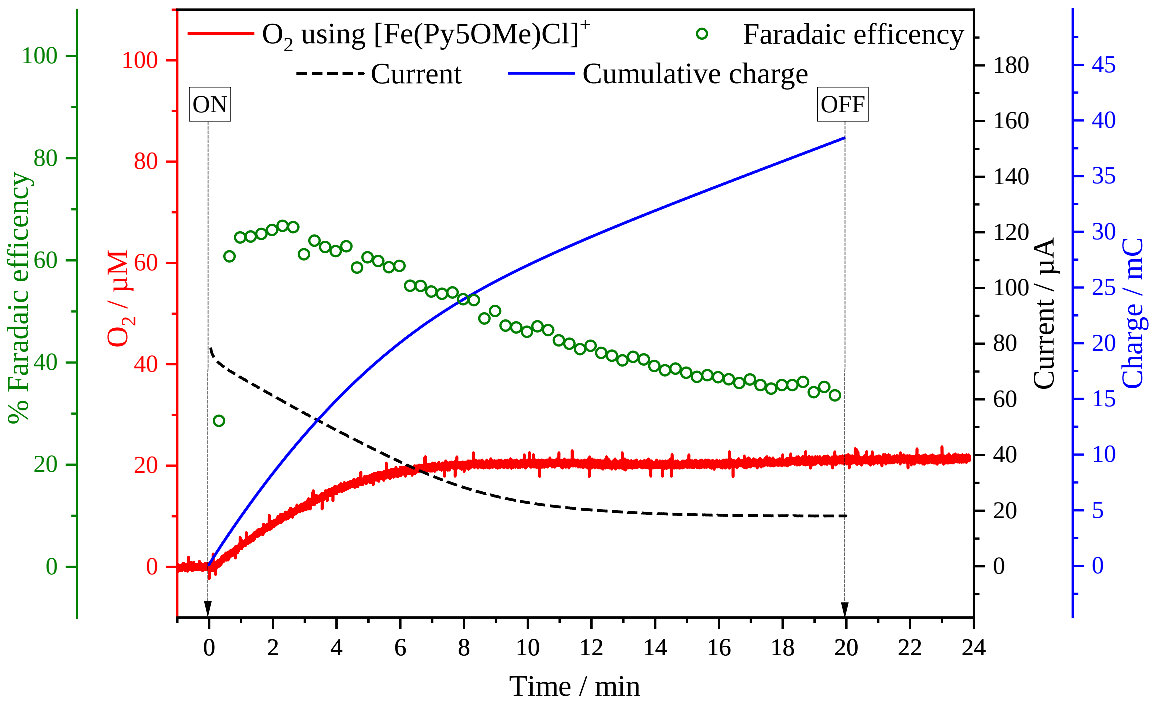

Figure S2. Electrocatalytic O_2 evolution of 0.5 mM $[Fe(Py5OMe)Cl]^+$ dissolved in 90 mM borate buffer pH 8.0 containing 10 % MeCN at an applied potential of 2.0 V (red trace). The corresponding current is shown as a dashed black line. The passed charge is depicted with a blue line and the corresponding calculated Faradaic efficiency as open green circles.

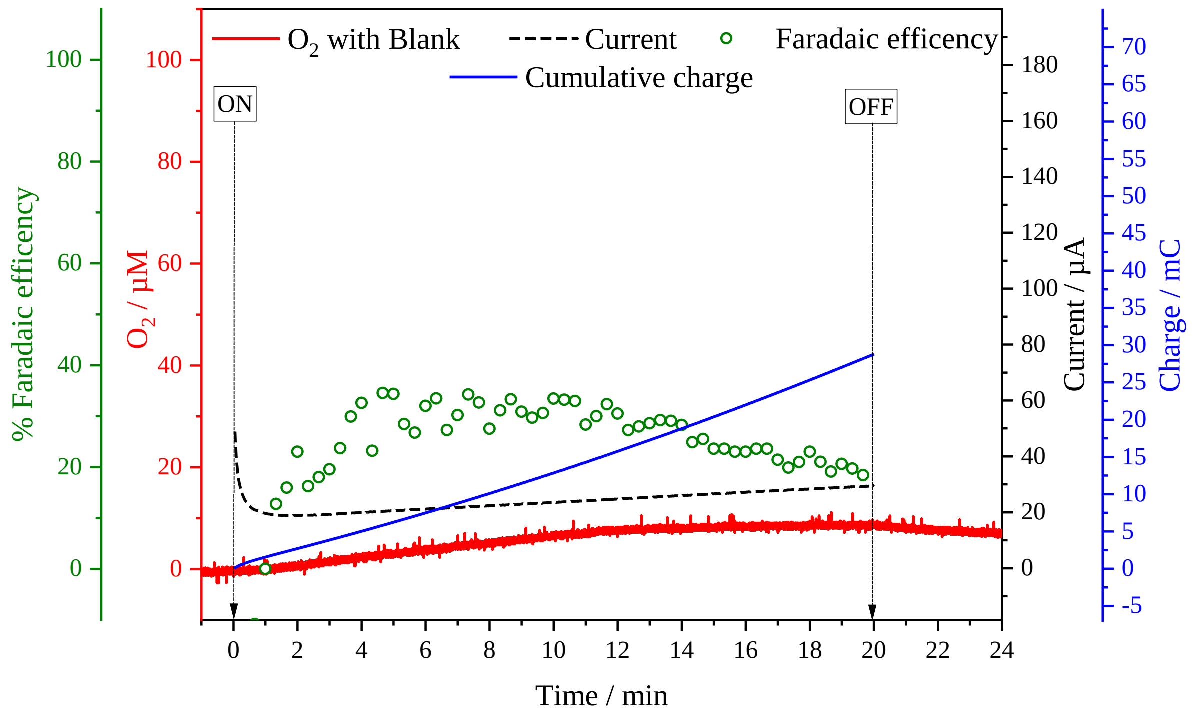

Figure S3. Electrocatalytic O_2 evolution of the electrolyte consisting in 90 mM borate buffer pH 8.0-10 % $^{v}/_{v}$ MeCN, red line, with an applied potential of 2.0 V. The corresponding current is shown as a dashed line. The passed charge is depicted with a blue line and the corresponding calculated Faradaic efficiency as open green circles.

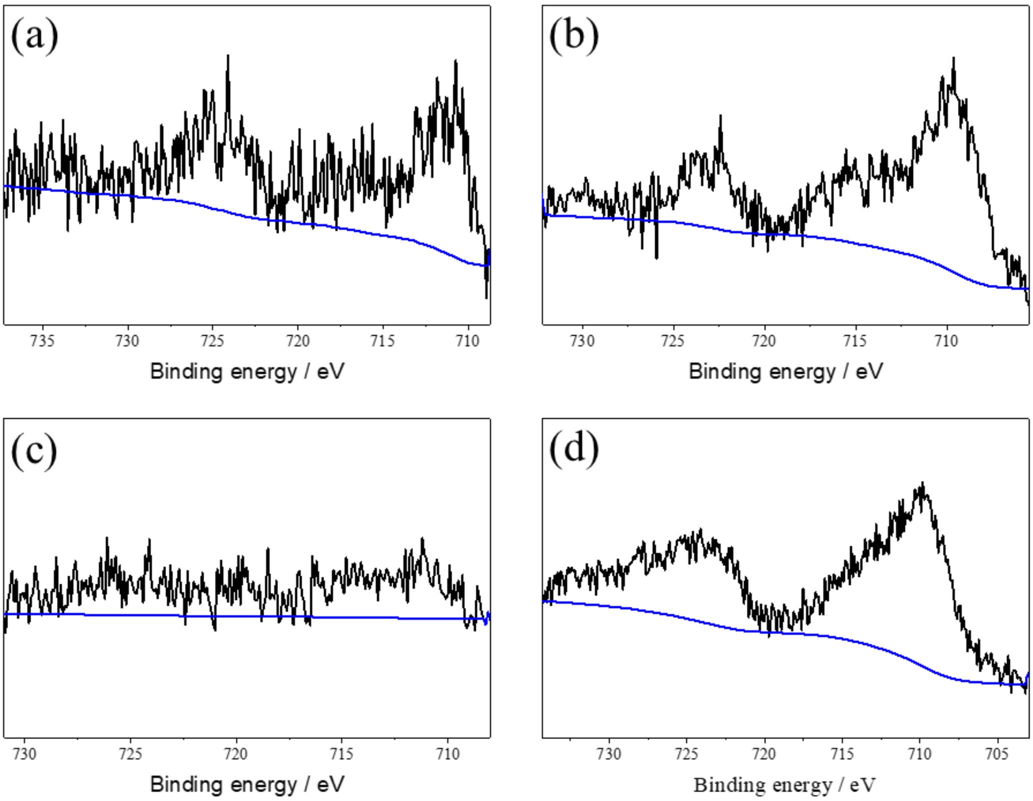

Figure S4. High resolution XPS spectra of the Fe 2p regions of (a) the glassy carbon electrode after CPE with $[Fe(Py5OMe)Cl]^+$, (b) $[Fe(Py5OMe)Cl]^+$ dropcasted onto a glassy carbon surface, (c) the glassy carbon electrode after CPE with $FeSO_4$, and (d) $FeSO_4$ in pH 8.0 dropcasted onto a glassy carbon surface. The blue line is the applied Shirley background correction. The binding energy shifts of the Fe $2p_{3/2}$ peaks are reported in **Table S2**.

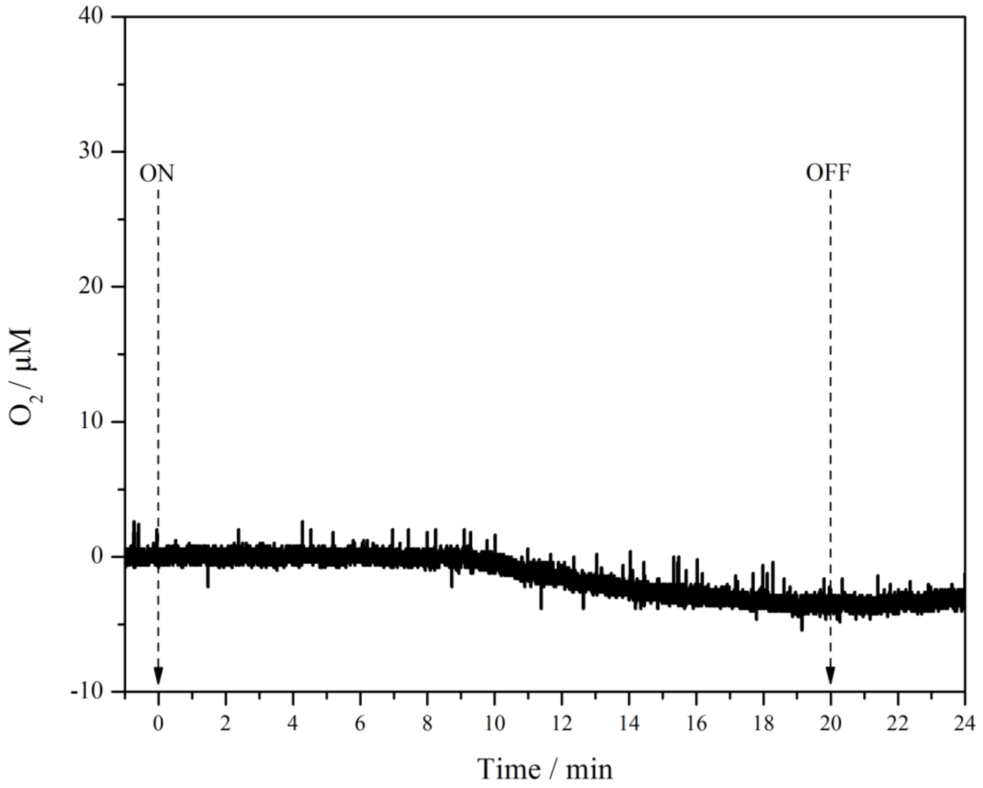

Figure S5. Electrocatalytic O_2 evolution comparison with rinsed electrode of previous electrolysis of 0.5 mM $[Fe(Py5OMe)]^+$ in neat electrolyte in 90 mM borate buffer pH 8.0 with 10 % $^v/_v$ MeCN. Applied potential 2.0 V.

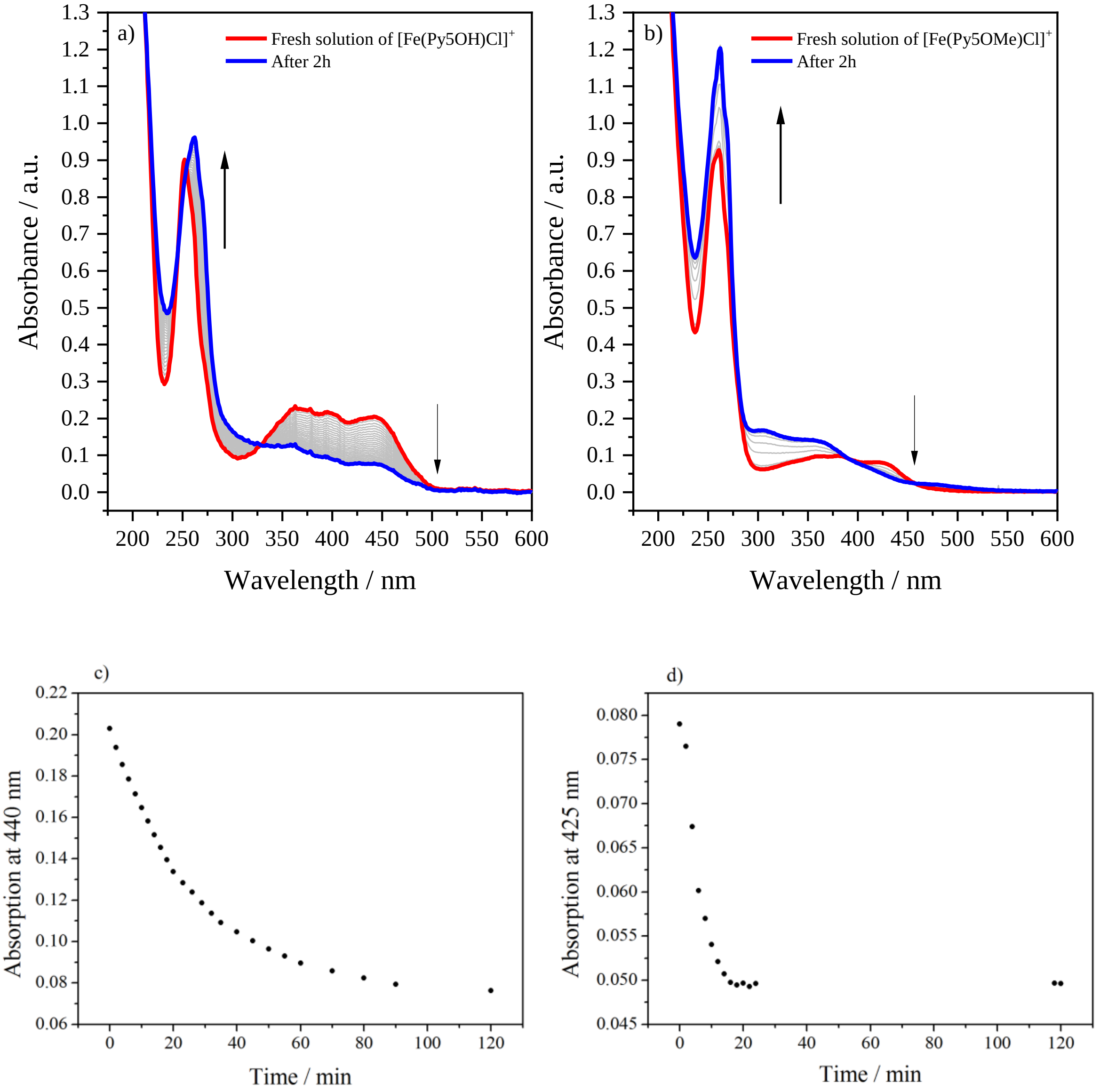

Figure S6. Evolution of UV-vis spectra of 50 µM $[Fe(Py5OH)Cl]^+$ (a) and 50 µM $[Fe(Py5OMe)Cl]^+$ (b) in 90 mM Borate buffer (pH 8.0, with 10 % MeCN) open to air. The initial spectra are shown in red, and the spectra recorded after 2 h are shown in blue. Decay of the absorption at 440 nm and 425 nm for $[Fe(Py5OH)Cl]^+$ and $[Fe(Py5OMe)Cl]^+$, respectively, are shown in (c) and (d).

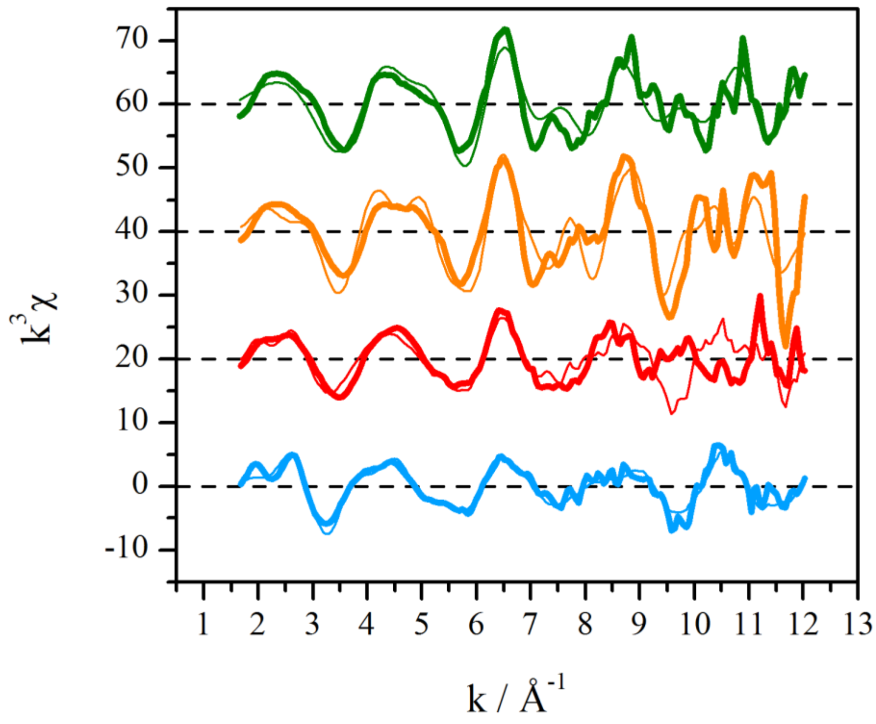

Figure S7. EXAFS spectra weighted by $k^3$of the 0.5mM [Fe(Py5OMe)Cl]$^+$ complex solution in different conditions. Complex in 5.6 % $^v/_v$ water-electrolyte solution, light blue line; complex after 2 minutes in 90 mM borate buffer (pH 8.0) with 10 % $^v/_v$ MeCN, red line; complex after 2 hours in 90 mM borate buffer (pH 8.0) with 10 % $^v/_v$ MeCN, orange line; complex oxidized with 6 equivalents of Ru(III) after 2 minutes from its preparation in 90 mM borate buffer (pH 8.0) with 10 % $^v/_v$ MeCN, green line. Spectra were recorded at 20 K and are offset for clarity. Simulations of the experimental data are shown as thin lines and the parameters are given in **Table S5**.

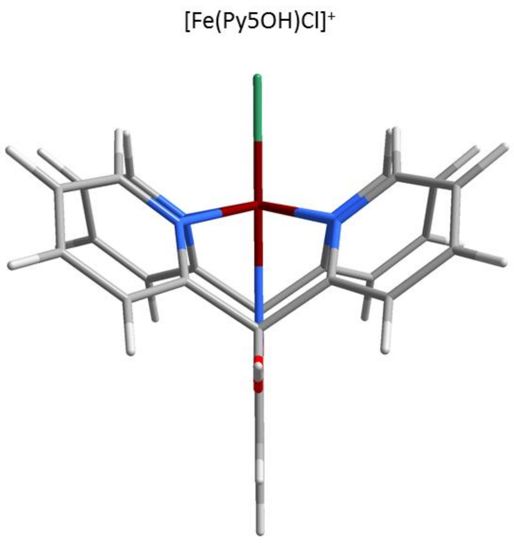

Figure S8. Molecular structures of the high-spin forms of $[Fe(Py5OH)Cl]^+$ (left)[4] and $[Fe(Py5OMe)Cl]^+$ (right).[5] Color code: Fe – brown, Cl – green, N – blue, O – red, C – dark grey, H – light grey.

4. Das, B.; Orthaber, A.; Ott, S.; Thapper, A., Iron Pentapyridyl Complexes as Molecular Water Oxidation Catalysts: Strong Influence of a Chloride Ligand and pH in Altering the Mechanism. *ChemSusChem* **2016**, *9*, 1178-1186.
5. Klein Gebbink, R. J. M.; Jonas, R. T.; Goldsmith, C. R.; Stack, T. D. P., A Periodic Walk: A Series of First-Row Transition Metal Complexes with the Pentadentate Ligand PY5. *Inorg. Chem.* **2002,** *41*, 4633-4641.

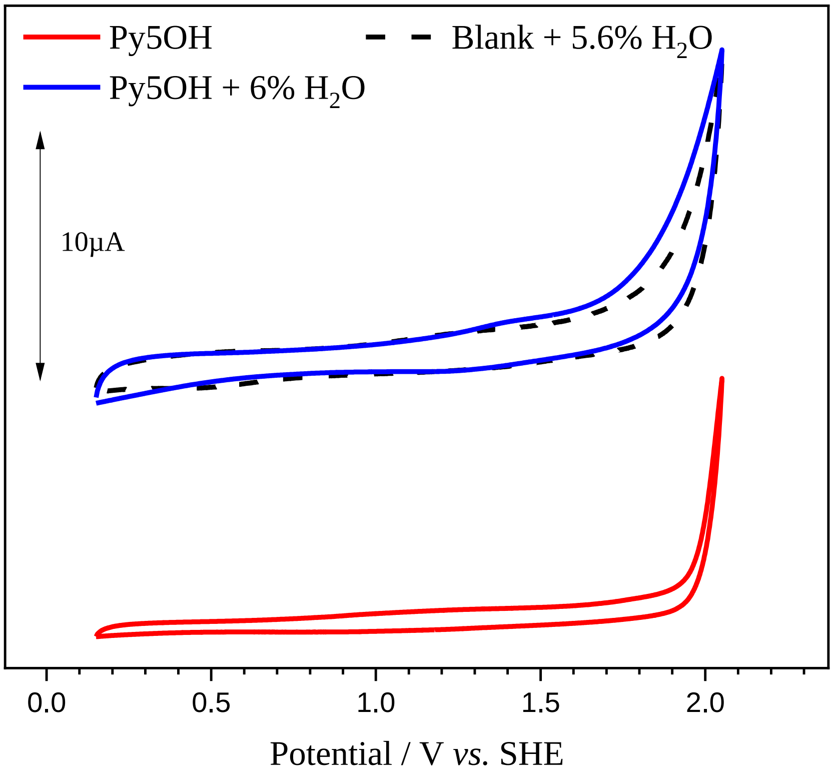

Figure S9. Cyclic Voltammetry of 0.5 mM Py5OH in dry MeCN and $TBAPF_6$ (0.1M), red line, and in 5.6 % $^{v}/_{v}$ of water, blue line. Blank with 5.6 % $^{v}/_{v}$ of water, black dashed line. Scan rate 100 mVs^{-1}.

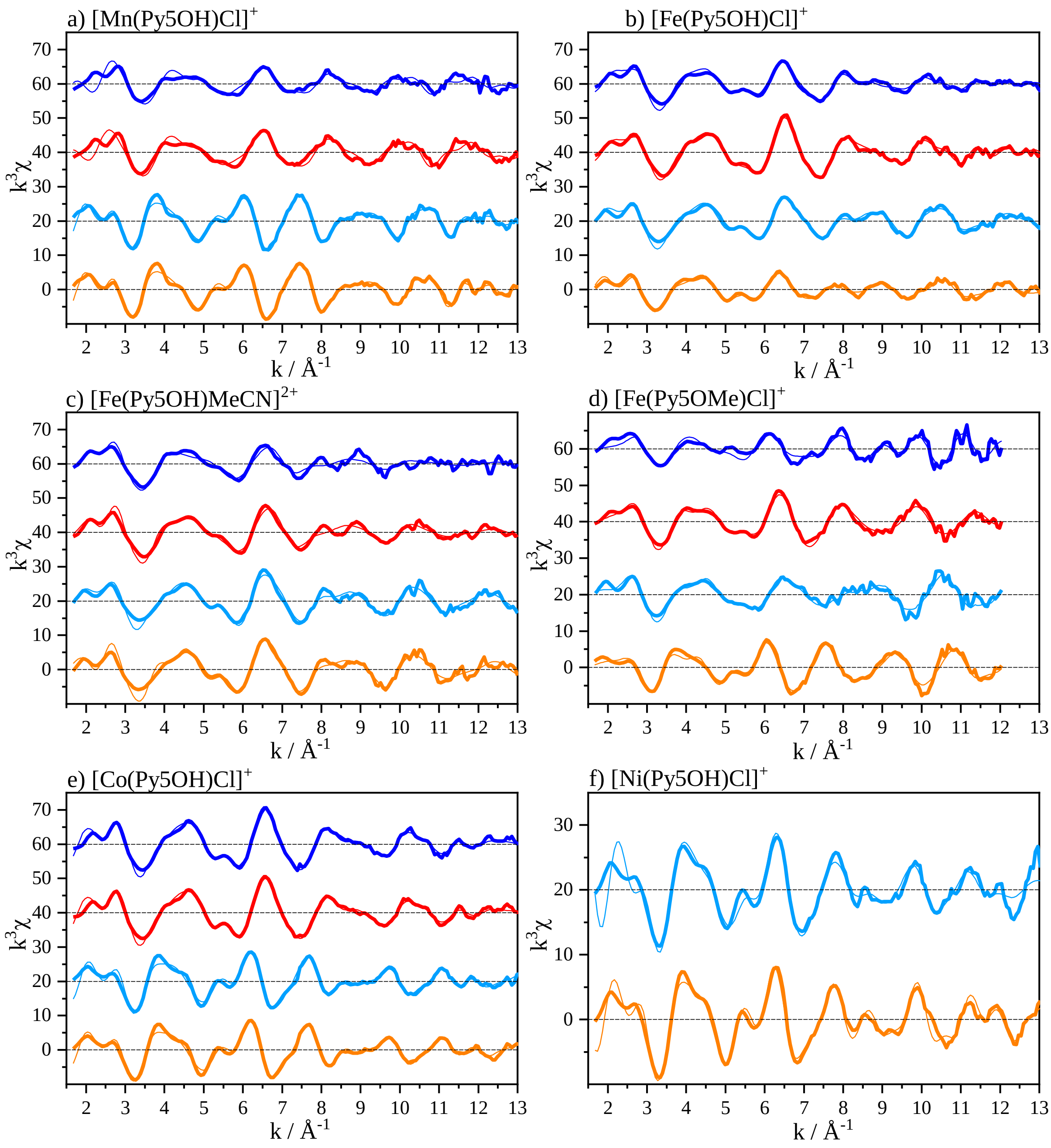

Figure S10. EXAFS spectra weighted by $k^3$of the studied complexes $[M(Py5OR)X]^+$ [a) M = Mn, R = H; b) M = Fe, R = H; c) M = Fe, R = H; d) M = Fe, R = Me; e) M = Co, R = H; f) M = Ni, R = H] in 1 mM dry MeCN + 0.1 M $TBAPF_6$, orange line; 0.5 mM complex in 5.6 % $^v/_v$ water-electrolyte solution, light blue line; 1 mM oxidized complex in electrolyte, red line; 0.5 mM oxidized complex in 5.6 % $^v/_v$ water-MeCN with the addition of $TBAPF_6$ (0.1 M), blue line. Spectra were recorded at 20 K and are offset for clarity. Simulations of the experimental data are shown as thin lines and the parameters are given in **Table S5**.

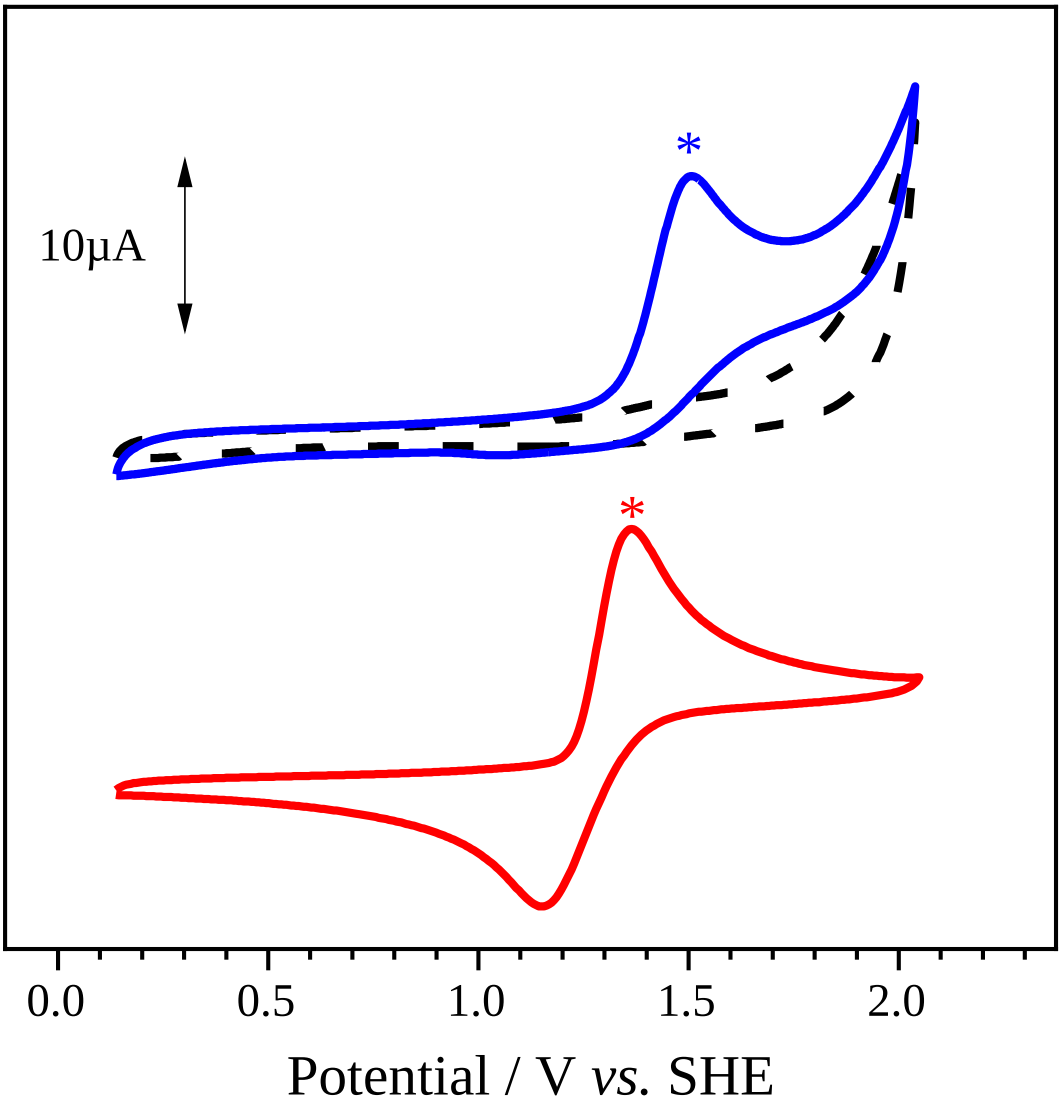

Figure S11. Cyclic Voltammetry of 0.5 mM TBACl in dry MeCN and $TBAPF_6$ (0.1M), red line, and in 5.6 % $^{v}/_{v}$ of water, blue line. Blank with 5.6 % $^{v}/_{v}$ of water, black dashed line. Scan rate 100 mVs^{-1}.

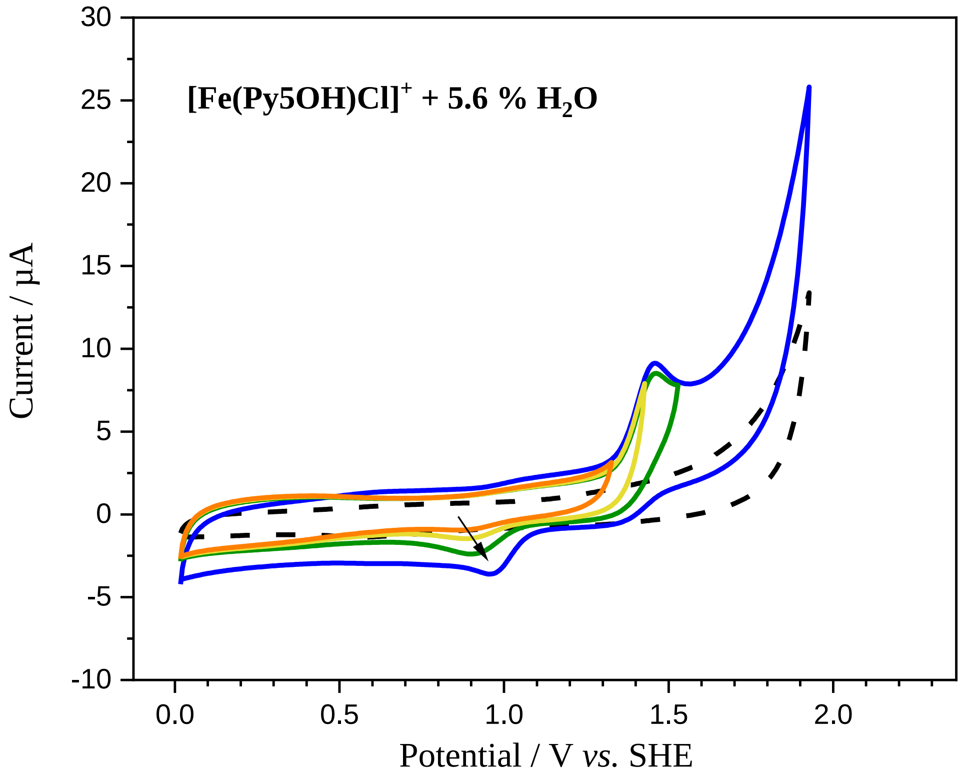

Figure S12. Cyclic voltammetry of 0.5 mM $[Fe(Py5OH)Cl]^+$ in 0.1M $TBAPF_6$ MeCN with 5.6 % $^v/_v$ water. Blank, black dashed line; scan to 1.93 V (blue line), 1.53 V (green line), 1.43 V (yellow line) and 1.33 V (orange line). Scan rate 100 mV s^{-1}.

Table S1. *Element composition ratios of glassy carbon (GC) electrodes as determined by XPS.*

Sample	O/C	Fe/C	O/Fe	N/Fe	Cl/Fe	S/Fe
**Pristine GC electrode**	0.17	0	-	-		--
**Dropcasted [Fe(Py5OMe)Cl]$^{+}$**	0.10	2.5 x 10^{-2}	4.12	4.97	1.06	--
**GC electrode after CPE with [Fe(Py5OMe)Cl]$^{+}$**	0.27	1.0 x 10^{-2}	26.52	2.53	--	--
**GC electrode after CPE with $FeSO_4$**	0.39	5.7 x 10^{-3}	68.53	2.73	--	0.75
**Dropcasted $FeSO_4$ in buffer pH 8.0**	3.17	2.5 x 10^{-1}	12.95	0.23	--	1.74

Table S2. *Binding energy of the Fe 2p$_{3/2}$ peak as determined by XPS high resolution spectra.*

Samples	Binding energy / eV
**Dropcasted [Fe(Py5OMe)Cl]$^{+}$**	709.7
**GC electrode after CPE with [Fe(Py5OMe)Cl]$^{+}$**	711.3
**GC electrode after CPE with $FeSO_4$**	712.8
**Dropcasted $FeSO_4$ in buffer pH 8.0**	710.0

Table S3. *DLS measurements. All samples were dissolved in borate buffer (pH 8.0). For the 10 µM iron samples the borate buffer was 40 mM with 0.2 % MeCN and for the 0.5 mM iron samples 90 mM borate buffer with 10 % MeCN was used. The error represents the standard deviation of the hydrodynamic diameter distribution of the particles. The maximal attenuator factor of 11 is used due to the absence of detectable nanoparticles in the concentration range of the instrument.*

Sample	Attenuator Factor	Particles diameter (nm)
Buffer	11	--
10 µM [Fe(Py5OMe)Cl]$^{+}$ + 0.6 mM Ru^{III}[a]	11	--
10 µM $FeSO_4$ + 0.6 mM Ru^{III}	11	--
3 mM Ru^{III}	11	--
0.5 mM [Fe(Py5OMe)Cl]$^{+}$ (2 min)[b]	11	--
0.5 mM [Fe(Py5OMe)Cl]$^{+}$ (2 h)[c]	8	900 ± 150
0.5 mM [Fe(Py5OMe)Cl]$^{+}$ + 3 mM Ru^{III}	8	700 ± 100
0.5 mM $FeSO_4$	7	2500 ± 1100
0.5 mM [Fe(Py5OH)Cl]$^{+}$ (2 min)[b]	11	--
0.5 mM [Fe(Py5OH)Cl]$^{+}$ (2 h)[c]	11	--
0.5 mM [Fe(Py5OH)Cl]$^{+}$ + 3 mM Ru^{III}	10	1100 ± 110
[Fe(Py5OMe)Cl]$^{+}$ after CPE[d]	8	640±40

[a] [Ru(bpy)$_3$](ClO$_4$)$_3$ [b] Fresh solution measured within 2 minutes. [c] Solution measured after 2 h aging. [d] The post-CPE solution was diluted twice with the same medium (final concentration: 0.25 mM) in order to have enough volume for the DLS analysis. The control experiment using 250 µM of $FeSO_4$ in the same medium also gives positive results for nanoparticle formation.

Table S4. *Geometric and electronic structure of selected complexes, including formal oxidation state, spin multiplicity, electron configuration, relative free energy at 298 K compared to a most stable structure, Mulliken spin on the metal atom, and metal-ligand bond distances. Orbitals are divided into π-type (d_{xy},d_{xz},d_{yz}) and σ-type orbitals ($d_{x^2-y^2}$, d_{z^2}). Includes also data from previous paper.[43] Only structures within 5.5 kcal/mol of the lowest energy structure are listed. In case only one structure is listed, the energy of the next lowest state is given in parenthesis.*

**Complex**	**Ox. state**	**Multiplicity**	**Electrons Configuration**	**Energy (kcal/mol)**	**Metal spin**	**Distances (Å)**			
						**Fe-N_{eq1}**	**Fe-N_{eq2}**	**Fe-N_{ax}**	**Fe-X**
**$[Mn(Py5OH)Cl]^{+}$ ‡**	II	Sextet	$\pi^{3}\sigma^{2}$	0.0 (21.8)	4.80	2.260	2.260	2.389	2.421
	III	Quintet	$\pi^{3}\sigma^{1}$	0.0 (6.7)	4.28	2.070	2.071	2.312	2.396
		Quintet§	$\pi^{3}\sigma^{1}$	0.6	4.19	2.046	2.270	2.116	2.264
**$[Fe(Py5OH)Cl]^{+}$ ‡**	II	Singlet	$\pi^{6}\sigma^{0}$	5.3	0.00	2.040	2.040	2.026	2.387
		Quintet	$\pi^{4}\sigma^{2}$	0.0	3.94	2.206	2.206	2.292	2.357
	III	Doublet	$\pi^{5}\sigma^{0}$	0.0	1.19	2.036	2.036	2.041	2.255
		Sextet	$\pi^{3}\sigma^{2}$	1.1	4.31	2.153	2.153	2.233	2.244
**$[Fe(Py5OMe)Cl]^{+}$ ‡**	II	Quintet	$\pi^{4}\sigma^{2}$	0.0 (7.4)	3.93	2.286	2.287	2.217	2.349
	III	Doublet	$\pi^{5}\sigma^{0}$	0.0	1.19	2.074	2.096	1.997	2.247
		Sextet	$\pi^{3}\sigma^{2}$	0.7	4.31	2.211	2.213	2.163	2.241
**$[Fe(Py5OH)MeCN]^{2+}$ ‡**	II	Singlet	$\pi^{6}\sigma^{0}$	0.0	0.00	2.040	2.040	2.032	1.933
		Quintet	$\pi^{4}\sigma^{2}$	3.2	3.80	2.186	2.188	2.218	2.125
	III	Doublet	$\pi^{5}\sigma^{0}$	0.0 (7.2)	1.22	2.014	2.015	2.026	1.945
**$[Fe(Py5OMe)MeCN]^{2+}$**	II	Singlet	$\pi^{6}\sigma^{0}$	0.0	0.00	2.089	2.027	1.990	1.935
		Quintet	$\pi^{4}\sigma^{2}$	3.8	3.90	2.117	2.247	2.163	2.117
	III	Doublet	$\pi^{5}\sigma^{0}$	0.0 (6.2)	1.25	2.008	2.071	1.971	1.941
**$[Fe(Py5OH)H_2O]^{2+}$ ‡**	II	Singlet	$\pi^{6}\sigma^{0}$	0.0	0.00	2.031	2.032	1.999	2.055
		Quintet	$\pi^{4}\sigma^{2}$	2.5	3.97	2.189	2.199	2.190	2.171
	III	Doublet	$\pi^{5}\sigma^{0}$	0.0 (8.0)	1.25	2.023	2.024	1.991	1.969
**$[Fe(Py5OMe)H_2O]^{2+}$**	II	Singlet	$\pi^{6}\sigma^{0}$	0.0	0.00	2.016	2.078	1.959	2.057
		Quintet	$\pi^{4}\sigma^{2}$	0.5	3.93	2.164	2.193	2.144	2.159
	III	Doublet	$\pi^{5}\sigma^{0}$	.0.0	1.19	2.066	2.048	1.949	1.964
		Sextet	$\pi^{3}\sigma^{2}$	4.5	4.42	2.121	2.160	2.071	2.086
**$[Fe(Py5OH)OH]^{+}$**	II	Singlet	$\pi^{6}\sigma^{0}$	5.1	0.00	2.020	2.020	2.029	1.952
		Quintet	$\pi^{4}\sigma^{2}$	0.0	3.89	2.029	2.222	2.368	1.915
	III	Doublet	$\pi^{5}\sigma^{0}$	0.0	1.10	2.021	2.024	2.064	1.807
		Sextet	$\pi^{3}\sigma^{2}$	4.3	4.33	2.149	2.164	2.274	1.821
**$[Fe(Py5OMe)OH]^{+}$**	II	Quintet	$\pi^{4}\sigma^{2}$	0.0 (7.0)	3.85	2.202	2.283	2.290	1.907
	III	Doublet	$\pi^{5}\sigma^{0}$	0.0	1.08	2.007	2.065	2.020	1.810
		Sextet	$\pi^{3}\sigma^{2}$	2.9	4.29	2.158	2.225	2.206	1.818
**$[Co(Py5OH)Cl]^{+}$ ‡**	II	Doublet	$\pi^{6}\sigma^{1}$	5.5	1.06	2.015	2.015	2.209	2.614
		Quartet	$\pi^{5}\sigma^{2}$	0.0	2.83	2.168	2.168	2.222	2.392
	III	Singlet	$\pi^{6}\sigma^{0}$	0.0 (18.1)	0.00	2.009	2.009	2.020	2.263
**$[Co(Py5OH)MeCN]^{2+}$**	II	Doublet	$\pi^{6}\sigma^{1}$	1.8	0.97	2.017	2.017	2.199	2.227

		Quartet	$\pi^{5}\sigma^{2}$	0.0	2.78	2.158	2.158	2.171	2.113
	III	Singlet	$\pi^{6}\sigma^{0}$	0.0 (27.5)	0.0	2.003	2.004	1.993	1.909
**[Ni(Py5OH)Cl]$^{+}$ ‡**	II	Triplet	$\pi^{6}\sigma^{2}$	0.0 (19.0)	1.61	2.131	2.131	2.150	2.409
	III	Doublet	$\pi^{6}\sigma^{1}$	0.0 (16.7)	0.84	1.993	1.993	2.193	2.420
**[Ni(Py5OH)MeCN]$^{2+}$**	II	Triplet	$\pi^{6}\sigma^{2}$	0.0	1.61	2.125	2.126	2.118	2.077

§ Quintet of the distorted [Mn^{III}(Py5OH)Cl]$^{2+}$ structure that fits best with EXAFS simulation. ‡ Data reported in reference [12].

Table S5. *Metal-ligand distances (R / Å) and Debye-Waller factors (σ / Å) of shells used in the EXAFS simulations for data collected at 20 K. Coordination numbers were fixed at values from the crystal structures. Numbers in parentheses show the uncertainty in the last digit corresponding to the 68 % confidence interval of the fit parameter, obtained from the covariance matrix of the Levenberg-Marquardt fit. Phase functions were generated by the FEFF9.0 software using the XRD structures (for all reduced complexes except [Fe^{II}(Py5OH)Cl]$^{+}$) or the corresponding lowest-energy DFT-geometry-optimized structure (for all oxidized complexes and [Fe^{II}(Py5OH)Cl]$^{+}$). Multiple-scattering shells (several hundred for each simulation) had the same σ, and R was not fitted.*

**Complex**	**Solvent**	**Ox. state**	**5 M–N**<br>**R**<br>σ	**1 M–Cl**<br>**R**<br>σ	**12 M–C**<br>**R**<br>σ	**10 M–C**<br>**R**<br>σ	**Multiple-scattering shells**<br>σ
**[Mn(Py5OH)Cl]$^{+}$**	Dry MeCN	II	2.247(8)<br>0.065(4)	2.43(1)<br>0.057(6)	3.164(8)<br>0.073(5)	4.48(5)<br>0.081(8)	0.062(5)
		III	2.12(1)<br>0.085(4)	2.16(1)<br>0.044(5)	2.97, 3.16(1) [a]<br>0.067(6)	4.29(5)<br>0.054(8)	0.070(6)
		III [b]	3x2.06(1), 2x2.27<br>0.085(4)	2.22(1)<br>0.055	6x2.98, 6x3.16<br>0.064	6x4.30, 4x4.48<br>0.064	0.064
	MeCN +5.6 % H_2O	II	2.247(8)<br>0.063(4)	2.41(1)<br>0.059(7)	3.160(8)<br>0.074(5)	4.52(3)<br>0.08(1)	0.075(7)
		III	2.09(2)<br>0.14(2)	2.19(1)<br>0.060(5)	2.97, 3.16(2) [a]<br>0.08(1)	4.32(4)<br>0.08(1)	0.08(1)
**[Fe(Py5OH)Cl]$^{+}$**	Dry MeCN	II [c]	1.993(3)<br>0.067(3)	2.378(6)<br>0.050(6)	2.958(6)<br>0.085(4)	4.25(3)<br>0.085(4)	0.083(6)
		III	1.990(3)<br>0.062(3)	2.218(6)<br>0.051(8)	2.950(6)<br>0.091(6)	4.28(3)<br>0.091(6)	0.058(8)
	MeCN +5.6 % H_2O	II	(66(4) % [Fe(II)Py5OH-MeCN] in MeCN + 34(4) % [Fe(II)PyOH-Cl] in MeCN)				
		III	2.02(1)<br>0.095(5)	0.4(1)x2.16(1) [h]<br>0.051(8)	2.95(1)<br>0.11(1)	4.23(4)<br>0.11(1)	0.068(9)
**[Fe(Py5OH)MeCN]$^{2+}$**	Dry MeCN	II [d]	1.973(3)<br>0.062(4)	--	2.930(5)<br>0.068(8)	4.15(6)<br>0.14(3)	0.074(7)
		III [d]	1.968(4)<br>0.083(2)	--	2.943(7)<br>0.084(4)	4.20(9)<br>0.18(4)	0.097(6)
	MeCN +5.6 % H_2O	II [d]	1.980(3)<br>0.065(4)	--	2.936(7)<br>0.087(7)	4.04(6)<br>0.08(1)	0.15(2)
		III [e]	O: 2x1.88(2),<br>N: 4x2.12(1)<br>0.05(1)	--	3.00(3)<br>0.11(2)	4.20<br>0.18	0.15(4)
**[Fe(Py5OMe)Cl]$^{+}$**	Dry	II	2.216(7)<br>0.054(4)	2.31(1)<br>0.045	3.14(1)<br>0.083(5)	4.42<br>0.1	0.15(4)

	MeCN	III	2.10(1)<br>0.085(6)	2.22(1)<br>0.055	3.03(1)<br>0.098(8)	4.30<br>0.1	0.09(2)
	MeCN +5.6 % H_2O	II [f]	1.977(5)<br>0.043(4)	--	2.94(1)<br>0.1	4.20<br>0.1	0.15
		III	3x2.144(7)<br>0.059(5)	--	6x3.11(1)<br>0.071	Fe-Fe shell:<br>2x3.33(1)<br>0.059	0.10
	Borate buffer (2h)	III [g]	Fe-O shells:<br>6x1.93, 6x3.66<br>0.061(5)	--	Fe-Fe shell:<br>6x2.99(1)<br>0.080(3)	Fe-Fe shell:<br>6x5.00(1)<br>0.067(4)	--
	After Ru(III) oxidation	III [g]	Fe-O shell:<br>6x1.92(1)<br>0.078(5)	--	Fe-Fe shell:<br>2.3(1)x2.89(1)<br>0.041(3)	Fe-Fe shell:<br>3.7(1)x3.05(1)<br>0.041(3)	--
**[Co(Py5OH)Cl]$^{+}$]**	Dry MeCN	II	2.141(5)<br>0.059(2)	2.40(1)<br>0.075(5)	3.075(7)<br>0.070(3)	4.39(2)<br>0.070(3)	0.076(6)
		III	1.969(4)<br>0.065(3)	2.20(1)<br>0.065(3)	2.923(8)<br>0.082(4)	4.27(4)<br>0.082(4)	0.095(7)
	MeCN +5.6 % H_2O	II	2.141(5)<br>0.063(3)	2.38(2)<br>0.088(9)	3.072(7)<br>0.071(4)	4.40(2)<br>0.071(4)	0.076(6)
		III	1.971(5)<br>0.065(4)	2.20(1)<br>0.065(4)	2.925(8)<br>0.081(4)	4.27(4)<br>0.081(4)	0.095(8)
**[Ni(Py5OH)Cl]$^{+}$**	Dry MeCN	II	2.092(5)<br>0.062(2)	2.42(1)<br>0.075(4)	3.031(8)<br>0.080(3)	4.38(3)<br>0.086(6)	0.076(5)
	MeCN +5.6 % H_2O	II	2.099(5)<br>0.069(4)	0.0(1)x- [h]	3.03(1)<br>0.086(8)	4.34(5)<br>0.086(8)	0.075(7)

[a]For a reasonable fit, the first Mn-C shell had to be split into two shells with coordination numbers 6+6. [b]Alternative EXAFS model based on the DFT-optimized structure ^{5}[Mn^{III}(Py5OH)Cl]$^{+}$that includes more shells; only the parameters with given errors were fitted, other parameters were kept to the values from the DFT-optimized structure. [c]EXAFS model for [Fe(Py5OH)Cl]$^{+}$fitted to data from powder sample only; data from sample dissolved in MeCN was fitted as a linear combination of 40 % of the powder sample + 40 % of the powder at 150 K + 20 % of the [Fe^{II}(Py5OH)MeCN]$^{2+}$ sample. [d]For [Fe^{II}(Py5OH)MeCN]$^{2+}$, the coordination number of Fe–N shells was 6 and that of the first Fe–C shells was 13, simulating MeCN coordination to Fe. [e]For [Fe^{III}(Py5OH)MeCN]$^{3+}$in MeCN + 5.6 % $^{v}/_{v}$ water, the listed shells corresponded to a minority fraction (31 % +/-4 %), with a majority fraction (69 %) identical to [Fe^{III}(Py5OH)MeCN]$^{3+}$in dry MeCN added separately. [f]For [Fe^{II}(Py5OMe)Cl]$^{+}$ in MeCN + 5.6 % $^{v}/_{v}$ water, the listed shells corresponded to a majority fraction (66 % +/-3 %), with a minority fraction (34 %) identical to [Fe^{II}(Py5OMe)Cl]$^{+}$ in dry MeCN added separately. [g]For [Fe^{II}(Py5OMe)Cl]$^{+}$ in borate buffer, only Fe–O and Fe–Fe shells were used. [h]The coordination number N_{Cl} of the M–Cl shell was fitted, and the coordination numbers of the M–N shell and first M-C shell were set to 6 – N_{Cl} and 13 – N_{Cl} correspondingly, simulating partial MeCN/Cl coordination to the metal center.

Table S6. *Summary of the species detected with XAS measurements at 20 K as explained in the text. The spin state can be estimated form the EXAFS for Fe(II) complexes. For all the other cases the spin state is determined by DFT calculations.*

Complex	Solid	MeCN + $TBAPF_6$		5.6 % H_2O/MeCN + $TBAPF_6$	
	**Reduced**	**Reduced**	**Oxidized**	**Reduced**	**Oxidized**
**$[Mn(Py5OH)Cl]^+$**	100 % ^{6}Mn–Cl HS	100 % ^{6}Mn-Cl HS	100 % ^{5}Mn-Cl HS	100 % ^{6}Mn–Cl HS	100 % ^{5}Mn–Cl HS^a
**$[Fe(Py5OH)Cl]^+$**	100 % ^{5}Fe–Cl HS	40 % ^{5}Fe-Cl, HS<br>40 % ^{1}Fe-Cl LS<br>20 % ^{1}Fe-MeCN LS	100 %, ^{2}Fe–Cl LS	12 % ^{5}Fe–Cl, HS<br>12 % ^{1}Fe–Cl, LS<br>76 % ^{1}Fe–MeCN LS	40 % ^{2}Fe–Cl LS<br>40 % $^{5\text{-}2}$Fe–MeCN HS or LS^b<br>20 % $^{5\text{-}2}$Fe–O HS
**$[Fe(Py5OH)Solv]^{2+}$**	100 % ^{1}Fe–MeOH LS	100 % ^{1}Fe-MeCN LS	100 % $^{5\text{-}2}$Fe–MeCN HS or LS	100 % ^{1}Fe–MeCN LS	60 % Fe-MeCN LS<br>40 %, Fe-OH_x LS
**$[Fe(Py5OMe)Cl]^+$**	100 % ^{5}Fe–Cl HS	100 % ^{5}Fe–Cl HS	100 % $^{5\text{-}2}$Fe–Cl HS or LS^b	34 % ^{5}Fe–Cl HS<br>66 % ^{1}Fe–MeCN LS	50 % Fe–MeCN or –Cl^c<br>50 % mono-µ-oxo Fe–Fe species
**$[Co(Py5OH)Cl]^+$**	100 % ^{4}Co–Cl HS	100 % ^{4}Co–Cl HS	100 % ^{1}Co–Cl LS	100 % ^{4}Co–Cl HS	100 % ^{4}Co–Cl HS
**$[Ni(Py5OH)Cl]^+$**	100 % ^{3}Ni–Cl HS	100 % ^{3}Ni–Cl HS	--	100 % ^{3}Ni–MeCN HS	--

^{a} Other species are present in small amounts, making the spectrum not identical to the anhydrous oxidized sample. ^{b} EXAFS does not allow a unique assignment of the spin state. However, DFT calculations suggest that the LS configuration is 0.7 kcal mol^{-1} lower in energy than the HS state. ^{c} The presence or absence of a Cl-ligand cannot be determined with XAS for this sample.

Table S7. *Half-potentials in V vs. SHE of MPy5OH/MPy5OMe complexes calculated using DFT(B3LYP*) and the SMD solvation model.*

Complex	Solvent model					
	SMD MeCN		SMD water			
	Ligand		Ligand			
	Cl^-	MeCN	Cl^-	MeCN	H_2O	OH^-
[Mn-Py5OH-X]	0.74	--	0.66	--	--	--
[Fe-Py5OH-X]	0.77	1.16	0.68	1.03	0.79	0.13
[Fe-Py5OMe-X]	0.88	1.16	0.83	1.05	0.80	0.17
[Co-Py5OH-X]	0.59	0.77	0.50	0.62	--	--
[Ni-Py5OH-X]	1.59	--	1.50	--	--	--

Coordinates of the DFT Optimized Geometry

[1][Fe^{II}(Py5OMe)MeCN]$^{2+}$

68

Fe	-0.000110	0.652756	0.024753
O	-3.565725	-2.056399	-0.200604
C	-3.688206	-2.957248	-1.318401
N	0.001089	-1.329276	-0.149650
N	1.514470	0.764191	-1.317985
O	3.568650	-2.052197	-0.200584
C	3.692038	-2.953159	-1.318186
N	1.563296	0.476638	1.399206
N	-1.513901	0.761221	-1.319151
N	-1.563817	0.475340	1.398548
C	-1.670207	1.250472	2.487791
H	-0.913917	2.000388	2.622701
C	1.668184	1.250792	2.489272
H	0.910937	1.999645	2.624616
C	2.697970	1.142772	3.406143
H	2.714944	1.795946	4.265309
C	3.686358	0.196452	3.188480
H	4.504671	0.076771	3.883925
C	3.617512	-0.584626	2.044593
H	4.374015	-1.315118	1.813191
C	2.552880	-0.408889	1.175521
C	2.456092	-1.198060	-0.136632
C	1.157343	-1.999729	-0.043134
C	1.193259	-3.343989	0.295287
H	2.133779	-3.837412	0.457953
C	0.002666	-4.021668	0.481496
H	0.003284	-5.060147	0.778846
C	-1.188735	-3.345322	0.295585
H	-2.128672	-3.839798	0.458443
C	-1.154408	-2.001045	-0.042908
C	-2.454167	-1.200977	-0.136615
C	-1.645650	1.778590	-2.184617
H	-0.875883	2.524477	-2.171798
C	-2.723382	1.929359	-3.034419
H	-2.757281	2.771827	-3.708264
C	-3.749149	1.003011	-2.964039
H	-4.626295	1.090537	-3.588722
C	-3.645369	-0.028940	-2.046082
H	-4.457902	-0.721182	-1.928193
C	-2.552252	-0.411474	1.175167
C	-2.700243	1.142015	3.404305
H	-2.718468	1.796017	4.262813
C	-3.687311	0.194183	3.187120
H	-4.505757	0.074113	3.882340
C	-3.617041	-0.587804	2.043945
H	-4.372584	-1.319375	1.812805
C	2.510762	-0.136991	-1.252498
C	3.646258	-0.024352	-2.045735
H	4.459077	-0.716380	-1.928662
C	3.749654	1.008644	-2.962573
H	4.626685	1.097054	-3.587294
C	2.723810	1.934992	-3.031544
H	2.757595	2.778465	-3.704138
C	1.646135	1.782883	-2.181906
H	0.876620	2.529021	-2.167544
C	-2.509742	-0.140367	-1.252857
H	-3.651736	-3.982078	-0.955883
H	-2.901215	-2.811829	-2.056111
H	-4.654668	-2.789154	-1.788245
H	3.656616	-3.977957	-0.955462
H	4.658336	-2.784197	-1.788059
H	2.904901	-2.808700	-2.055928
N	-0.002474	2.573072	0.262178
C	-0.005014	3.710624	0.404549
C	-0.009267	5.145193	0.586076
H	1.011931	5.501967	0.711807
H	-0.453140	5.623481	-0.286069
H	-0.591699	5.400355	1.470407

$^{5}[Fe^{II}(Py5OMe)MeCN]^{2+}$

68

Fe	-0.006054	0.773710	0.095154
O	-3.538323	-2.122106	-0.387610
C	-3.500567	-3.003073	-1.524878
N	0.010675	-1.350899	-0.311149
N	1.593177	0.833671	-1.380446
O	3.564759	-2.089360	-0.402765
C	3.529656	-2.972692	-1.538127
N	1.747691	0.398409	1.468371
N	-1.648431	0.865992	-1.331073
N	-1.705621	0.341985	1.500209
C	-1.834389	0.995030	2.660909
H	-1.045494	1.687490	2.910784
C	1.894545	1.073134	2.614212
H	1.120273	1.786320	2.851744
C	2.978361	0.896825	3.457726
H	3.046050	1.462922	4.374342
C	3.959736	-0.011130	3.087576
H	4.822887	-0.177306	3.716456
C	3.826216	-0.703457	1.890589
H	4.570753	-1.409475	1.563219
C	2.704797	-0.471429	1.109693
C	2.493009	-1.191695	-0.238911
C	1.172979	-1.975958	-0.091177
C	1.215852	-3.264740	0.423749
H	2.159603	-3.727271	0.657803
C	0.022195	-3.916605	0.684433
H	0.026209	-4.909713	1.110150
C	-1.176434	-3.275999	0.421361
H	-2.115604	-3.747709	0.655027
C	-1.145948	-1.986105	-0.094511
C	-2.474940	-1.213178	-0.229717
C	-1.814176	1.911697	-2.150907
H	-1.064250	2.684396	-2.095268
C	-2.893636	2.041373	-3.003629
H	-2.975626	2.903350	-3.648119
C	-3.863898	1.051984	-2.975190
H	-4.739349	1.116260	-3.605535
C	-3.714333	-0.017448	-2.105041
H	-4.486626	-0.762448	-2.037902
C	-2.675599	-0.509677	1.129342
C	-2.917115	0.818468	3.505498
H	-2.971508	1.366092	4.434037
C	-3.913304	-0.067403	3.122067
H	-4.775966	-0.232884	3.751768
C	-3.794930	-0.740545	1.912317
H	-4.549698	-1.431853	1.576950
C	2.563598	-0.090916	-1.325585
C	3.695569	0.015727	-2.127984
H	4.495788	-0.697170	-2.038284
C	3.801256	1.064401	-3.028921
H	4.675161	1.149147	-3.658954
C	2.784580	2.004475	-3.093979
H	2.826476	2.845055	-3.769761
C	1.708944	1.854156	-2.240323
H	0.918537	2.586259	-2.222711
C	-2.579491	-0.099097	-1.303303
H	-3.120632	-3.981680	-1.238228
H	-2.882691	-2.608670	-2.330555
H	-4.524301	-3.107157	-1.875763
H	3.160239	-3.954231	-1.247683
H	4.552668	-3.067392	-1.893707
H	2.904579	-2.585486	-2.341762
N	-0.009878	2.847351	0.522727
C	-0.014994	3.976674	0.723460
C	-0.023030	5.399816	0.972896
H	0.998790	5.760617	1.079748
H	-0.503849	5.912168	0.140581
H	-0.576824	5.604376	1.888283

$^{2}[Fe^{III}(Py5OMe)MeCN]^{3+}$

68

Fe	-0.000622	-0.635145	-0.015527
O	-3.565901	2.057727	0.152206
C	-3.705858	2.992706	1.246380
N	0.002700	1.332102	0.100772
N	1.500237	-0.734504	1.315932
O	3.573053	2.047594	0.152238
C	3.712286	2.987674	1.242096
N	1.556910	-0.504936	-1.376021
N	-1.504176	-0.733427	1.311496
N	-1.556350	-0.500121	-1.376415
C	-1.651927	-1.315556	-2.438817
H	-0.899575	-2.070593	-2.552861
C	1.649285	-1.318934	-2.439825
H	0.894158	-2.070972	-2.555238
C	2.676391	-1.240163	-3.360244
H	2.686447	-1.920895	-4.197267
C	3.668162	-0.291646	-3.175433
H	4.483981	-0.197394	-3.877126
C	3.609177	0.524791	-2.055755
H	4.370048	1.257426	-1.846690
C	2.550232	0.382916	-1.178899
C	2.461961	1.206149	0.109462
C	1.164185	1.998729	-0.008698
C	1.197617	3.336749	-0.358535
H	2.139167	3.827792	-0.520092
C	0.007352	4.011765	-0.555379
H	0.009178	5.046471	-0.864541
C	-1.185272	3.340632	-0.359498
H	-2.125015	3.834767	-0.521952
C	-1.156461	2.002643	-0.009027
C	-2.456684	1.213942	0.108630
C	-1.623987	-1.745297	2.188009
H	-0.868476	-2.502998	2.172181
C	-2.685649	-1.871146	3.057915
H	-2.714275	-2.707045	3.739377
C	-3.700973	-0.933347	2.997296
H	-4.565107	-1.003112	3.641514
C	-3.608273	0.085877	2.063580
H	-4.415641	0.784596	1.952782
C	-2.546761	0.391264	-1.180077
C	-2.678875	-1.234424	-3.359185
H	-2.691695	-1.916540	-4.195041
C	-3.667022	-0.281838	-3.175772
H	-4.482560	-0.185667	-3.877528
C	-3.605121	0.535870	-2.057151
H	-4.363504	1.271309	-1.848847
C	2.494366	0.171149	1.247222
C	3.608800	0.075494	2.065790
H	4.420341	0.768860	1.952284
C	3.695114	-0.940298	3.003814
H	4.558870	-1.012916	3.648226
C	2.672989	-1.870285	3.069569
H	2.695332	-2.702401	3.755881
C	1.612427	-1.741729	2.198708
H	0.850654	-2.493046	2.188632
C	-2.493384	0.177786	1.245266
H	-3.673460	4.004679	0.851385
H	-2.925609	2.872107	1.995173
H	-4.677564	2.828463	1.704821
H	3.682315	3.997707	0.841925
H	4.682802	2.824197	1.703347
H	2.930236	2.872223	1.989806
N	-0.002258	-2.575398	-0.196150
C	-0.003926	-3.712625	-0.328668
C	-0.007892	-5.142598	-0.495926
H	1.014182	-5.495481	-0.624117
H	-0.448379	-5.605138	0.386909
H	-0.600989	-5.398693	-1.373184

$^{1}[Fe^{II}(Py5OH)H_2O]^{2+}$

59

Fe	0.000063	-0.003350	-0.745456
O	3.496590	0.000017	2.016664
H	4.334173	0.011034	1.540644
O	-0.000028	0.048486	-2.799542
N	-0.000212	-0.006508	1.253860
N	-1.513852	1.351471	-0.734152
O	-3.497201	-0.000129	2.015885
H	-4.334683	0.010941	1.539686
N	-1.515880	-1.355161	-0.735477
N	1.513912	1.351391	-0.733924
N	1.516083	-1.354978	-0.735344
C	1.625945	-2.360302	-1.616747
H	0.909339	-2.379892	-2.416584
C	-1.625081	-2.360927	-1.616465
H	-0.908138	-2.380682	-2.415986
C	-2.605759	-3.332798	-1.545474
H	-2.634585	-4.116798	-2.286681
C	-3.521483	-3.272201	-0.508562
H	-4.287700	-4.025556	-0.396521
C	-3.450632	-2.208081	0.378237
H	-4.157701	-2.126780	1.187013
C	-2.458279	-1.252136	0.218573
C	-2.422800	-0.000872	1.112601
C	-1.151765	-0.009084	1.943372
C	-1.193958	-0.018030	3.327553
H	-2.144216	-0.020509	3.832772
C	-0.000539	-0.023476	4.029656
H	-0.000666	-0.031170	5.110034
C	1.193047	-0.018006	3.327836
H	2.143182	-0.020453	3.833284
C	1.151176	-0.009065	1.943644
C	2.422382	-0.000796	1.113162
C	1.622230	2.355238	-1.617419
H	0.916990	2.358573	-2.427463
C	2.591764	3.337640	-1.535289
H	2.621454	4.121101	-2.277080
C	3.496172	3.288322	-0.487766
H	4.252027	4.050819	-0.367250
C	3.429662	2.222485	0.397514
H	4.130988	2.147552	1.211971
C	2.458199	-1.251980	0.219003
C	2.606897	-3.331904	-1.545810
H	2.636261	-4.115557	-2.287360
C	3.522180	-3.271507	-0.508498
H	4.288550	-4.024707	-0.396459
C	3.450727	-2.207738	0.378678
H	4.157493	-2.126563	1.187734
C	-2.449436	1.256211	0.226767
C	-3.429990	2.222370	0.396778
H	-4.131584	2.147339	1.210995
C	-3.496238	3.288330	-0.488371
H	-4.252168	4.050776	-0.367999
C	-2.591459	3.337845	-1.535561
H	-2.620893	4.121440	-2.277219
C	-1.621882	2.355471	-1.617511
H	-0.916360	2.358941	-2.427304
C	2.449195	1.256276	0.227301
H	0.779165	-0.080227	-3.350417
H	-0.779450	-0.080703	-3.349977

$^{5}[Fe^{II}(Py5OH)H_{2}O]^{2+}$

59

Fe	0.000452	-0.013365	-0.956880
O	3.506662	-0.007005	2.017703
H	4.355958	0.029341	1.564077
O	-0.003870	0.261364	-3.110900
N	0.001485	-0.029789	1.232662
N	-1.650575	1.412507	-0.779443
O	-3.502054	-0.019364	2.024230
H	-4.352292	0.009662	1.571842
N	-1.670923	-1.427213	-0.806666
N	1.670953	1.384928	-0.814549
N	1.645690	-1.460875	-0.771957
C	1.744304	-2.536457	-1.564259
H	1.017341	-2.618428	-2.356634
C	-1.790237	-2.480096	-1.626607
H	-1.086118	-2.533446	-2.442246
C	-2.757524	-3.455063	-1.467957
H	-2.810901	-4.281806	-2.159841
C	-3.629239	-3.340418	-0.396731
H	-4.385152	-4.091129	-0.215772
C	-3.525672	-2.241535	0.444899
H	-4.191286	-2.141926	1.285718
C	-2.548228	-1.284316	0.197996
C	-2.455431	-0.017864	1.083668
C	-1.156648	-0.031735	1.906594
C	-1.194755	-0.033686	3.292856
H	-2.138866	-0.035561	3.808847
C	0.004263	-0.032006	3.987230
H	0.005392	-0.035151	5.067910
C	1.201873	-0.024793	3.290419
H	2.147002	-0.019785	3.804513
C	1.161033	-0.025068	1.904264
C	2.458458	-0.015443	1.078879
C	1.764671	2.452514	-1.618480
H	1.083110	2.479019	-2.453707
C	2.681094	3.468056	-1.416482
H	2.717336	4.306240	-2.095690
C	3.525623	3.378404	-0.321162
H	4.240873	4.159910	-0.108296
C	3.448676	2.263453	0.502252
H	4.094327	2.180504	1.360467
C	2.544612	-1.294053	0.210084
C	2.717782	-3.505530	-1.404903
H	2.753526	-4.351172	-2.074737
C	3.618527	-3.360150	-0.362076
H	4.382282	-4.103279	-0.182720
C	3.531455	-2.241704	0.455333
H	4.216114	-2.121018	1.277806
C	-2.518179	1.275191	0.233390
C	-3.449007	2.264760	0.527223
H	-4.107666	2.164822	1.373657
C	-3.514789	3.395098	-0.275941
H	-4.235301	4.170971	-0.060215
C	-2.650844	3.508281	-1.353782
H	-2.675433	4.360728	-2.015523
C	-1.730544	2.497180	-1.561199
H	-1.033986	2.543462	-2.382919
C	2.524374	1.266508	0.212488
H	0.762411	0.046818	-3.655443
H	-0.786635	0.094678	-3.648792

$^{2}[Fe^{III}(Py5OH)H_2O]^{3+}$

59

Fe	-0.000143	0.000088	-0.746664
O	-3.495491	0.000025	2.019552
H	-4.341381	0.000335	1.556946
O	-0.000432	0.000199	-2.715533
N	0.000286	-0.000349	1.244530
N	1.520813	-1.333773	-0.738274
O	3.496404	-0.000171	2.018027
H	4.342090	-0.000265	1.555041
N	1.510243	1.346602	-0.726458
N	-1.510684	-1.346430	-0.725967
N	-1.520963	1.333895	-0.737474
C	-1.615261	2.331545	-1.631859
H	-0.912126	2.335191	-2.441840
C	1.593221	2.357121	-1.607277
H	0.881607	2.371531	-2.409456
C	2.557419	3.342060	-1.528889
H	2.570723	4.128727	-2.267025
C	3.474297	3.291883	-0.492982
H	4.226534	4.058238	-0.377799
C	3.425628	2.222581	0.388905
H	4.135228	2.146063	1.195723
C	2.452437	1.252300	0.227735
C	2.433695	0.000324	1.114298
C	1.157446	-0.001814	1.931461
C	1.194715	-0.002785	3.312326
H	2.145603	-0.004320	3.815890
C	0.000880	-0.000964	4.013073
H	0.001111	-0.001224	5.093132
C	-1.193254	0.001271	3.312839
H	-2.143925	0.002675	3.816815
C	-1.156578	0.000976	1.931956
C	-2.433187	-0.000495	1.115349
C	-1.594060	-2.356721	-1.607002
H	-0.882626	-2.371113	-2.409342
C	-2.558461	-3.341473	-1.528637
H	-2.572085	-4.127960	-2.266960
C	-3.475124	-3.291344	-0.492542
H	-4.227525	-4.057545	-0.377411
C	-3.426008	-2.222286	0.389613
H	-4.135380	-2.145821	1.196637
C	-2.457547	1.246205	0.222403
C	-2.582117	3.314160	-1.558193
H	-2.604865	4.090667	-2.306784
C	-3.489842	3.274845	-0.513638
H	-4.242861	4.040884	-0.401511
C	-3.432437	2.215512	0.379676
H	-4.136730	2.145541	1.191755
C	2.457917	-1.246147	0.221071
C	3.433024	-2.215347	0.377658
H	4.137770	-2.145397	1.189347
C	3.490031	-3.274557	-0.515823
H	4.243207	-4.040520	-0.404243
C	2.581674	-3.313848	-1.559829
H	2.604043	-4.090274	-2.308518
C	1.614678	-2.331309	-1.632829
H	0.911060	-2.334950	-2.442395
C	-2.452615	-1.252200	0.228463
H	-0.785645	-0.013644	-3.279755
H	0.784755	0.013912	-3.279796

[1][Fe^{II}(Py5OMe)H_2O]$^{2+}$

65

Fe	-0.000641	0.183966	-0.801964
O	3.576822	-0.343295	1.815578
C	3.721591	0.495210	2.978680
N	0.003436	-0.192290	1.120636
N	-1.516503	1.494310	-0.581349
O	-3.565823	-0.342559	1.830852
C	-3.705395	0.496632	2.994115
N	-1.554507	-1.176141	-1.039522
N	1.502557	1.507203	-0.565865
N	1.558004	-1.168429	-1.049189
C	1.661919	-1.987470	-2.105960
H	0.900471	-1.901977	-2.858562
C	-1.660227	-2.001652	-2.090883
H	-0.898980	-1.922280	-2.844371
C	-2.691549	-2.909758	-2.248426
H	-2.710698	-3.546609	-3.119668
C	-3.677083	-2.971437	-1.276227
H	-4.494987	-3.672893	-1.357257
C	-3.606720	-2.096550	-0.201692
H	-4.362126	-2.083003	0.565744
C	-2.544242	-1.211231	-0.125925
C	-2.451689	-0.166573	0.995752
C	-1.152492	-0.468468	1.743644
C	-1.183484	-1.145555	2.953110
H	-2.121978	-1.432909	3.390443
C	0.009113	-1.501654	3.555153
H	0.011219	-2.059165	4.480365
C	1.199025	-1.147334	2.946610
H	2.139574	-1.435771	3.378865
C	1.162620	-0.470117	1.737544
C	2.459378	-0.167083	0.984994
C	1.622301	2.615631	-1.313432
H	0.839589	2.804700	-2.020840
C	2.704513	3.471113	-1.246880
H	2.730571	4.347781	-1.875923
C	3.743779	3.150303	-0.391224
H	4.623636	3.773397	-0.321888
C	3.649993	1.988115	0.357233
H	4.471790	1.686877	0.979742
C	2.549878	-1.208033	-0.138799
C	2.692401	-2.895218	-2.270132
H	2.709783	-3.527172	-3.144976
C	3.679601	-2.962865	-1.299906
H	4.496698	-3.664643	-1.386096
C	3.612155	-2.093108	-0.221064
H	4.369468	-2.083086	0.544533
C	-2.511734	1.194292	0.272735
C	-3.650515	1.984520	0.367077
H	-4.460857	1.693721	1.009025
C	-3.761481	3.131342	-0.402496
H	-4.640979	3.754333	-0.327851
C	-2.742807	3.433616	-1.289079
H	-2.786701	4.293231	-1.940279
C	-1.658392	2.581176	-1.356521
H	-0.894105	2.751028	-2.089151
C	2.511335	1.197211	0.268604
H	3.655471	-0.118088	3.874404
H	2.963835	1.275274	3.024393
H	4.706982	0.954345	2.943239
H	-3.632382	-0.115252	3.890253
H	-4.692194	0.953166	2.964236
H	-2.949266	1.278593	3.034325
O	0.009097	0.559575	-2.824397
H	-0.758231	0.461028	-3.397826
H	0.798641	0.499605	-3.372690

$^{5}[Fe^{II}(Py5OMe)H_2O]^{2+}$

65

Fe	-0.001462	0.231663	-0.984013
O	3.547675	-0.386660	1.883446
C	3.518175	0.357520	3.114537
N	-0.003458	-0.170887	1.121415
N	-1.634904	1.588835	-0.570287
O	-3.552779	-0.308851	1.894698
C	-3.496139	0.461921	3.108376
N	-1.759253	-1.237870	-1.088541
N	1.610374	1.626932	-0.469114
N	1.751712	-1.193570	-1.132147
C	1.891971	-2.023733	-2.173292
H	1.114058	-1.989848	-2.921318
C	-1.899699	-2.109505	-2.094797
H	-1.114540	-2.117645	-2.836008
C	-2.985482	-2.962722	-2.205476
H	-3.048607	-3.649730	-3.035958
C	-3.970463	-2.906853	-1.230136
H	-4.833009	-3.556505	-1.278997
C	-3.838162	-2.001717	-0.184761
H	-4.581526	-1.926473	0.591326
C	-2.718754	-1.184889	-0.151673
C	-2.497088	-0.158613	0.978914
C	-1.167621	-0.576990	1.643917
C	-1.209043	-1.507545	2.674300
H	-2.154345	-1.871596	3.039401
C	-0.017602	-2.000895	3.177158
H	-0.023751	-2.749962	3.956020
C	1.181967	-1.543371	2.658570
H	2.120544	-1.936096	3.011079
C	1.155663	-0.607036	1.634240
C	2.490655	-0.196428	0.975637
C	1.732426	2.811325	-1.083317
H	0.935711	3.083072	-1.755243
C	2.821615	3.644730	-0.910020
H	2.869023	4.589651	-1.430027
C	3.843008	3.215662	-0.076771
H	4.726074	3.820254	0.074349
C	3.727541	1.986262	0.554513
H	4.528099	1.623452	1.174657
C	2.703499	-1.187665	-0.186165
C	2.969853	-2.882171	-2.310828
H	3.034069	-3.533534	-3.169451
C	3.946504	-2.877318	-1.325397
H	4.802915	-3.533216	-1.394259
C	3.814710	-2.014763	-0.244689
H	4.553213	-1.978444	0.538718
C	-2.584436	1.234062	0.307552
C	-3.716392	2.022051	0.495097
H	-4.497944	1.706024	1.162942
C	-3.849385	3.208193	-0.211082
H	-4.723701	3.827105	-0.067305
C	-2.861659	3.571603	-1.113436
H	-2.929883	4.474991	-1.700500
C	-1.779929	2.725090	-1.265632
H	-1.010162	2.938893	-1.989258
C	2.583244	1.219166	0.357455
H	3.122161	-0.253709	3.923229
H	2.918990	1.263959	3.034663
H	4.546822	0.627037	3.341581
H	-3.084712	-0.131589	3.922673
H	-4.519288	0.739298	3.349991
H	-2.896173	1.364202	2.994628
O	0.161965	0.818739	-3.054723
H	-0.549090	0.766826	-3.703441
H	0.993009	0.808223	-3.542577

$^{2}[Fe^{III}(Py5OMe)H_2O]^{3+}$

65

Fe	-0.000265	0.153523	-0.806679
O	3.578571	-0.292168	1.820740
C	3.730526	0.568743	2.973121
N	0.003151	-0.167042	1.115484
N	-1.517190	1.454480	-0.632887
O	-3.568589	-0.291744	1.834512
C	-3.718686	0.571489	2.985432
N	-1.549643	-1.202169	-0.996686
N	1.493103	1.477367	-0.603785
N	1.564095	-1.182488	-1.016224
C	1.658314	-2.016387	-2.064154
H	0.902104	-1.943559	-2.821198
C	-1.634175	-2.056820	-2.028174
H	-0.870911	-1.999256	-2.779273
C	-2.662436	-2.969131	-2.165899
H	-2.669021	-3.629808	-3.018835
C	-3.658607	-3.002949	-1.204297
H	-4.474353	-3.707493	-1.274834
C	-3.603629	-2.097604	-0.153979
H	-4.366682	-2.061081	0.605018
C	-2.546196	-1.210442	-0.090893
C	-2.461444	-0.138491	1.002432
C	-1.158660	-0.422659	1.743955
C	-1.184600	-1.061765	2.970065
H	-2.123548	-1.331841	3.416519
C	0.008745	-1.405160	3.577666
H	0.010625	-1.938984	4.516433
C	1.199636	-1.069363	2.960860
H	2.140580	-1.344796	3.399972
C	1.168366	-0.428190	1.736258
C	2.468946	-0.139246	0.991902
C	1.586789	2.568789	-1.382110
H	0.809912	2.733395	-2.099923
C	2.649676	3.445445	-1.326288
H	2.659789	4.306008	-1.976750
C	3.688274	3.167589	-0.455685
H	4.552844	3.812029	-0.395752
C	3.617310	2.022075	0.320227
H	4.440421	1.752741	0.955233
C	2.557622	-1.201595	-0.108120
C	2.689224	-2.923943	-2.211625
H	2.703049	-3.568489	-3.076728
C	3.678868	-2.974139	-1.243817
H	4.495580	-3.676834	-1.321205
C	3.617399	-2.085492	-0.179819
H	4.377599	-2.058173	0.582405
C	-2.505858	1.197570	0.242534
C	-3.623584	2.013639	0.322217
H	-4.430570	1.760201	0.983547
C	-3.720391	3.134288	-0.486392
H	-4.585433	3.777702	-0.422487
C	-2.711362	3.383434	-1.399431
H	-2.748097	4.217073	-2.083166
C	-1.644063	2.512440	-1.452510
H	-0.893313	2.649763	-2.203827
C	2.501085	1.204070	0.244549
H	3.666058	-0.032244	3.876255
H	2.975802	1.351841	3.006740
H	4.718401	1.018890	2.922337
H	-3.648433	-0.026574	3.890064
H	-4.708485	1.017764	2.937789
H	-2.966516	1.357233	3.013775
O	0.007510	0.391495	-2.755984
H	-0.775362	0.443442	-3.319282
H	0.797764	0.490569	-3.302762

$^{6}[Fe^{III}(Py5OMe)H_2O]^{3+}$

65

Fe	0.000472	0.163206	-0.914274
O	3.538073	-0.226711	1.903945
C	3.595527	0.650472	3.051510
N	-0.023921	-0.127796	1.136378
N	-1.520337	1.595299	-0.549669
O	-3.609310	-0.282116	1.798231
C	-3.724203	0.562890	2.965229
N	-1.672161	-1.191355	-1.090196
N	1.623856	1.478986	-0.707270
N	1.627712	-1.299711	-0.954168
C	1.713183	-2.229643	-1.916164
H	0.930903	-2.238806	-2.655207
C	-1.765765	-2.051463	-2.116430
H	-0.999125	-1.990502	-2.869444
C	-2.800463	-2.958414	-2.233038
H	-2.829370	-3.628998	-3.077605
C	-3.781716	-2.972194	-1.253562
H	-4.605564	-3.668720	-1.308770
C	-3.705039	-2.067878	-0.201204
H	-4.456814	-2.034639	0.568712
C	-2.639950	-1.188776	-0.154834
C	-2.508993	-0.129416	0.953446
C	-1.200508	-0.444826	1.705516
C	-1.242188	-1.158993	2.889625
H	-2.183487	-1.475724	3.300927
C	-0.051906	-1.509559	3.503858
H	-0.061537	-2.096196	4.410525
C	1.150936	-1.122208	2.939163
H	2.083321	-1.414879	3.386968
C	1.136385	-0.413420	1.749605
C	2.461141	-0.111212	1.024376
C	1.797242	2.512665	-1.548822
H	1.062876	2.634362	-2.326513
C	2.877183	3.364776	-1.468291
H	2.968497	4.182872	-2.165272
C	3.833682	3.114205	-0.498220
H	4.706652	3.743598	-0.407346
C	3.681584	2.017784	0.338900
H	4.454047	1.778660	1.045377
C	2.603171	-1.228949	-0.029242
C	2.756087	-3.134365	-1.982726
H	2.778770	-3.864981	-2.776187
C	3.750856	-3.069935	-1.019549
H	4.581174	-3.760696	-1.037679
C	3.676511	-2.098081	-0.028575
H	4.434392	-2.008329	0.730997
C	-2.550235	1.242792	0.242143
C	-3.680259	2.039992	0.328328
H	-4.526992	1.718223	0.906258
C	-3.725192	3.246271	-0.355979
H	-4.601925	3.873274	-0.287591
C	-2.641379	3.623755	-1.131445
H	-2.626423	4.551119	-1.682158
C	-1.567898	2.762014	-1.213758
H	-0.728118	3.002568	-1.839201
C	2.556366	1.215348	0.227749
H	3.394283	0.083910	3.956647
H	2.883800	1.470474	2.978631
H	4.605078	1.049071	3.102403
H	-3.624004	-0.047406	3.858697
H	-4.714593	1.009871	2.952537
H	-2.972983	1.349858	2.981510
O	0.031029	0.346232	-2.991911
H	-0.732093	0.530497	-3.556251
H	0.817618	0.339157	-3.553128

[1][Fe^{II}(Py5OH)OH]$^{+}$

58

Fe	-0.001841	0.000020	-0.772278
O	3.530665	-0.000017	1.968834
H	4.357074	-0.000104	1.473751
O	-0.063828	-0.000228	-2.723116
N	0.020209	0.000049	1.257058
N	-1.521705	1.329398	-0.733980
O	-3.469659	0.000046	2.052035
H	-4.306579	-0.000003	1.575093
N	-1.521593	-1.329487	-0.733841
N	1.502069	1.347794	-0.750355
N	1.502119	-1.347658	-0.750438
C	1.585556	-2.351630	-1.635592
H	0.859049	-2.333705	-2.426995
C	-1.657667	-2.291691	-1.656110
H	-0.977598	-2.226547	-2.486377
C	-2.633243	-3.270905	-1.583554
H	-2.691129	-4.026633	-2.352595
C	-3.514102	-3.252185	-0.513830
H	-4.272775	-4.013822	-0.403297
C	-3.426892	-2.213230	0.402810
H	-4.117931	-2.154161	1.227845
C	-2.440138	-1.250533	0.243619
C	-2.398112	0.000033	1.141266
C	-1.121319	0.000068	1.962325
C	-1.147643	0.000082	3.347720
H	-2.091551	0.000099	3.865289
C	0.054938	0.000065	4.035522
H	0.068539	0.000073	5.116029
C	1.240040	0.000030	3.317802
H	2.196467	0.000006	3.811769
C	1.179478	0.000023	1.933588
C	2.438764	-0.000011	1.082313
C	1.585456	2.351938	-1.635324
H	0.858861	2.334287	-2.426644
C	2.553606	3.338399	-1.570599
H	2.565329	4.125020	-2.310088
C	3.483779	3.288568	-0.544806
H	4.241471	4.052108	-0.441526
C	3.439632	2.221751	0.341619
H	4.160250	2.146956	1.139448
C	2.455318	-1.254699	0.192652
C	2.553674	-3.338129	-1.570926
H	2.565456	-4.124632	-2.310540
C	3.483744	-3.288495	-0.545025
H	4.241384	-4.052092	-0.441781
C	3.439580	-2.221797	0.341546
H	4.160147	-2.147137	1.139436
C	-2.440209	1.250517	0.243521
C	-3.427025	2.213166	0.402618
H	-4.118025	2.154163	1.227691
C	-3.514358	3.251972	-0.514179
H	-4.273081	4.013571	-0.403727
C	-2.633585	3.270563	-1.583976
H	-2.691599	4.026136	-2.353159
C	-1.657940	2.291410	-1.656427
H	-0.977978	2.226111	-2.486768
C	2.455326	1.254706	0.192677
H	0.827022	0.000798	-3.082249

$^{5}[Fe^{II}(Py5OH)OH]^{+}$

58

Fe	0.008861	-0.000799	-1.135642
O	-3.512282	0.001029	2.022938
H	-4.359240	0.001669	1.564287
O	0.029363	-0.002556	-3.050259
N	-0.005558	0.000087	1.232096
N	1.671770	-1.411009	-0.783076
O	3.495783	0.000613	2.045551
H	4.345298	0.001295	1.591550
N	1.671443	1.410479	-0.783766
N	-1.659042	-1.424967	-0.780237
N	-1.658467	1.424402	-0.781211
C	-1.754046	2.488010	-1.587231
H	-1.043538	2.523959	-2.398909
C	1.784276	2.459764	-1.606061
H	1.091902	2.477593	-2.433890
C	2.731771	3.452921	-1.430847
H	2.784588	4.280880	-2.121887
C	3.588865	3.353948	-0.344866
H	4.328340	4.118355	-0.152237
C	3.494516	2.251582	0.493800
H	4.152566	2.160384	1.342034
C	2.535406	1.278244	0.229443
C	2.449939	0.000221	1.098718
C	1.146341	-0.000255	1.912536
C	1.184474	-0.000988	3.301234
H	2.127656	-0.001267	3.819138
C	-0.014874	-0.001314	3.994206
H	-0.018627	-0.001896	5.075165
C	-1.209528	-0.000846	3.293049
H	-2.156211	-0.001011	3.804498
C	-1.161990	-0.000124	1.904730
C	-2.460621	0.000436	1.082316
C	-1.755136	-2.488812	-1.585881
H	-1.044035	-2.525857	-2.396987
C	-2.705559	-3.479889	-1.414717
H	-2.742888	-4.319388	-2.092711
C	-3.585163	-3.363155	-0.348981
H	-4.329667	-4.123599	-0.160076
C	-3.504667	-2.249579	0.476037
H	-4.177395	-2.148130	1.311370
C	-2.538356	1.281272	0.216660
C	-2.703213	3.480228	-1.415733
H	-2.740144	4.319484	-2.094049
C	-3.582051	3.364947	-0.349209
H	-4.325483	4.126351	-0.159957
C	-3.502206	2.251573	0.476142
H	-4.174442	2.151191	1.312004
C	2.536325	-1.277744	0.229487
C	3.496737	-2.249964	0.493205
H	4.155323	-2.157930	1.340930
C	3.591664	-3.352317	-0.345408
H	4.332186	-4.115848	-0.153308
C	2.733776	-3.452408	-1.430665
H	2.786948	-4.280421	-2.121615
C	1.785057	-2.460321	-1.605300
H	1.092073	-2.478985	-2.432597
C	-2.539472	-1.280512	0.216955
H	-0.813109	-0.001201	-3.510240

$^{2}[Fe^{III}(Py5OH)OH]^{2+}$

58

Fe	0.001864	-0.000056	-0.805769
O	-3.550144	0.000034	1.934869
H	-4.378488	0.000215	1.441727
O	0.071549	-0.000056	-2.611422
N	-0.035655	-0.000042	1.258355
N	1.533775	-1.320685	-0.737606
O	3.442785	0.000063	2.075888
H	4.289308	0.000121	1.615227
N	1.533638	1.320703	-0.737577
N	-1.503883	-1.347494	-0.766808
N	-1.503899	1.347356	-0.766899
C	-1.559674	2.365928	-1.638678
H	-0.827680	2.364474	-2.423993
C	1.678165	2.277258	-1.664922
H	1.018039	2.218004	-2.510318
C	2.646753	3.259487	-1.576626
H	2.715809	4.009527	-2.349422
C	3.504033	3.250319	-0.488796
H	4.256185	4.016191	-0.367164
C	3.405791	2.216219	0.431454
H	4.081068	2.162581	1.269373
C	2.430190	1.247711	0.259568
C	2.385363	0.000020	1.159228
C	1.102140	-0.000061	1.966721
C	1.115848	-0.000135	3.350514
H	2.054796	-0.000153	3.876336
C	-0.093854	-0.000176	4.024614
H	-0.116782	-0.000235	5.104717
C	-1.274625	-0.000129	3.299903
H	-2.234692	-0.000141	3.786069
C	-1.203478	-0.000062	1.918490
C	-2.457426	0.000003	1.060229
C	-1.559668	-2.366119	-1.638525
H	-0.827583	-2.364817	-2.423748
C	-2.514156	-3.362303	-1.565623
H	-2.507779	-4.158596	-2.293849
C	-3.451553	-3.309020	-0.547495
H	-4.199840	-4.080603	-0.439268
C	-3.428045	-2.232118	0.326377
H	-4.153306	-2.156401	1.119392
C	-2.458217	1.254314	0.173576
C	-2.514024	3.362237	-1.565698
H	-2.507653	4.158486	-2.293973
C	-3.451272	3.309145	-0.547420
H	-4.199421	4.080849	-0.439110
C	-3.427798	2.232277	0.326498
H	-4.152964	2.156697	1.119613
C	2.430320	-1.247640	0.259540
C	3.406031	-2.216044	0.431389
H	4.081297	-2.162366	1.269315
C	3.504399	-3.250087	-0.488910
H	4.256642	-4.015875	-0.367315
C	2.647142	-3.259283	-1.576757
H	2.716309	-4.009260	-2.349605
C	1.678443	-2.277162	-1.665012
H	1.018360	-2.217909	-2.510439
C	-2.458304	-1.254299	0.173552
H	-0.806585	-0.000192	-3.007664

$^{6}[Fe^{III}(Py5OH)OH]^{2+}$

58

Fe	0.014069	0.000020	-1.037748
O	-3.531665	-0.000000	1.983966
H	-4.375016	-0.000210	1.517524
O	0.023992	-0.000048	-2.858258
N	-0.022193	0.000099	1.236248
N	1.634040	-1.386800	-0.773448
O	3.470517	-0.000092	2.058699
H	4.323796	-0.000114	1.610763
N	1.634189	1.386674	-0.773497
N	-1.607657	-1.408890	-0.779740
N	-1.607529	1.409049	-0.779612
C	-1.679031	2.463467	-1.604474
H	-0.963047	2.489003	-2.408716
C	1.753317	2.409662	-1.631202
H	1.079926	2.403252	-2.472401
C	2.699922	3.403565	-1.472320
H	2.759485	4.209815	-2.187070
C	3.550132	3.330510	-0.380249
H	4.290420	4.097675	-0.204801
C	3.457402	2.245902	0.482525
H	4.119088	2.167213	1.328880
C	2.500828	1.270332	0.243889
C	2.427218	-0.000066	1.120781
C	1.125814	0.000053	1.927663
C	1.153040	-0.000035	3.313506
H	2.094169	-0.000112	3.834956
C	-0.050578	-0.000046	3.998733
H	-0.061550	-0.000109	5.079182
C	-1.240018	-0.000016	3.289072
H	-2.191960	-0.000053	3.790555
C	-1.183701	0.000034	1.904393
C	-2.467546	0.000055	1.069319
C	-1.679391	-2.463180	-1.604748
H	-0.963487	-2.488703	-2.409060
C	-2.624633	-3.460081	-1.458715
H	-2.642110	-4.290536	-2.147568
C	-3.527207	-3.358404	-0.412109
H	-4.271283	-4.124358	-0.247785
C	-3.476430	-2.249983	0.422465
H	-4.172590	-2.154811	1.238775
C	-2.514567	1.275239	0.199561
C	-2.624147	3.460479	-1.458392
H	-2.641423	4.291043	-2.147119
C	-3.526828	3.358790	-0.411881
H	-4.270788	4.124844	-0.247504
C	-3.476267	2.250262	0.422565
H	-4.172449	2.155131	1.238855
C	2.500659	-1.270528	0.243965
C	3.457055	-2.246252	0.482702
H	4.118704	-2.167633	1.329090
C	3.549646	-3.330923	-0.380008
H	4.289795	-4.098205	-0.204480
C	2.699490	-3.403888	-1.472128
H	2.758967	-4.210179	-2.186840
C	1.753055	-2.409842	-1.631104
H	1.079718	-2.403359	-2.472346
C	-2.514589	-1.275094	0.199534
H	-0.728720	0.000017	-3.456423

$^{5}[Fe^{II}(Py5OMe)OH]^{+}$

64

Fe	-0.010417	0.212655	1.140606
O	-3.546943	-0.268037	-1.920245
C	-3.476409	0.520982	-3.119091
N	-0.002812	-0.106014	-1.126973
N	1.627261	1.597716	0.579778
O	3.540679	-0.253410	-1.925551
C	3.466023	0.543614	-3.118527
N	1.760454	-1.268998	1.032736
N	-1.641839	1.585443	0.589617
N	-1.766132	-1.244745	1.052324
C	-1.914223	-2.105317	2.064395
H	-1.129899	-2.090486	2.806276
C	1.909509	-2.145135	2.031049
H	1.115754	-2.159013	2.763017
C	3.014440	-2.974778	2.141505
H	3.087819	-3.671436	2.963267
C	4.009283	-2.880905	1.177917
H	4.886747	-3.510274	1.228595
C	3.869354	-1.962352	0.145086
H	4.622365	-1.854011	-0.617780
C	2.727450	-1.175325	0.110642
C	2.490149	-0.124409	-0.992187
C	1.154323	-0.524643	-1.652008
C	1.192812	-1.450915	-2.688228
H	2.137288	-1.821206	-3.049752
C	-0.000121	-1.925374	-3.204320
H	0.001093	-2.665286	-3.992014
C	-1.194638	-1.456753	-2.686412
H	-2.137741	-1.831934	-3.046302
C	-1.158714	-0.529462	-1.651142
C	-2.494898	-0.131253	-0.990294
C	-1.787843	2.706696	1.305595
H	-1.019963	2.898163	2.037988
C	-2.867402	3.559337	1.164706
H	-2.935367	4.453197	1.766480
C	-3.854759	3.213510	0.254288
H	-4.728282	3.835850	0.120501
C	-3.721399	2.039683	-0.471986
H	-4.501785	1.733535	-1.146116
C	-2.725741	-1.175903	0.119924
C	-3.009424	-2.947424	2.175804
H	-3.083726	-3.630434	3.008859
C	-3.993853	-2.883978	1.199076
H	-4.862636	-3.525283	1.249811
C	-3.856026	-1.979085	0.153886
H	-4.602624	-1.892327	-0.617995
C	2.582288	1.253100	-0.293037
C	3.721563	2.038005	-0.459304
H	4.507911	1.724473	-1.123235
C	3.851503	3.215451	0.261486
H	4.729711	3.832769	0.135304
C	2.852782	3.572889	1.154798
H	2.915196	4.471903	1.749463
C	1.768234	2.725155	1.287296
H	0.989570	2.929595	2.004670
C	-2.588334	1.248487	-0.295709
H	-3.053370	-0.058793	-3.937771
H	-2.879441	1.422098	-2.983710
H	-4.496204	0.802593	-3.370623
H	3.023468	-0.024024	-3.935461
H	4.486931	0.810953	-3.380648
H	2.885103	1.453126	-2.969778
O	-0.012955	0.375226	3.040200
H	0.832585	0.479795	3.482314

$^{2}[Fe^{III}(Py5OMe)OH]^{2+}$

64

Fe	0.002686	0.163439	-0.870062
O	3.521966	-0.304075	1.883392
C	3.641638	0.538454	3.048560
N	-0.031631	-0.156844	1.124465
N	-1.481210	1.493085	-0.626220
O	-3.620542	-0.283055	1.747587
C	-3.778908	0.569585	2.901443
N	-1.548265	-1.187852	-1.046989
N	1.538153	1.439094	-0.646161
N	1.574406	-1.170683	-1.015730
C	1.714358	-1.960818	-2.088019
H	1.000708	-1.816181	-2.877274
C	-1.601308	-2.050412	-2.072462
H	-0.817628	-1.975363	-2.801778
C	-2.617971	-2.974666	-2.223928
H	-2.602327	-3.644793	-3.069632
C	-3.636339	-3.007019	-1.284675
H	-4.445842	-3.717665	-1.368426
C	-3.610945	-2.095106	-0.238979
H	-4.390116	-2.061471	0.503552
C	-2.558537	-1.200543	-0.159253
C	-2.489381	-0.133164	0.938364
C	-1.205052	-0.429544	1.716275
C	-1.266427	-1.093790	2.929561
H	-2.215808	-1.374199	3.347316
C	-0.087653	-1.447350	3.561079
H	-0.109658	-1.997571	4.490332
C	1.118766	-1.104138	2.979497
H	2.047601	-1.393236	3.435766
C	1.111931	-0.437789	1.764877
C	2.420213	-0.146099	1.036133
C	1.713631	2.456242	-1.502294
H	1.011044	2.519190	-2.311049
C	2.776316	3.333265	-1.417623
H	2.859805	4.138879	-2.130685
C	3.727803	3.121420	-0.435491
H	4.586031	3.770799	-0.341710
C	3.592741	2.022404	0.398658
H	4.368592	1.791126	1.103719
C	2.529714	-1.209275	-0.069322
C	2.744377	-2.875277	-2.213363
H	2.801253	-3.492010	-3.097153
C	3.685033	-2.963885	-1.199795
H	4.498991	-3.672019	-1.258905
C	3.587055	-2.099882	-0.117521
H	4.319590	-2.095073	0.671802
C	-2.505597	1.215035	0.197956
C	-3.616878	2.043436	0.267843
H	-4.457647	1.770078	0.877718
C	-3.655724	3.208554	-0.480645
H	-4.515324	3.860507	-0.426305
C	-2.589066	3.500728	-1.312725
H	-2.572032	4.383046	-1.933633
C	-1.537253	2.609247	-1.369927
H	-0.731474	2.770794	-2.057329
C	2.482402	1.198560	0.280228
H	3.559732	-0.073279	3.943681
H	2.881825	1.317168	3.076604
H	4.627514	0.996759	3.029597
H	-3.735917	-0.037140	3.802681
H	-4.759144	1.036015	2.841074
H	-3.014578	1.342654	2.953511
O	0.056645	0.399033	-2.663899
H	-0.823385	0.461697	-3.050763

^{6}[Fe^{III}(Py5OMe)OH]$^{2+}$

64

Fe	0.016187	0.194582	-1.065797
O	3.534370	-0.281662	1.902666
C	3.539402	0.529212	3.095140
N	-0.018057	-0.130819	1.115381
N	-1.575868	1.574847	-0.601153
O	-3.585803	-0.262911	1.837043
C	-3.614278	0.558208	3.022020
N	-1.677526	-1.247968	-1.050614
N	1.619795	1.535837	-0.625393
N	1.701519	-1.221835	-1.050418
C	1.836914	-2.067625	-2.080225
H	1.086560	-2.002373	-2.851165
C	-1.771297	-2.136645	-2.048712
H	-0.982264	-2.117354	-2.782224
C	-2.827650	-3.022980	-2.158615
H	-2.855195	-3.725077	-2.977848
C	-3.834075	-2.974102	-1.205380
H	-4.677095	-3.648137	-1.259059
C	-3.754636	-2.039373	-0.180537
H	-4.520699	-1.960893	0.572399
C	-2.660399	-1.192959	-0.137155
C	-2.499155	-0.126497	0.961271
C	-1.183200	-0.484156	1.675653
C	-1.230423	-1.301464	2.795852
H	-2.175657	-1.636289	3.185398
C	-0.042446	-1.729406	3.361245
H	-0.051852	-2.394411	4.212653
C	1.157751	-1.312678	2.813576
H	2.093729	-1.656636	3.217112
C	1.135334	-0.492795	1.694340
C	2.466014	-0.138042	1.006570
C	1.771056	2.624060	-1.393631
H	1.036478	2.764322	-2.168246
C	2.831793	3.495978	-1.250497
H	2.906840	4.362934	-1.888638
C	3.795215	3.198663	-0.300014
H	4.656319	3.837536	-0.166324
C	3.665366	2.045026	0.460414
H	4.441074	1.771591	1.151855
C	2.649113	-1.196834	-0.098145
C	2.893279	-2.955931	-2.175344
H	2.957826	-3.623837	-3.020631
C	3.852619	-2.952853	-1.173660
H	4.691944	-3.632475	-1.212944
C	3.736623	-2.051537	-0.122000
H	4.472817	-2.001863	0.662547
C	-2.559046	1.244518	0.250036
C	-3.683704	2.050109	0.381045
H	-4.497790	1.748287	1.014718
C	-3.767080	3.234708	-0.335259
H	-4.636614	3.867754	-0.232193
C	-2.736874	3.577652	-1.196306
H	-2.762551	4.479672	-1.788099
C	-1.671586	2.707597	-1.312840
H	-0.881118	2.898826	-2.017227
C	2.552251	1.231983	0.291121
H	3.288931	-0.079376	3.961009
H	2.838127	1.359542	3.031349
H	4.547881	0.917209	3.216077
H	-3.370381	-0.040267	3.896822
H	-4.627525	0.938813	3.124459
H	-2.919817	1.394376	2.960738
O	0.005664	0.430559	-2.867907
H	-0.745017	0.529641	-3.460631

^{2}[Co^{II}(Py5OH)MeCN]$^{2+}$

62

Co	-0.000707	0.617019	0.002332
O	-3.491027	-2.236271	-0.006904
H	-4.312967	-1.732301	-0.006283
N	0.003910	-1.581565	-0.004677
N	1.505146	0.540850	-1.337027
O	3.501521	-2.221055	-0.006947
H	4.321342	-1.713659	-0.006348
N	1.505153	0.532105	1.341079
N	-1.505783	0.533294	-1.337726
N	-1.505808	0.524662	1.341649
C	-1.617859	1.390241	2.358541
H	-0.950078	2.232090	2.345035
C	1.614663	1.399369	2.356804
H	0.944096	2.239033	2.342335
C	2.544669	1.264418	3.370431
H	2.581771	1.996362	4.162725
C	3.401891	0.177297	3.337034
H	4.125538	0.014774	4.122689
C	3.333838	-0.692724	2.258877
H	4.001742	-1.535630	2.195135
C	2.396977	-0.472239	1.258909
C	2.393368	-1.358555	-0.004102
C	1.155102	-2.245338	-0.006920
C	1.210215	-3.630746	-0.011537
H	2.159111	-4.139933	-0.013292
C	0.009889	-4.328664	-0.013822
H	0.012247	-5.409385	-0.017410
C	-1.193472	-3.635987	-0.011515
H	-2.140144	-4.149301	-0.013253
C	-1.144395	-2.250361	-0.006902
C	-2.386576	-1.369014	-0.004085
C	-1.617635	1.405036	-2.349353
H	-0.949918	2.246838	-2.330479
C	-2.546343	1.271798	-3.364380
H	-2.585380	2.007492	-4.153099
C	-3.399696	0.181482	-3.337021
H	-4.122207	0.020253	-4.123986
C	-3.329007	-0.693823	-2.263331
H	-3.993603	-1.539698	-2.204667
C	-2.393829	-0.482985	1.259038
C	-2.546917	1.250894	3.372413
H	-2.586130	1.981757	4.165605
C	-3.400413	0.160889	3.338134
H	-4.123191	-0.004991	4.123887
C	-3.329541	-0.707849	2.259135
H	-3.994237	-1.553239	2.194903
C	2.396814	-0.464157	-1.261398
C	3.333224	-0.678583	-2.263083
H	4.000975	-1.522020	-2.205008
C	3.401116	0.198127	-3.335819
H	4.124455	0.040296	-4.122713
C	2.544133	1.285635	-3.362137
H	2.581109	2.022517	-4.149845
C	1.614510	1.414415	-2.347344
H	0.944089	2.254087	-2.327341
C	-2.393699	-0.474970	-1.261548
N	-0.007906	2.844438	0.009471
C	-0.017008	3.992034	0.011481
C	-0.029032	5.439368	0.013479
H	-0.908232	5.798048	0.546547
H	-0.056124	5.806158	-1.011589
H	0.867962	5.813013	0.505013

$^{4}[Co^{II}(Py5OH)MeCN]^{2+}$

62

Co	0.000047	-0.001398	-0.683535
O	3.503288	0.024106	2.264571
H	4.350376	0.015171	1.805552
N	-0.000283	0.021703	1.487378
N	-1.638540	1.397268	-0.558113
O	-3.504070	0.024226	2.263505
H	-4.351025	0.015507	1.804236
N	-1.634487	-1.402541	-0.528203
N	1.638080	1.397905	-0.556946
N	1.634997	-1.401940	-0.528291
C	1.731820	-2.462735	-1.338503
H	1.035010	-2.502892	-2.159975
C	-1.730701	-2.463732	-1.337964
H	-1.033280	-2.504319	-2.158890
C	-2.672578	-3.461359	-1.163999
H	-2.707934	-4.296073	-1.847461
C	-3.544682	-3.357166	-0.091780
H	-4.281264	-4.124449	0.099076
C	-3.468461	-2.244848	0.735151
H	-4.137633	-2.146566	1.573605
C	-2.513847	-1.268958	0.474012
C	-2.449792	0.016129	1.331721
C	-1.157626	0.030198	2.159580
C	-1.199132	0.048810	3.545747
H	-2.144271	0.055186	4.060015
C	-0.000700	0.058283	4.241564
H	-0.000864	0.072515	5.322191
C	1.197944	0.048696	3.546114
H	2.142926	0.054982	4.060672
C	1.156861	0.030120	2.159933
C	2.449286	0.016149	1.332474
C	1.739156	2.439013	-1.391792
H	1.034994	2.467717	-2.207387
C	2.693502	3.429790	-1.248541
H	2.732855	4.248209	-1.951213
C	3.573229	3.339976	-0.181365
H	4.320568	4.102407	-0.014289
C	3.490354	2.248824	0.672454
H	4.164567	2.164711	1.508341
C	2.513732	-1.268733	0.474511
C	2.673580	-3.460431	-1.164333
H	2.709440	-4.294822	-1.848162
C	3.544904	-3.356736	-0.091429
H	4.281319	-4.124129	0.099626
C	3.468151	-2.244752	0.735905
H	4.136767	-2.146811	1.574843
C	-2.523033	1.277928	0.441452
C	-3.490641	2.248742	0.671143
H	-4.164764	2.164992	1.507141
C	-3.573579	3.339540	-0.183122
H	-4.320834	4.102097	-0.016238
C	-2.694079	3.428801	-1.250535
H	-2.733570	4.246869	-1.953608
C	-1.739781	2.437936	-1.393493
H	-1.035831	2.466151	-2.209302
C	2.522664	1.278155	0.442498
N	0.000533	-0.022221	-2.796069
C	0.001232	-0.049337	-3.942069
C	0.001974	-0.084904	-5.386209
H	0.882256	-0.621106	-5.737696
H	0.020951	0.932904	-5.773647
H	-0.896411	-0.588775	-5.739654

$^{1}[Co^{III}(Py5OH)MeCN]^{3+}$

62

Co	-0.000513	-0.001522	-0.527013
O	3.484522	0.004868	2.236456
H	4.327226	0.006478	1.767584
N	-0.006307	0.002253	1.466093
N	-1.494662	1.332360	-0.529937
O	-3.499126	0.003436	2.211849
H	-4.338162	0.004801	1.736518
N	-1.493965	-1.335957	-0.524532
N	1.479244	1.349265	-0.496857
N	1.480391	-1.351158	-0.491751
C	1.565978	-2.364343	-1.368097
H	0.862351	-2.382293	-2.174893
C	-1.602820	-2.319668	-1.431174
H	-0.918008	-2.310781	-2.254857
C	-2.568247	-3.303814	-1.356917
H	-2.598537	-4.070704	-2.115089
C	-3.465928	-3.275494	-0.304192
H	-4.217993	-4.042401	-0.191721
C	-3.404543	-2.219342	0.591890
H	-4.107734	-2.148588	1.404839
C	-2.430657	-1.248531	0.435628
C	-2.423443	0.001781	1.324250
C	-1.159935	0.002827	2.151587
C	-1.204325	0.004173	3.533379
H	-2.158641	0.004511	4.030650
C	-0.014348	0.004861	4.238955
H	-0.017330	0.005571	5.318964
C	1.179835	0.004524	3.540113
H	2.131099	0.005074	4.043214
C	1.144254	0.003243	2.158233
C	2.415683	0.002736	1.340268
C	1.563009	2.360290	-1.375879
H	0.859464	2.374974	-2.182993
C	2.521350	3.350349	-1.291887
H	2.530040	4.138798	-2.028191
C	3.438868	3.300707	-0.257343
H	4.188533	4.069089	-0.139115
C	3.394784	2.226223	0.617175
H	4.106434	2.146083	1.421745
C	2.425460	-1.250544	0.458393
C	2.526528	-3.352070	-1.282332
H	2.536707	-4.142096	-2.016935
C	3.444355	-3.297918	-0.248321
H	4.195949	-4.064215	-0.128736
C	3.398156	-2.221413	0.623663
H	4.109976	-2.137477	1.427628
C	-2.430410	1.249097	0.431500
C	-3.402798	2.221708	0.586201
H	-4.104788	2.154674	1.400561
C	-3.463955	3.275146	-0.313048
H	-4.214481	4.043716	-0.201741
C	-2.567885	3.298645	-1.367281
H	-2.598294	4.063044	-2.127962
C	-1.603839	2.313031	-1.439799
H	-0.920388	2.300504	-2.264555
C	2.424050	1.253016	0.454025
N	0.037648	-0.004827	-2.435748
C	0.098864	-0.004349	-3.577931
C	0.179009	-0.008576	-5.015894
H	1.035569	-0.605799	-5.326292
H	0.298566	1.014642	-5.370707
H	-0.735611	-0.434270	-5.426870

$^{1}[Fe^{II}(Py5OMe)MeCN]^{2+}$

68

Fe	-0.000110	0.652756	0.024753
O	-3.565725	-2.056399	-0.200604
C	-3.688206	-2.957248	-1.318401
N	0.001089	-1.329276	-0.149650
N	1.514470	0.764191	-1.317985
O	3.568650	-2.052197	-0.200584
C	3.692038	-2.953159	-1.318186
N	1.563296	0.476638	1.399206
N	-1.513901	0.761221	-1.319151
N	-1.563817	0.475340	1.398548
C	-1.670207	1.250472	2.487791
H	-0.913917	2.000388	2.622701
C	1.668184	1.250792	2.489272
H	0.910937	1.999645	2.624616
C	2.697970	1.142772	3.406143
H	2.714944	1.795946	4.265309
C	3.686358	0.196452	3.188480
H	4.504671	0.076771	3.883925
C	3.617512	-0.584626	2.044593
H	4.374015	-1.315118	1.813191
C	2.552880	-0.408889	1.175521
C	2.456092	-1.198060	-0.136632
C	1.157343	-1.999729	-0.043134
C	1.193259	-3.343989	0.295287
H	2.133779	-3.837412	0.457953
C	0.002666	-4.021668	0.481496
H	0.003284	-5.060147	0.778846
C	-1.188735	-3.345322	0.295585
H	-2.128672	-3.839798	0.458443
C	-1.154408	-2.001045	-0.042908
C	-2.454167	-1.200977	-0.136615
C	-1.645650	1.778590	-2.184617
H	-0.875883	2.524477	-2.171798
C	-2.723382	1.929359	-3.034419
H	-2.757281	2.771827	-3.708264
C	-3.749149	1.003011	-2.964039
H	-4.626295	1.090537	-3.588722
C	-3.645369	-0.028940	-2.046082
H	-4.457902	-0.721182	-1.928193
C	-2.552252	-0.411474	1.175167
C	-2.700243	1.142015	3.404305
H	-2.718468	1.796017	4.262813
C	-3.687311	0.194183	3.187120
H	-4.505757	0.074113	3.882340
C	-3.617041	-0.587804	2.043945
H	-4.372584	-1.319375	1.812805
C	2.510762	-0.136991	-1.252498
C	3.646258	-0.024352	-2.045735
H	4.459077	-0.716380	-1.928662
C	3.749654	1.008644	-2.962573
H	4.626685	1.097054	-3.587294
C	2.723810	1.934992	-3.031544
H	2.757595	2.778465	-3.704138
C	1.646135	1.782883	-2.181906
H	0.876620	2.529021	-2.167544
C	-2.509742	-0.140367	-1.252857
H	-3.651736	-3.982078	-0.955883
H	-2.901215	-2.811829	-2.056111
H	-4.654668	-2.789154	-1.788245
H	3.656616	-3.977957	-0.955462
H	4.658336	-2.784197	-1.788059
H	2.904901	-2.808700	-2.055928
N	-0.002474	2.573072	0.262178
C	-0.005014	3.710624	0.404549
C	-0.009267	5.145193	0.586076
H	1.011931	5.501967	0.711807
H	-0.453140	5.623481	-0.286069
H	-0.591699	5.400355	1.470407

$^{5}[Fe^{II}(Py5OMe)MeCN]^{2+}$

68

Fe	-0.006054	0.773710	0.095154
O	-3.538323	-2.122106	-0.387610
C	-3.500567	-3.003073	-1.524878
N	0.010675	-1.350899	-0.311149
N	1.593177	0.833671	-1.380446
O	3.564759	-2.089360	-0.402765
C	3.529656	-2.972692	-1.538127
N	1.747691	0.398409	1.468371
N	-1.648431	0.865992	-1.331073
N	-1.705621	0.341985	1.500209
C	-1.834389	0.995030	2.660909
H	-1.045494	1.687490	2.910784
C	1.894545	1.073134	2.614212
H	1.120273	1.786320	2.851744
C	2.978361	0.896825	3.457726
H	3.046050	1.462922	4.374342
C	3.959736	-0.011130	3.087576
H	4.822887	-0.177306	3.716456
C	3.826216	-0.703457	1.890589
H	4.570753	-1.409475	1.563219
C	2.704797	-0.471429	1.109693
C	2.493009	-1.191695	-0.238911
C	1.172979	-1.975958	-0.091177
C	1.215852	-3.264740	0.423749
H	2.159603	-3.727271	0.657803
C	0.022195	-3.916605	0.684433
H	0.026209	-4.909713	1.110150
C	-1.176434	-3.275999	0.421361
H	-2.115604	-3.747709	0.655027
C	-1.145948	-1.986105	-0.094511
C	-2.474940	-1.213178	-0.229717
C	-1.814176	1.911697	-2.150907
H	-1.064250	2.684396	-2.095268
C	-2.893636	2.041373	-3.003629
H	-2.975626	2.903350	-3.648119
C	-3.863898	1.051984	-2.975190
H	-4.739349	1.116260	-3.605535
C	-3.714333	-0.017448	-2.105041
H	-4.486626	-0.762448	-2.037902
C	-2.675599	-0.509677	1.129342
C	-2.917115	0.818468	3.505498
H	-2.971508	1.366092	4.434037
C	-3.913304	-0.067403	3.122067
H	-4.775966	-0.232884	3.751768
C	-3.794930	-0.740545	1.912317
H	-4.549698	-1.431853	1.576950
C	2.563598	-0.090916	-1.325585
C	3.695569	0.015727	-2.127984
H	4.495788	-0.697170	-2.038284
C	3.801256	1.064401	-3.028921
H	4.675161	1.149147	-3.658954
C	2.784580	2.004475	-3.093979
H	2.826476	2.845055	-3.769761
C	1.708944	1.854156	-2.240323
H	0.918537	2.586259	-2.222711
C	-2.579491	-0.099097	-1.303303
H	-3.120632	-3.981680	-1.238228
H	-2.882691	-2.608670	-2.330555
H	-4.524301	-3.107157	-1.875763
H	3.160239	-3.954231	-1.247683
H	4.552668	-3.067392	-1.893707
H	2.904579	-2.585486	-2.341762
N	-0.009878	2.847351	0.522727
C	-0.014994	3.976674	0.723460
C	-0.023030	5.399816	0.972896
H	0.998790	5.760617	1.079748
H	-0.503849	5.912168	0.140581
H	-0.576824	5.604376	1.888283

$^2[Fe^{III}(Py5OMe)MeCN]^{3+}$

68

Fe	-0.000622	-0.635145	-0.015527
O	-3.565901	2.057727	0.152206
C	-3.705858	2.992706	1.246380
N	0.002700	1.332102	0.100772
N	1.500237	-0.734504	1.315932
O	3.573053	2.047594	0.152238
C	3.712286	2.987674	1.242096
N	1.556910	-0.504936	-1.376021
N	-1.504176	-0.733427	1.311496
N	-1.556350	-0.500121	-1.376415
C	-1.651927	-1.315556	-2.438817
H	-0.899575	-2.070593	-2.552861
C	1.649285	-1.318934	-2.439825
H	0.894158	-2.070972	-2.555238
C	2.676391	-1.240163	-3.360244
H	2.686447	-1.920895	-4.197267
C	3.668162	-0.291646	-3.175433
H	4.483981	-0.197394	-3.877126
C	3.609177	0.524791	-2.055755
H	4.370048	1.257426	-1.846690
C	2.550232	0.382916	-1.178899
C	2.461961	1.206149	0.109462
C	1.164185	1.998729	-0.008698
C	1.197617	3.336749	-0.358535
H	2.139167	3.827792	-0.520092
C	0.007352	4.011765	-0.555379
H	0.009178	5.046471	-0.864541
C	-1.185272	3.340632	-0.359498
H	-2.125015	3.834767	-0.521952
C	-1.156461	2.002643	-0.009027
C	-2.456684	1.213942	0.108630
C	-1.623987	-1.745297	2.188009
H	-0.868476	-2.502998	2.172181
C	-2.685649	-1.871146	3.057915
H	-2.714275	-2.707045	3.739377
C	-3.700973	-0.933347	2.997296
H	-4.565107	-1.003112	3.641514
C	-3.608273	0.085877	2.063580
H	-4.415641	0.784596	1.952782
C	-2.546761	0.391264	-1.180077
C	-2.678875	-1.234424	-3.359185
H	-2.691695	-1.916540	-4.195041
C	-3.667022	-0.281838	-3.175772
H	-4.482560	-0.185667	-3.877528
C	-3.605121	0.535870	-2.057151
H	-4.363504	1.271309	-1.848847
C	2.494366	0.171149	1.247222
C	3.608800	0.075494	2.065790
H	4.420341	0.768860	1.952284
C	3.695114	-0.940298	3.003814
H	4.558870	-1.012916	3.648226
C	2.672989	-1.870285	3.069569
H	2.695332	-2.702401	3.755881
C	1.612427	-1.741729	2.198708
H	0.850654	-2.493046	2.188632
C	-2.493384	0.177786	1.245266
H	-3.673460	4.004679	0.851385
H	-2.925609	2.872107	1.995173
H	-4.677564	2.828463	1.704821
H	3.682315	3.997707	0.841925
H	4.682802	2.824197	1.703347
H	2.930236	2.872223	1.989806
N	-0.002258	-2.575398	-0.196150
C	-0.003926	-3.712625	-0.328668
C	-0.007892	-5.142598	-0.495926
H	1.014182	-5.495481	-0.624117
H	-0.448379	-5.605138	0.386909
H	-0.600989	-5.398693	-1.373184

$^{1}[Fe^{II}(Py5OH)H_2O]^{2+}$

59

Fe	0.000063	-0.003350	-0.745456
O	3.496590	0.000017	2.016664
H	4.334173	0.011034	1.540644
O	-0.000028	0.048486	-2.799542
N	-0.000212	-0.006508	1.253860
N	-1.513852	1.351471	-0.734152
O	-3.497201	-0.000129	2.015885
H	-4.334683	0.010941	1.539686
N	-1.515880	-1.355161	-0.735477
N	1.513912	1.351391	-0.733924
N	1.516083	-1.354978	-0.735344
C	1.625945	-2.360302	-1.616747
H	0.909339	-2.379892	-2.416584
C	-1.625081	-2.360927	-1.616465
H	-0.908138	-2.380682	-2.415986
C	-2.605759	-3.332798	-1.545474
H	-2.634585	-4.116798	-2.286681
C	-3.521483	-3.272201	-0.508562
H	-4.287700	-4.025556	-0.396521
C	-3.450632	-2.208081	0.378237
H	-4.157701	-2.126780	1.187013
C	-2.458279	-1.252136	0.218573
C	-2.422800	-0.000872	1.112601
C	-1.151765	-0.009084	1.943372
C	-1.193958	-0.018030	3.327553
H	-2.144216	-0.020509	3.832772
C	-0.000539	-0.023476	4.029656
H	-0.000666	-0.031170	5.110034
C	1.193047	-0.018006	3.327836
H	2.143182	-0.020453	3.833284
C	1.151176	-0.009065	1.943644
C	2.422382	-0.000796	1.113162
C	1.622230	2.355238	-1.617419
H	0.916990	2.358573	-2.427463
C	2.591764	3.337640	-1.535289
H	2.621454	4.121101	-2.277080
C	3.496172	3.288322	-0.487766
H	4.252027	4.050819	-0.367250
C	3.429662	2.222485	0.397514
H	4.130988	2.147552	1.211971
C	2.458199	-1.251980	0.219003
C	2.606897	-3.331904	-1.545810
H	2.636261	-4.115557	-2.287360
C	3.522180	-3.271507	-0.508498
H	4.288550	-4.024707	-0.396459
C	3.450727	-2.207738	0.378678
H	4.157493	-2.126563	1.187734
C	-2.449436	1.256211	0.226767
C	-3.429990	2.222370	0.396778
H	-4.131584	2.147339	1.210995
C	-3.496238	3.288330	-0.488371
H	-4.252168	4.050776	-0.367999
C	-2.591459	3.337845	-1.535561
H	-2.620893	4.121440	-2.277219
C	-1.621882	2.355471	-1.617511
H	-0.916360	2.358941	-2.427304
C	2.449195	1.256276	0.227301
H	0.779165	-0.080227	-3.350417
H	-0.779450	-0.080703	-3.349977

$^{5}[Fe^{II}(Py5OH)H_2O]^{2+}$

59

Fe	0.000452	-0.013365	-0.956880
O	3.506662	-0.007005	2.017703
H	4.355958	0.029341	1.564077
O	-0.003870	0.261364	-3.110900
N	0.001485	-0.029789	1.232662
N	-1.650575	1.412507	-0.779443
O	-3.502054	-0.019364	2.024230
H	-4.352292	0.009662	1.571842
N	-1.670923	-1.427213	-0.806666
N	1.670953	1.384928	-0.814549
N	1.645690	-1.460875	-0.771957
C	1.744304	-2.536457	-1.564259
H	1.017341	-2.618428	-2.356634
C	-1.790237	-2.480096	-1.626607
H	-1.086118	-2.533446	-2.442246
C	-2.757524	-3.455063	-1.467957
H	-2.810901	-4.281806	-2.159841
C	-3.629239	-3.340418	-0.396731
H	-4.385152	-4.091129	-0.215772
C	-3.525672	-2.241535	0.444899
H	-4.191286	-2.141926	1.285718
C	-2.548228	-1.284316	0.197996
C	-2.455431	-0.017864	1.083668
C	-1.156648	-0.031735	1.906594
C	-1.194755	-0.033686	3.292856
H	-2.138866	-0.035561	3.808847
C	0.004263	-0.032006	3.987230
H	0.005392	-0.035151	5.067910
C	1.201873	-0.024793	3.290419
H	2.147002	-0.019785	3.804513
C	1.161033	-0.025068	1.904264
C	2.458458	-0.015443	1.078879
C	1.764671	2.452514	-1.618480
H	1.083110	2.479019	-2.453707
C	2.681094	3.468056	-1.416482
H	2.717336	4.306240	-2.095690
C	3.525623	3.378404	-0.321162
H	4.240873	4.159910	-0.108296
C	3.448676	2.263453	0.502252
H	4.094327	2.180504	1.360467
C	2.544612	-1.294053	0.210084
C	2.717782	-3.505530	-1.404903
H	2.753526	-4.351172	-2.074737
C	3.618527	-3.360150	-0.362076
H	4.382282	-4.103279	-0.182720
C	3.531455	-2.241704	0.455333
H	4.216114	-2.121018	1.277806
C	-2.518179	1.275191	0.233390
C	-3.449007	2.264760	0.527223
H	-4.107666	2.164822	1.373657
C	-3.514789	3.395098	-0.275941
H	-4.235301	4.170971	-0.060215
C	-2.650844	3.508281	-1.353782
H	-2.675433	4.360728	-2.015523
C	-1.730544	2.497180	-1.561199
H	-1.033986	2.543462	-2.382919
C	2.524374	1.266508	0.212488
H	0.762411	0.046818	-3.655443
H	-0.786635	0.094678	-3.648792

$^{2}[Fe^{III}(Py5OH)H_2O]^{3+}$

59

Fe	-0.000143	0.000088	-0.746664
O	-3.495491	0.000025	2.019552
H	-4.341381	0.000335	1.556946
O	-0.000432	0.000199	-2.715533
N	0.000286	-0.000349	1.244530
N	1.520813	-1.333773	-0.738274
O	3.496404	-0.000171	2.018027
H	4.342090	-0.000265	1.555041
N	1.510243	1.346602	-0.726458
N	-1.510684	-1.346430	-0.725967
N	-1.520963	1.333895	-0.737474
C	-1.615261	2.331545	-1.631859
H	-0.912126	2.335191	-2.441840
C	1.593221	2.357121	-1.607277
H	0.881607	2.371531	-2.409456
C	2.557419	3.342060	-1.528889
H	2.570723	4.128727	-2.267025
C	3.474297	3.291883	-0.492982
H	4.226534	4.058238	-0.377799
C	3.425628	2.222581	0.388905
H	4.135228	2.146063	1.195723
C	2.452437	1.252300	0.227735
C	2.433695	0.000324	1.114298
C	1.157446	-0.001814	1.931461
C	1.194715	-0.002785	3.312326
H	2.145603	-0.004320	3.815890
C	0.000880	-0.000964	4.013073
H	0.001111	-0.001224	5.093132
C	-1.193254	0.001271	3.312839
H	-2.143925	0.002675	3.816815
C	-1.156578	0.000976	1.931956
C	-2.433187	-0.000495	1.115349
C	-1.594060	-2.356721	-1.607002
H	-0.882626	-2.371113	-2.409342
C	-2.558461	-3.341473	-1.528637
H	-2.572085	-4.127960	-2.266960
C	-3.475124	-3.291344	-0.492542
H	-4.227525	-4.057545	-0.377411
C	-3.426008	-2.222286	0.389613
H	-4.135380	-2.145821	1.196637
C	-2.457547	1.246205	0.222403
C	-2.582117	3.314160	-1.558193
H	-2.604865	4.090667	-2.306784
C	-3.489842	3.274845	-0.513638
H	-4.242861	4.040884	-0.401511
C	-3.432437	2.215512	0.379676
H	-4.136730	2.145541	1.191755
C	2.457917	-1.246147	0.221071
C	3.433024	-2.215347	0.377658
H	4.137770	-2.145397	1.189347
C	3.490031	-3.274557	-0.515823
H	4.243207	-4.040520	-0.404243
C	2.581674	-3.313848	-1.559829
H	2.604043	-4.090274	-2.308518
C	1.614678	-2.331309	-1.632829
H	0.911060	-2.334950	-2.442395
C	-2.452615	-1.252200	0.228463
H	-0.785645	-0.013644	-3.279755
H	0.784755	0.013912	-3.279796

[1][Fe^{II}(Py5OMe)H_2O]^{2+}

65

Fe	-0.000641	0.183966	-0.801964
O	3.576822	-0.343295	1.815578
C	3.721591	0.495210	2.978680
N	0.003436	-0.192290	1.120636
N	-1.516503	1.494310	-0.581349
O	-3.565823	-0.342559	1.830852
C	-3.705395	0.496632	2.994115
N	-1.554507	-1.176141	-1.039522
N	1.502557	1.507203	-0.565865
N	1.558004	-1.168429	-1.049189
C	1.661919	-1.987470	-2.105960
H	0.900471	-1.901977	-2.858562
C	-1.660227	-2.001652	-2.090883
H	-0.898980	-1.922280	-2.844371
C	-2.691549	-2.909758	-2.248426
H	-2.710698	-3.546609	-3.119668
C	-3.677083	-2.971437	-1.276227
H	-4.494987	-3.672893	-1.357257
C	-3.606720	-2.096550	-0.201692
H	-4.362126	-2.083003	0.565744
C	-2.544242	-1.211231	-0.125925
C	-2.451689	-0.166573	0.995752
C	-1.152492	-0.468468	1.743644
C	-1.183484	-1.145555	2.953110
H	-2.121978	-1.432909	3.390443
C	0.009113	-1.501654	3.555153
H	0.011219	-2.059165	4.480365
C	1.199025	-1.147334	2.946610
H	2.139574	-1.435771	3.378865
C	1.162620	-0.470117	1.737544
C	2.459378	-0.167083	0.984994
C	1.622301	2.615631	-1.313432
H	0.839589	2.804700	-2.020840
C	2.704513	3.471113	-1.246880
H	2.730571	4.347781	-1.875923
C	3.743779	3.150303	-0.391224
H	4.623636	3.773397	-0.321888
C	3.649993	1.988115	0.357233
H	4.471790	1.686877	0.979742
C	2.549878	-1.208033	-0.138799
C	2.692401	-2.895218	-2.270132
H	2.709783	-3.527172	-3.144976
C	3.679601	-2.962865	-1.299906
H	4.496698	-3.664643	-1.386096
C	3.612155	-2.093108	-0.221064
H	4.369468	-2.083086	0.544533
C	-2.511734	1.194292	0.272735
C	-3.650515	1.984520	0.367077
H	-4.460857	1.693721	1.009025
C	-3.761481	3.131342	-0.402496
H	-4.640979	3.754333	-0.327851
C	-2.742807	3.433616	-1.289079
H	-2.786701	4.293231	-1.940279
C	-1.658392	2.581176	-1.356521
H	-0.894105	2.751028	-2.089151
C	2.511335	1.197211	0.268604
H	3.655471	-0.118088	3.874404
H	2.963835	1.275274	3.024393
H	4.706982	0.954345	2.943239
H	-3.632382	-0.115252	3.890253
H	-4.692194	0.953166	2.964236
H	-2.949266	1.278593	3.034325
O	0.009097	0.559575	-2.824397
H	-0.758231	0.461028	-3.397826
H	0.798641	0.499605	-3.372690

$^5[Fe^{II}(Py5OMe)H_2O]^{2+}$

65

Fe	-0.001462	0.231663	-0.984013
O	3.547675	-0.386660	1.883446
C	3.518175	0.357520	3.114537
N	-0.003458	-0.170887	1.121415
N	-1.634904	1.588835	-0.570287
O	-3.552779	-0.308851	1.894698
C	-3.496139	0.461921	3.108376
N	-1.759253	-1.237870	-1.088541
N	1.610374	1.626932	-0.469114
N	1.751712	-1.193570	-1.132147
C	1.891971	-2.023733	-2.173292
H	1.114058	-1.989848	-2.921318
C	-1.899699	-2.109505	-2.094797
H	-1.114540	-2.117645	-2.836008
C	-2.985482	-2.962722	-2.205476
H	-3.048607	-3.649730	-3.035958
C	-3.970463	-2.906853	-1.230136
H	-4.833009	-3.556505	-1.278997
C	-3.838162	-2.001717	-0.184761
H	-4.581526	-1.926473	0.591326
C	-2.718754	-1.184889	-0.151673
C	-2.497088	-0.158613	0.978914
C	-1.167621	-0.576990	1.643917
C	-1.209043	-1.507545	2.674300
H	-2.154345	-1.871596	3.039401
C	-0.017602	-2.000895	3.177158
H	-0.023751	-2.749962	3.956020
C	1.181967	-1.543371	2.658570
H	2.120544	-1.936096	3.011079
C	1.155663	-0.607036	1.634240
C	2.490655	-0.196428	0.975637
C	1.732426	2.811325	-1.083317
H	0.935711	3.083072	-1.755243
C	2.821615	3.644730	-0.910020
H	2.869023	4.589651	-1.430027
C	3.843008	3.215662	-0.076771
H	4.726074	3.820254	0.074349
C	3.727541	1.986262	0.554513
H	4.528099	1.623452	1.174657
C	2.703499	-1.187665	-0.186165
C	2.969853	-2.882171	-2.310828
H	3.034069	-3.533534	-3.169451
C	3.946504	-2.877318	-1.325397
H	4.802915	-3.533216	-1.394259
C	3.814710	-2.014763	-0.244689
H	4.553213	-1.978444	0.538718
C	-2.584436	1.234062	0.307552
C	-3.716392	2.022051	0.495097
H	-4.497944	1.706024	1.162942
C	-3.849385	3.208193	-0.211082
H	-4.723701	3.827105	-0.067305
C	-2.861659	3.571603	-1.113436
H	-2.929883	4.474991	-1.700500
C	-1.779929	2.725090	-1.265632
H	-1.010162	2.938893	-1.989258
C	2.583244	1.219166	0.357455
H	3.122161	-0.253709	3.923229
H	2.918990	1.263959	3.034663
H	4.546822	0.627037	3.341581
H	-3.084712	-0.131589	3.922673
H	-4.519288	0.739298	3.349991
H	-2.896173	1.364202	2.994628
O	0.161965	0.818739	-3.054723
H	-0.549090	0.766826	-3.703441
H	0.993009	0.808223	-3.542577

$^{2}[Fe^{III}(Py5OMe)H_2O]^{3+}$

65

Fe	-0.000265	0.153523	-0.806679
O	3.578571	-0.292168	1.820740
C	3.730526	0.568743	2.973121
N	0.003151	-0.167042	1.115484
N	-1.517190	1.454480	-0.632887
O	-3.568589	-0.291744	1.834512
C	-3.718686	0.571489	2.985432
N	-1.549643	-1.202169	-0.996686
N	1.493103	1.477367	-0.603785
N	1.564095	-1.182488	-1.016224
C	1.658314	-2.016387	-2.064154
H	0.902104	-1.943559	-2.821198
C	-1.634175	-2.056820	-2.028174
H	-0.870911	-1.999256	-2.779273
C	-2.662436	-2.969131	-2.165899
H	-2.669021	-3.629808	-3.018835
C	-3.658607	-3.002949	-1.204297
H	-4.474353	-3.707493	-1.274834
C	-3.603629	-2.097604	-0.153979
H	-4.366682	-2.061081	0.605018
C	-2.546196	-1.210442	-0.090893
C	-2.461444	-0.138491	1.002432
C	-1.158660	-0.422659	1.743955
C	-1.184600	-1.061765	2.970065
H	-2.123548	-1.331841	3.416519
C	0.008745	-1.405160	3.577666
H	0.010625	-1.938984	4.516433
C	1.199636	-1.069363	2.960860
H	2.140580	-1.344796	3.399972
C	1.168366	-0.428190	1.736258
C	2.468946	-0.139246	0.991902
C	1.586789	2.568789	-1.382110
H	0.809912	2.733395	-2.099923
C	2.649676	3.445445	-1.326288
H	2.659789	4.306008	-1.976750
C	3.688274	3.167589	-0.455685
H	4.552844	3.812029	-0.395752
C	3.617310	2.022075	0.320227
H	4.440421	1.752741	0.955233
C	2.557622	-1.201595	-0.108120
C	2.689224	-2.923943	-2.211625
H	2.703049	-3.568489	-3.076728
C	3.678868	-2.974139	-1.243817
H	4.495580	-3.676834	-1.321205
C	3.617399	-2.085492	-0.179819
H	4.377599	-2.058173	0.582405
C	-2.505858	1.197570	0.242534
C	-3.623584	2.013639	0.322217
H	-4.430570	1.760201	0.983547
C	-3.720391	3.134288	-0.486392
H	-4.585433	3.777702	-0.422487
C	-2.711362	3.383434	-1.399431
H	-2.748097	4.217073	-2.083166
C	-1.644063	2.512440	-1.452510
H	-0.893313	2.649763	-2.203827
C	2.501085	1.204070	0.244549
H	3.666058	-0.032244	3.876255
H	2.975802	1.351841	3.006740
H	4.718401	1.018890	2.922337
H	-3.648433	-0.026574	3.890064
H	-4.708485	1.017764	2.937789
H	-2.966516	1.357233	3.013775
O	0.007510	0.391495	-2.755984
H	-0.775362	0.443442	-3.319282
H	0.797764	0.490569	-3.302762

$^6[Fe^{III}(Py5OMe)H_2O]^{3+}$

65

Fe	0.000472	0.163206	-0.914274
O	3.538073	-0.226711	1.903945
C	3.595527	0.650472	3.051510
N	-0.023921	-0.127796	1.136378
N	-1.520337	1.595299	-0.549669
O	-3.609310	-0.282116	1.798231
C	-3.724203	0.562890	2.965229
N	-1.672161	-1.191355	-1.090196
N	1.623856	1.478986	-0.707270
N	1.627712	-1.299711	-0.954168
C	1.713183	-2.229643	-1.916164
H	0.930903	-2.238806	-2.655207
C	-1.765765	-2.051463	-2.116430
H	-0.999125	-1.990502	-2.869444
C	-2.800463	-2.958414	-2.233038
H	-2.829370	-3.628998	-3.077605
C	-3.781716	-2.972194	-1.253562
H	-4.605564	-3.668720	-1.308770
C	-3.705039	-2.067878	-0.201204
H	-4.456814	-2.034639	0.568712
C	-2.639950	-1.188776	-0.154834
C	-2.508993	-0.129416	0.953446
C	-1.200508	-0.444826	1.705516
C	-1.242188	-1.158993	2.889625
H	-2.183487	-1.475724	3.300927
C	-0.051906	-1.509559	3.503858
H	-0.061537	-2.096196	4.410525
C	1.150936	-1.122208	2.939163
H	2.083321	-1.414879	3.386968
C	1.136385	-0.413420	1.749605
C	2.461141	-0.111212	1.024376
C	1.797242	2.512665	-1.548822
H	1.062876	2.634362	-2.326513
C	2.877183	3.364776	-1.468291
H	2.968497	4.182872	-2.165272
C	3.833682	3.114205	-0.498220
H	4.706652	3.743598	-0.407346
C	3.681584	2.017784	0.338900
H	4.454047	1.778660	1.045377
C	2.603171	-1.228949	-0.029242
C	2.756087	-3.134365	-1.982726
H	2.778770	-3.864981	-2.776187
C	3.750856	-3.069935	-1.019549
H	4.581174	-3.760696	-1.037679
C	3.676511	-2.098081	-0.028575
H	4.434392	-2.008329	0.730997
C	-2.550235	1.242792	0.242143
C	-3.680259	2.039992	0.328328
H	-4.526992	1.718223	0.906258
C	-3.725192	3.246271	-0.355979
H	-4.601925	3.873274	-0.287591
C	-2.641379	3.623755	-1.131445
H	-2.626423	4.551119	-1.682158
C	-1.567898	2.762014	-1.213758
H	-0.728118	3.002568	-1.839201
C	2.556366	1.215348	0.227749
H	3.394283	0.083910	3.956647
H	2.883800	1.470474	2.978631
H	4.605078	1.049071	3.102403
H	-3.624004	-0.047406	3.858697
H	-4.714593	1.009871	2.952537
H	-2.972983	1.349858	2.981510
O	0.031029	0.346232	-2.991911
H	-0.732093	0.530497	-3.556251
H	0.817618	0.339157	-3.553128

$^{1}[Fe^{II}(Py5OH)OH]^{+}$

58

Fe	-0.001841	0.000020	-0.772278
O	3.530665	-0.000017	1.968834
H	4.357074	-0.000104	1.473751
O	-0.063828	-0.000228	-2.723116
N	0.020209	0.000049	1.257058
N	-1.521705	1.329398	-0.733980
O	-3.469659	0.000046	2.052035
H	-4.306579	-0.000003	1.575093
N	-1.521593	-1.329487	-0.733841
N	1.502069	1.347794	-0.750355
N	1.502119	-1.347658	-0.750438
C	1.585556	-2.351630	-1.635592
H	0.859049	-2.333705	-2.426995
C	-1.657667	-2.291691	-1.656110
H	-0.977598	-2.226547	-2.486377
C	-2.633243	-3.270905	-1.583554
H	-2.691129	-4.026633	-2.352595
C	-3.514102	-3.252185	-0.513830
H	-4.272775	-4.013822	-0.403297
C	-3.426892	-2.213230	0.402810
H	-4.117931	-2.154161	1.227845
C	-2.440138	-1.250533	0.243619
C	-2.398112	0.000033	1.141266
C	-1.121319	0.000068	1.962325
C	-1.147643	0.000082	3.347720
H	-2.091551	0.000099	3.865289
C	0.054938	0.000065	4.035522
H	0.068539	0.000073	5.116029
C	1.240040	0.000030	3.317802
H	2.196467	0.000006	3.811769
C	1.179478	0.000023	1.933588
C	2.438764	-0.000011	1.082313
C	1.585456	2.351938	-1.635324
H	0.858861	2.334287	-2.426644
C	2.553606	3.338399	-1.570599
H	2.565329	4.125020	-2.310088
C	3.483779	3.288568	-0.544806
H	4.241471	4.052108	-0.441526
C	3.439632	2.221751	0.341619
H	4.160250	2.146956	1.139448
C	2.455318	-1.254699	0.192652
C	2.553674	-3.338129	-1.570926
H	2.565456	-4.124632	-2.310540
C	3.483744	-3.288495	-0.545025
H	4.241384	-4.052092	-0.441781
C	3.439580	-2.221797	0.341546
H	4.160147	-2.147137	1.139436
C	-2.440209	1.250517	0.243521
C	-3.427025	2.213166	0.402618
H	-4.118025	2.154163	1.227691
C	-3.514358	3.251972	-0.514179
H	-4.273081	4.013571	-0.403727
C	-2.633585	3.270563	-1.583976
H	-2.691599	4.026136	-2.353159
C	-1.657940	2.291410	-1.656427
H	-0.977978	2.226111	-2.486768
C	2.455326	1.254706	0.192677
H	0.827022	0.000798	-3.082249

$^{5}[Fe^{II}(Py5OH)OH]^{+}$

58

Fe	0.008861	-0.000799	-1.135642
O	-3.512282	0.001029	2.022938
H	-4.359240	0.001669	1.564287
O	0.029363	-0.002556	-3.050259
N	-0.005558	0.000087	1.232096
N	1.671770	-1.411009	-0.783076
O	3.495783	0.000613	2.045551
H	4.345298	0.001295	1.591550
N	1.671443	1.410479	-0.783766
N	-1.659042	-1.424967	-0.780237
N	-1.658467	1.424402	-0.781211
C	-1.754046	2.488010	-1.587231
H	-1.043538	2.523959	-2.398909
C	1.784276	2.459764	-1.606061
H	1.091902	2.477593	-2.433890
C	2.731771	3.452921	-1.430847
H	2.784588	4.280880	-2.121887
C	3.588865	3.353948	-0.344866
H	4.328340	4.118355	-0.152237
C	3.494516	2.251582	0.493800
H	4.152566	2.160384	1.342034
C	2.535406	1.278244	0.229443
C	2.449939	0.000221	1.098718
C	1.146341	-0.000255	1.912536
C	1.184474	-0.000988	3.301234
H	2.127656	-0.001267	3.819138
C	-0.014874	-0.001314	3.994206
H	-0.018627	-0.001896	5.075165
C	-1.209528	-0.000846	3.293049
H	-2.156211	-0.001011	3.804498
C	-1.161990	-0.000124	1.904730
C	-2.460621	0.000436	1.082316
C	-1.755136	-2.488812	-1.585881
H	-1.044035	-2.525857	-2.396987
C	-2.705559	-3.479889	-1.414717
H	-2.742888	-4.319388	-2.092711
C	-3.585163	-3.363155	-0.348981
H	-4.329667	-4.123599	-0.160076
C	-3.504667	-2.249579	0.476037
H	-4.177395	-2.148130	1.311370
C	-2.538356	1.281272	0.216660
C	-2.703213	3.480228	-1.415733
H	-2.740144	4.319484	-2.094049
C	-3.582051	3.364947	-0.349209
H	-4.325483	4.126351	-0.159957
C	-3.502206	2.251573	0.476142
H	-4.174442	2.151191	1.312004
C	2.536325	-1.277744	0.229487
C	3.496737	-2.249964	0.493205
H	4.155323	-2.157930	1.340930
C	3.591664	-3.352317	-0.345408
H	4.332186	-4.115848	-0.153308
C	2.733776	-3.452408	-1.430665
H	2.786948	-4.280421	-2.121615
C	1.785057	-2.460321	-1.605300
H	1.092073	-2.478985	-2.432597
C	-2.539472	-1.280512	0.216955
H	-0.813109	-0.001201	-3.510240

$^{2}[Fe^{III}(Py5OH)OH]^{2+}$

58

Fe	0.001864	-0.000056	-0.805769
O	-3.550144	0.000034	1.934869
H	-4.378488	0.000215	1.441727
O	0.071549	-0.000056	-2.611422
N	-0.035655	-0.000042	1.258355
N	1.533775	-1.320685	-0.737606
O	3.442785	0.000063	2.075888
H	4.289308	0.000121	1.615227
N	1.533638	1.320703	-0.737577
N	-1.503883	-1.347494	-0.766808
N	-1.503899	1.347356	-0.766899
C	-1.559674	2.365928	-1.638678
H	-0.827680	2.364474	-2.423993
C	1.678165	2.277258	-1.664922
H	1.018039	2.218004	-2.510318
C	2.646753	3.259487	-1.576626
H	2.715809	4.009527	-2.349422
C	3.504033	3.250319	-0.488796
H	4.256185	4.016191	-0.367164
C	3.405791	2.216219	0.431454
H	4.081068	2.162581	1.269373
C	2.430190	1.247711	0.259568
C	2.385363	0.000020	1.159228
C	1.102140	-0.000061	1.966721
C	1.115848	-0.000135	3.350514
H	2.054796	-0.000153	3.876336
C	-0.093854	-0.000176	4.024614
H	-0.116782	-0.000235	5.104717
C	-1.274625	-0.000129	3.299903
H	-2.234692	-0.000141	3.786069
C	-1.203478	-0.000062	1.918490
C	-2.457426	0.000003	1.060229
C	-1.559668	-2.366119	-1.638525
H	-0.827583	-2.364817	-2.423748
C	-2.514156	-3.362303	-1.565623
H	-2.507779	-4.158596	-2.293849
C	-3.451553	-3.309020	-0.547495
H	-4.199840	-4.080603	-0.439268
C	-3.428045	-2.232118	0.326377
H	-4.153306	-2.156401	1.119392
C	-2.458217	1.254314	0.173576
C	-2.514024	3.362237	-1.565698
H	-2.507653	4.158486	-2.293973
C	-3.451272	3.309145	-0.547420
H	-4.199421	4.080849	-0.439110
C	-3.427798	2.232277	0.326498
H	-4.152964	2.156697	1.119613
C	2.430320	-1.247640	0.259540
C	3.406031	-2.216044	0.431389
H	4.081297	-2.162366	1.269315
C	3.504399	-3.250087	-0.488910
H	4.256642	-4.015875	-0.367315
C	2.647142	-3.259283	-1.576757
H	2.716309	-4.009260	-2.349605
C	1.678443	-2.277162	-1.665012
H	1.018360	-2.217909	-2.510439
C	-2.458304	-1.254299	0.173552
H	-0.806585	-0.000192	-3.007664

$^{6}[Fe^{III}(Py5OH)OH]^{2+}$

58

Fe	0.014069	0.000020	-1.037748
O	-3.531665	-0.000000	1.983966
H	-4.375016	-0.000210	1.517524
O	0.023992	-0.000048	-2.858258
N	-0.022193	0.000099	1.236248
N	1.634040	-1.386800	-0.773448
O	3.470517	-0.000092	2.058699
H	4.323796	-0.000114	1.610763
N	1.634189	1.386674	-0.773497
N	-1.607657	-1.408890	-0.779740
N	-1.607529	1.409049	-0.779612
C	-1.679031	2.463467	-1.604474
H	-0.963047	2.489003	-2.408716
C	1.753317	2.409662	-1.631202
H	1.079926	2.403252	-2.472401
C	2.699922	3.403565	-1.472320
H	2.759485	4.209815	-2.187070
C	3.550132	3.330510	-0.380249
H	4.290420	4.097675	-0.204801
C	3.457402	2.245902	0.482525
H	4.119088	2.167213	1.328880
C	2.500828	1.270332	0.243889
C	2.427218	-0.000066	1.120781
C	1.125814	0.000053	1.927663
C	1.153040	-0.000035	3.313506
H	2.094169	-0.000112	3.834956
C	-0.050578	-0.000046	3.998733
H	-0.061550	-0.000109	5.079182
C	-1.240018	-0.000016	3.289072
H	-2.191960	-0.000053	3.790555
C	-1.183701	0.000034	1.904393
C	-2.467546	0.000055	1.069319
C	-1.679391	-2.463180	-1.604748
H	-0.963487	-2.488703	-2.409060
C	-2.624633	-3.460081	-1.458715
H	-2.642110	-4.290536	-2.147568
C	-3.527207	-3.358404	-0.412109
H	-4.271283	-4.124358	-0.247785
C	-3.476430	-2.249983	0.422465
H	-4.172590	-2.154811	1.238775
C	-2.514567	1.275239	0.199561
C	-2.624147	3.460479	-1.458392
H	-2.641423	4.291043	-2.147119
C	-3.526828	3.358790	-0.411881
H	-4.270788	4.124844	-0.247504
C	-3.476267	2.250262	0.422565
H	-4.172449	2.155131	1.238855
C	2.500659	-1.270528	0.243965
C	3.457055	-2.246252	0.482702
H	4.118704	-2.167633	1.329090
C	3.549646	-3.330923	-0.380008
H	4.289795	-4.098205	-0.204480
C	2.699490	-3.403888	-1.472128
H	2.758967	-4.210179	-2.186840
C	1.753055	-2.409842	-1.631104
H	1.079718	-2.403359	-2.472346
C	-2.514589	-1.275094	0.199534
H	-0.728720	0.000017	-3.456423

$^{5}[Fe^{II}(Py5OMe)OH]^{+}$

64

Fe	-0.010417	0.212655	1.140606
O	-3.546943	-0.268037	-1.920245
C	-3.476409	0.520982	-3.119091
N	-0.002812	-0.106014	-1.126973
N	1.627261	1.597716	0.579778
O	3.540679	-0.253410	-1.925551
C	3.466023	0.543614	-3.118527
N	1.760454	-1.268998	1.032736
N	-1.641839	1.585443	0.589617
N	-1.766132	-1.244745	1.052324
C	-1.914223	-2.105317	2.064395
H	-1.129899	-2.090486	2.806276
C	1.909509	-2.145135	2.031049
H	1.115754	-2.159013	2.763017
C	3.014440	-2.974778	2.141505
H	3.087819	-3.671436	2.963267
C	4.009283	-2.880905	1.177917
H	4.886747	-3.510274	1.228595
C	3.869354	-1.962352	0.145086
H	4.622365	-1.854011	-0.617780
C	2.727450	-1.175325	0.110642
C	2.490149	-0.124409	-0.992187
C	1.154323	-0.524643	-1.652008
C	1.192812	-1.450915	-2.688228
H	2.137288	-1.821206	-3.049752
C	-0.000121	-1.925374	-3.204320
H	0.001093	-2.665286	-3.992014
C	-1.194638	-1.456753	-2.686412
H	-2.137741	-1.831934	-3.046302
C	-1.158714	-0.529462	-1.651142
C	-2.494898	-0.131253	-0.990294
C	-1.787843	2.706696	1.305595
H	-1.019963	2.898163	2.037988
C	-2.867402	3.559337	1.164706
H	-2.935367	4.453197	1.766480
C	-3.854759	3.213510	0.254288
H	-4.728282	3.835850	0.120501
C	-3.721399	2.039683	-0.471986
H	-4.501785	1.733535	-1.146116
C	-2.725741	-1.175903	0.119924
C	-3.009424	-2.947424	2.175804
H	-3.083726	-3.630434	3.008859
C	-3.993853	-2.883978	1.199076
H	-4.862636	-3.525283	1.249811
C	-3.856026	-1.979085	0.153886
H	-4.602624	-1.892327	-0.617995
C	2.582288	1.253100	-0.293037
C	3.721563	2.038005	-0.459304
H	4.507911	1.724473	-1.123235
C	3.851503	3.215451	0.261486
H	4.729711	3.832769	0.135304
C	2.852782	3.572889	1.154798
H	2.915196	4.471903	1.749463
C	1.768234	2.725155	1.287296
H	0.989570	2.929595	2.004670
C	-2.588334	1.248487	-0.295709
H	-3.053370	-0.058793	-3.937771
H	-2.879441	1.422098	-2.983710
H	-4.496204	0.802593	-3.370623
H	3.023468	-0.024024	-3.935461
H	4.486931	0.810953	-3.380648
H	2.885103	1.453126	-2.969778
O	-0.012955	0.375226	3.040200
H	0.832585	0.479795	3.482314

$^{2}[Fe^{III}(Py5OMe)OH]^{2+}$

64

Fe	0.002686	0.163439	-0.870062
O	3.521966	-0.304075	1.883392
C	3.641638	0.538454	3.048560
N	-0.031631	-0.156844	1.124465
N	-1.481210	1.493085	-0.626220
O	-3.620542	-0.283055	1.747587
C	-3.778908	0.569585	2.901443
N	-1.548265	-1.187852	-1.046989
N	1.538153	1.439094	-0.646161
N	1.574406	-1.170683	-1.015730
C	1.714358	-1.960818	-2.088019
H	1.000708	-1.816181	-2.877274
C	-1.601308	-2.050412	-2.072462
H	-0.817628	-1.975363	-2.801778
C	-2.617971	-2.974666	-2.223928
H	-2.602327	-3.644793	-3.069632
C	-3.636339	-3.007019	-1.284675
H	-4.445842	-3.717665	-1.368426
C	-3.610945	-2.095106	-0.238979
H	-4.390116	-2.061471	0.503552
C	-2.558537	-1.200543	-0.159253
C	-2.489381	-0.133164	0.938364
C	-1.205052	-0.429544	1.716275
C	-1.266427	-1.093790	2.929561
H	-2.215808	-1.374199	3.347316
C	-0.087653	-1.447350	3.561079
H	-0.109658	-1.997571	4.490332
C	1.118766	-1.104138	2.979497
H	2.047601	-1.393236	3.435766
C	1.111931	-0.437789	1.764877
C	2.420213	-0.146099	1.036133
C	1.713631	2.456242	-1.502294
H	1.011044	2.519190	-2.311049
C	2.776316	3.333265	-1.417623
H	2.859805	4.138879	-2.130685
C	3.727803	3.121420	-0.435491
H	4.586031	3.770799	-0.341710
C	3.592741	2.022404	0.398658
H	4.368592	1.791126	1.103719
C	2.529714	-1.209275	-0.069322
C	2.744377	-2.875277	-2.213363
H	2.801253	-3.492010	-3.097153
C	3.685033	-2.963885	-1.199795
H	4.498991	-3.672019	-1.258905
C	3.587055	-2.099882	-0.117521
H	4.319590	-2.095073	0.671802
C	-2.505597	1.215035	0.197956
C	-3.616878	2.043436	0.267843
H	-4.457647	1.770078	0.877718
C	-3.655724	3.208554	-0.480645
H	-4.515324	3.860507	-0.426305
C	-2.589066	3.500728	-1.312725
H	-2.572032	4.383046	-1.933633
C	-1.537253	2.609247	-1.369927
H	-0.731474	2.770794	-2.057329
C	2.482402	1.198560	0.280228
H	3.559732	-0.073279	3.943681
H	2.881825	1.317168	3.076604
H	4.627514	0.996759	3.029597
H	-3.735917	-0.037140	3.802681
H	-4.759144	1.036015	2.841074
H	-3.014578	1.342654	2.953511
O	0.056645	0.399033	-2.663899
H	-0.823385	0.461697	-3.050763

$^{6}[Fe^{III}(Py5OMe)OH]^{2+}$

64

Fe	0.016187	0.194582	-1.065797
O	3.534370	-0.281662	1.902666
C	3.539402	0.529212	3.095140
N	-0.018057	-0.130819	1.115381
N	-1.575868	1.574847	-0.601153
O	-3.585803	-0.262911	1.837043
C	-3.614278	0.558208	3.022020
N	-1.677526	-1.247968	-1.050614
N	1.619795	1.535837	-0.625393
N	1.701519	-1.221835	-1.050418
C	1.836914	-2.067625	-2.080225
H	1.086560	-2.002373	-2.851165
C	-1.771297	-2.136645	-2.048712
H	-0.982264	-2.117354	-2.782224
C	-2.827650	-3.022980	-2.158615
H	-2.855195	-3.725077	-2.977848
C	-3.834075	-2.974102	-1.205380
H	-4.677095	-3.648137	-1.259059
C	-3.754636	-2.039373	-0.180537
H	-4.520699	-1.960893	0.572399
C	-2.660399	-1.192959	-0.137155
C	-2.499155	-0.126497	0.961271
C	-1.183200	-0.484156	1.675653
C	-1.230423	-1.301464	2.795852
H	-2.175657	-1.636289	3.185398
C	-0.042446	-1.729406	3.361245
H	-0.051852	-2.394411	4.212653
C	1.157751	-1.312678	2.813576
H	2.093729	-1.656636	3.217112
C	1.135334	-0.492795	1.694340
C	2.466014	-0.138042	1.006570
C	1.771056	2.624060	-1.393631
H	1.036478	2.764322	-2.168246
C	2.831793	3.495978	-1.250497
H	2.906840	4.362934	-1.888638
C	3.795215	3.198663	-0.300014
H	4.656319	3.837536	-0.166324
C	3.665366	2.045026	0.460414
H	4.441074	1.771591	1.151855
C	2.649113	-1.196834	-0.098145
C	2.893279	-2.955931	-2.175344
H	2.957826	-3.623837	-3.020631
C	3.852619	-2.952853	-1.173660
H	4.691944	-3.632475	-1.212944
C	3.736623	-2.051537	-0.122000
H	4.472817	-2.001863	0.662547
C	-2.559046	1.244518	0.250036
C	-3.683704	2.050109	0.381045
H	-4.497790	1.748287	1.014718
C	-3.767080	3.234708	-0.335259
H	-4.636614	3.867754	-0.232193
C	-2.736874	3.577652	-1.196306
H	-2.762551	4.479672	-1.788099
C	-1.671586	2.707597	-1.312840
H	-0.881118	2.898826	-2.017227
C	2.552251	1.231983	0.291121
H	3.288931	-0.079376	3.961009
H	2.838127	1.359542	3.031349
H	4.547881	0.917209	3.216077
H	-3.370381	-0.040267	3.896822
H	-4.627525	0.938813	3.124459
H	-2.919817	1.394376	2.960738
O	0.005664	0.430559	-2.867907
H	-0.745017	0.529641	-3.460631

$^{2}[Co^{II}(Py5OH)MeCN]^{2+}$

62

Co	-0.000707	0.617019	0.002332
O	-3.491027	-2.236271	-0.006904
H	-4.312967	-1.732301	-0.006283
N	0.003910	-1.581565	-0.004677
N	1.505146	0.540850	-1.337027
O	3.501521	-2.221055	-0.006947
H	4.321342	-1.713659	-0.006348
N	1.505153	0.532105	1.341079
N	-1.505783	0.533294	-1.337726
N	-1.505808	0.524662	1.341649
C	-1.617859	1.390241	2.358541
H	-0.950078	2.232090	2.345035
C	1.614663	1.399369	2.356804
H	0.944096	2.239033	2.342335
C	2.544669	1.264418	3.370431
H	2.581771	1.996362	4.162725
C	3.401891	0.177297	3.337034
H	4.125538	0.014774	4.122689
C	3.333838	-0.692724	2.258877
H	4.001742	-1.535630	2.195135
C	2.396977	-0.472239	1.258909
C	2.393368	-1.358555	-0.004102
C	1.155102	-2.245338	-0.006920
C	1.210215	-3.630746	-0.011537
H	2.159111	-4.139933	-0.013292
C	0.009889	-4.328664	-0.013822
H	0.012247	-5.409385	-0.017410
C	-1.193472	-3.635987	-0.011515
H	-2.140144	-4.149301	-0.013253
C	-1.144395	-2.250361	-0.006902
C	-2.386576	-1.369014	-0.004085
C	-1.617635	1.405036	-2.349353
H	-0.949918	2.246838	-2.330479
C	-2.546343	1.271798	-3.364380
H	-2.585380	2.007492	-4.153099
C	-3.399696	0.181482	-3.337021
H	-4.122207	0.020253	-4.123986
C	-3.329007	-0.693823	-2.263331
H	-3.993603	-1.539698	-2.204667
C	-2.393829	-0.482985	1.259038
C	-2.546917	1.250894	3.372413
H	-2.586130	1.981757	4.165605
C	-3.400413	0.160889	3.338134
H	-4.123191	-0.004991	4.123887
C	-3.329541	-0.707849	2.259135
H	-3.994237	-1.553239	2.194903
C	2.396814	-0.464157	-1.261398
C	3.333224	-0.678583	-2.263083
H	4.000975	-1.522020	-2.205008
C	3.401116	0.198127	-3.335819
H	4.124455	0.040296	-4.122713
C	2.544133	1.285635	-3.362137
H	2.581109	2.022517	-4.149845
C	1.614510	1.414415	-2.347344
H	0.944089	2.254087	-2.327341
C	-2.393699	-0.474970	-1.261548
N	-0.007906	2.844438	0.009471
C	-0.017008	3.992034	0.011481
C	-0.029032	5.439368	0.013479
H	-0.908232	5.798048	0.546547
H	-0.056124	5.806158	-1.011589
H	0.867962	5.813013	0.505013

$^{4}[Co^{II}(Py5OH)MeCN]^{2+}$

62

Co	0.000047	-0.001398	-0.683535
O	3.503288	0.024106	2.264571
H	4.350376	0.015171	1.805552
N	-0.000283	0.021703	1.487378
N	-1.638540	1.397268	-0.558113
O	-3.504070	0.024226	2.263505
H	-4.351025	0.015507	1.804236
N	-1.634487	-1.402541	-0.528203
N	1.638080	1.397905	-0.556946
N	1.634997	-1.401940	-0.528291
C	1.731820	-2.462735	-1.338503
H	1.035010	-2.502892	-2.159975
C	-1.730701	-2.463732	-1.337964
H	-1.033280	-2.504319	-2.158890
C	-2.672578	-3.461359	-1.163999
H	-2.707934	-4.296073	-1.847461
C	-3.544682	-3.357166	-0.091780
H	-4.281264	-4.124449	0.099076
C	-3.468461	-2.244848	0.735151
H	-4.137633	-2.146566	1.573605
C	-2.513847	-1.268958	0.474012
C	-2.449792	0.016129	1.331721
C	-1.157626	0.030198	2.159580
C	-1.199132	0.048810	3.545747
H	-2.144271	0.055186	4.060015
C	-0.000700	0.058283	4.241564
H	-0.000864	0.072515	5.322191
C	1.197944	0.048696	3.546114
H	2.142926	0.054982	4.060672
C	1.156861	0.030120	2.159933
C	2.449286	0.016149	1.332474
C	1.739156	2.439013	-1.391792
H	1.034994	2.467717	-2.207387
C	2.693502	3.429790	-1.248541
H	2.732855	4.248209	-1.951213
C	3.573229	3.339976	-0.181365
H	4.320568	4.102407	-0.014289
C	3.490354	2.248824	0.672454
H	4.164567	2.164711	1.508341
C	2.513732	-1.268733	0.474511
C	2.673580	-3.460431	-1.164333
H	2.709440	-4.294822	-1.848162
C	3.544904	-3.356736	-0.091429
H	4.281319	-4.124129	0.099626
C	3.468151	-2.244752	0.735905
H	4.136767	-2.146811	1.574843
C	-2.523033	1.277928	0.441452
C	-3.490641	2.248742	0.671143
H	-4.164764	2.164992	1.507141
C	-3.573579	3.339540	-0.183122
H	-4.320834	4.102097	-0.016238
C	-2.694079	3.428801	-1.250535
H	-2.733570	4.246869	-1.953608
C	-1.739781	2.437936	-1.393493
H	-1.035831	2.466151	-2.209302
C	2.522664	1.278155	0.442498
N	0.000533	-0.022221	-2.796069
C	0.001232	-0.049337	-3.942069
C	0.001974	-0.084904	-5.386209
H	0.882256	-0.621106	-5.737696
H	0.020951	0.932904	-5.773647
H	-0.896411	-0.588775	-5.739654

$^{1}[Co^{III}(Py5OH)MeCN]^{3+}$

62

Co	-0.000513	-0.001522	-0.527013
O	3.484522	0.004868	2.236456
H	4.327226	0.006478	1.767584
N	-0.006307	0.002253	1.466093
N	-1.494662	1.332360	-0.529937
O	-3.499126	0.003436	2.211849
H	-4.338162	0.004801	1.736518
N	-1.493965	-1.335957	-0.524532
N	1.479244	1.349265	-0.496857
N	1.480391	-1.351158	-0.491751
C	1.565978	-2.364343	-1.368097
H	0.862351	-2.382293	-2.174893
C	-1.602820	-2.319668	-1.431174
H	-0.918008	-2.310781	-2.254857
C	-2.568247	-3.303814	-1.356917
H	-2.598537	-4.070704	-2.115089
C	-3.465928	-3.275494	-0.304192
H	-4.217993	-4.042401	-0.191721
C	-3.404543	-2.219342	0.591890
H	-4.107734	-2.148588	1.404839
C	-2.430657	-1.248531	0.435628
C	-2.423443	0.001781	1.324250
C	-1.159935	0.002827	2.151587
C	-1.204325	0.004173	3.533379
H	-2.158641	0.004511	4.030650
C	-0.014348	0.004861	4.238955
H	-0.017330	0.005571	5.318964
C	1.179835	0.004524	3.540113
H	2.131099	0.005074	4.043214
C	1.144254	0.003243	2.158233
C	2.415683	0.002736	1.340268
C	1.563009	2.360290	-1.375879
H	0.859464	2.374974	-2.182993
C	2.521350	3.350349	-1.291887
H	2.530040	4.138798	-2.028191
C	3.438868	3.300707	-0.257343
H	4.188533	4.069089	-0.139115
C	3.394784	2.226223	0.617175
H	4.106434	2.146083	1.421745
C	2.425460	-1.250544	0.458393
C	2.526528	-3.352070	-1.282332
H	2.536707	-4.142096	-2.016935
C	3.444355	-3.297918	-0.248321
H	4.195949	-4.064215	-0.128736
C	3.398156	-2.221413	0.623663
H	4.109976	-2.137477	1.427628
C	-2.430410	1.249097	0.431500
C	-3.402798	2.221708	0.586201
H	-4.104788	2.154674	1.400561
C	-3.463955	3.275146	-0.313048
H	-4.214481	4.043716	-0.201741
C	-2.567885	3.298645	-1.367281
H	-2.598294	4.063044	-2.127962
C	-1.603839	2.313031	-1.439799
H	-0.920388	2.300504	-2.264555
C	2.424050	1.253016	0.454025
N	0.037648	-0.004827	-2.435748
C	0.098864	-0.004349	-3.577931
C	0.179009	-0.008576	-5.015894
H	1.035569	-0.605799	-5.326292
H	0.298566	1.014642	-5.370707
H	-0.735611	-0.434270	-5.426870

$^{3}[Ni^{II}(Py5OH)MeCN]^{2+}$

62

Ni	0.000235	0.019232	0.628722
O	3.497801	-0.074334	-2.258622
H	4.342775	-0.061428	-1.795992
N	-0.002246	-0.049630	-1.488082
N	-1.603843	-1.375343	0.583886
O	-3.504160	-0.073346	-2.249337
H	-4.347826	-0.060771	-1.784274
N	-1.598751	1.412755	0.488374
N	1.598939	-1.381267	0.573760
N	1.601621	1.409231	0.486423
C	1.703369	2.473116	1.290723
H	0.998459	2.528097	2.102705
C	-1.696871	2.478545	1.290547
H	-0.989647	2.534493	2.100463
C	-2.654555	3.462492	1.123572
H	-2.688614	4.302299	1.800783
C	-3.544278	3.337356	0.068952
H	-4.294508	4.092814	-0.115641
C	-3.469812	2.216351	-0.746172
H	-4.154790	2.099052	-1.569269
C	-2.498278	1.256052	-0.493141
C	-2.442450	-0.043376	-1.328064
C	-1.158592	-0.071293	-2.160547
C	-1.202409	-0.115343	-3.545867
H	-2.149396	-0.131729	-4.056605
C	-0.005764	-0.136837	-4.243876
H	-0.007129	-0.170622	-5.323999
C	1.192667	-0.114583	-3.548936
H	2.138329	-0.130408	-4.062142
C	1.152436	-0.070870	-2.163504
C	2.438651	-0.044471	-1.334417
C	1.697454	-2.394562	1.441281
H	0.989449	-2.399689	2.252508
C	2.655841	-3.386513	1.336465
H	2.690397	-4.182445	2.064694
C	3.545736	-3.326839	0.276274
H	4.296816	-4.091545	0.139332
C	3.469996	-2.259754	-0.607998
H	4.154338	-2.195473	-1.437395
C	2.498387	1.253364	-0.497634
C	2.661881	3.456129	1.123367
H	2.698846	4.294406	1.802321
C	3.548643	3.332106	0.066114
H	4.299297	4.087040	-0.118889
C	3.470587	2.212925	-0.751152
H	4.153172	2.096602	-1.576365
C	-2.501312	-1.283400	-0.407628
C	-3.474016	-2.257009	-0.596706
H	-4.157032	-2.195668	-1.427440
C	-3.552102	-3.320061	0.292220
H	-4.303207	-4.085067	0.157098
C	-2.665212	-3.374799	1.355240
H	-2.702811	-4.166584	2.087825
C	-1.705938	-2.383400	1.457153
H	-1.000814	-2.383868	2.271132
C	2.497601	-1.286329	-0.416456
N	0.005652	0.085771	2.704293
C	0.013065	0.129487	3.849210
C	0.023559	0.186695	5.293431
H	0.902343	0.734569	5.630270
H	0.051590	-0.824110	5.697811
H	-0.875035	0.691292	5.645219
